# Effectiveness of a culturally appropriate intervention to prevent intimate partner violence and HIV transmission among men, women, and couples in rural Ethiopia: Findings from a cluster-randomized controlled trial

**DOI:** 10.1371/journal.pmed.1003274

**Published:** 2020-08-18

**Authors:** Vandana Sharma, Jessica Leight, Fabio Verani, Samuel Tewolde, Negussie Deyessa

**Affiliations:** 1 Abdul Latif Jameel Poverty Action Lab, Massachusetts Institute of Technology, Cambridge, Massachusetts, United States of America; 2 International Food Policy Research Institute, Washington, DC, United States of America; 3 CARE, New York, United States of America; 4 EngenderHealth, Addis Ababa, Ethiopia; 5 Ethiopian Public Health Association, Addis Ababa, Ethiopia; 6 Addis Ababa University, Department of Preventive Medicine, School of Public Health, Addis Ababa, Ethiopia; Harvard Medical School, UNITED STATES

## Abstract

**Background:**

Intimate partner violence (IPV) is associated with increased HIV risk and other adverse health and psychosocial outcomes. We assessed the impact of Unite for a Better Life (UBL), a gender-transformative, participatory intervention delivered to men, women, and couples in Ethiopia in the context of the coffee ceremony, a traditional community-based discussion forum.

**Methods and findings:**

Villages (n = 64) in 4 Ethiopian districts were randomly allocated to control, men’s UBL, women’s UBL, or couples’ UBL, and approximately 106 households per village were randomly selected for inclusion in the trial. The intervention included 14 sessions delivered twice weekly by trained facilitators; control arm households were offered a short IPV educational session. Primary outcomes were women’s experience of past-year physical or sexual IPV 24 months postintervention. Secondary outcomes included male perpetration of past-year physical or sexual IPV, comprehensive HIV knowledge, and condom use at last intercourse. Additional prespecified outcomes included experience and perpetration of past-year physical and/or sexual IPV and emotional IPV, HIV/AIDs knowledge and behaviors, decision-making, and gender norms. An intention-to-treat (ITT) analysis was conducted, evaluating 6,770 households surveyed at baseline in 2014–2015 (1,680 households, 16 clusters in control; 1,692 households, 16 clusters in couples’ UBL; 1,707 households, 16 clusters in women’s UBL; 1,691 households, 16 clusters in men’s UBL). Follow-up data were available from 88% of baseline respondents and 87% of baseline spouses surveyed in 2017–2018. Results from both unadjusted and adjusted specifications are reported, the latter adjusting for age, education level, marriage length, polygamy, socioeconomic status, and months between intervention and endline. For primary outcomes, there was no effect of any UBL intervention compared to control on women’s past-year experience of physical (couples’ UBL arm adjusted odds ratio [AOR] = 1.00, 95% confidence interval [CI]: 0.77–1.30, p = 0.973; women’s UBL arm AOR = 1.11, 95% CI 0.87–1.42, p = 0.414; men’s UBL arm AOR = 1.02, 95% CI: 0.81–1.28, p = 0.865) or sexual IPV (couples’ UBL arm AOR = 0.86, 95% CI: 0.62–1.20, p = 0.378; women’s UBL arm AOR = 1.15, 95% CI: 0.89–1.50; p = 0.291; men’s UBL arm AOR = 0.80, 95% CI: 0.63–1.01, p = 0.062). For the secondary outcomes, only the men’s UBL intervention significantly reduced male perpetration of past-year sexual IPV (AOR: 0.73; 95% CI: 0.56–0.94, p = 0.014), and no intervention reduced perpetration of past-year physical IPV. Among women, the couples’ UBL intervention significantly improved comprehensive HIV knowledge, and both couples’ and women’s UBL significantly increased reported condom use at last intercourse. Among additional outcomes of interest, the men’s UBL intervention was associated with a significant reduction in women’s experience of past-year physical and/or sexual IPV (AOR = 0.81, 95% CI: 0.66–0.99, p = 0.036) and men’s perpetration of physical and/or sexual IPV (AOR = 0.78; 95% CI: 0.62–0.98, p = 0.037). UBL delivered to men and couples was associated with a significant reduction in HIV risk behaviors and more equitable intrahousehold decision-making and household task-sharing. The primary limitation is reliance on self-reported data.

**Conclusions:**

A gender-transformative intervention delivered to men was effective in reducing self-reported perpetration of sexual IPV but did not reduce IPV when delivered to couples or women. We found evidence of decreased sexual IPV with men’s UBL across men’s and women’s reports and of increased HIV knowledge and condom use at last intercourse among women. The men’s UBL intervention could help accelerate progress towards gender equality and combating HIV/AIDS.

**Trial registration:**

The trial was prospectively registered at clinicaltrials.gov (NCT02311699) and in the American Economic Association registry (AEARCTR-0000211).

## Abstract 2

In a cluster-randomized trial, Vandana Sharma and colleagues investigate the impact of the gender-transformative Unite for a Better Life (UBL) intervention on intimate partner violence, HIV high-risk behaviors, and household decision making when delivered and assessed among groups of women, men, and couples in Ethiopia.

## Author summary

**Why was this study done?:**

- Intimate partner violence (IPV) experienced by women is a major global health challenge with immediate and long-term adverse health, social, and economic consequences.
- Group-based interventions to prevent IPV by targeting underlying gender and social norms have shown promise, but there is limited evidence on interventions for couples and men.

**What did the researchers do and find?:**

- We conducted a cluster-randomized controlled trial (cRCT) to evaluate the effectiveness of Unite for a Better Life (UBL), a participatory, gender-transformative IPV and HIV prevention intervention delivered to groups of women, men, or couples. The UBL program is delivered in the context of the Ethiopian traditional coffee ceremony, a forum for community-based discussion.
- The trial included a sample of 64 villages in southern Ethiopia randomly assigned to one of 4 trial arms: women’s UBL, men’s UBL, couples’ UBL, or a control group. The baseline sample included 6,770 respondents randomly selected within the study villages, and follow-up data were available from 88% of baseline respondents and 87% of their spouses.
- Women’s past-year experience of IPV and men’s past-year perpetration of IPV, along with comprehensive knowledge on HIV and condom use at last intercourse, were assessed.
- Compared to the control group, the UBL intervention was effective in reducing male perpetration of past-year sexual IPV in the men’s UBL arm. The men’s UBL program was also associated with reductions in women’s experience of past-year physical and/or sexual IPV and male perpetration of past-year physical and/or sexual IPV.
- In addition, all 3 UBL interventions were associated with positive effects on a range of other outcomes, including HIV risk behaviors, intrahousehold decision-making, and male involvement in household tasks.

**What do these findings mean?:**

- The findings suggest that UBL, when delivered to men, is an effective strategy for reducing IPV and highlight the relative effectiveness of working with men compared to couples and women in this context.
- The intervention demonstrates promise as a strategy that could be replicated and tested in other settings and that could help accelerate progress towards achieving gender equality and combating HIV/AIDS.

## Background

Globally, 30% of women experience physical and/or sexual violence by an intimate partner (IPV) in their lifetime [1]. IPV has both immediate and long-term adverse health, social, and economic consequences for women and their families [2,3]. Physical effects of IPV include traumatic injuries, chronic illness, death [2,3], and adverse mental health effects, including depression, anxiety, and suicide [3,4]. In addition, IPV is linked with poor reproductive and sexual health outcomes, as well as increased HIV risk [5–7].

Gender inequalities are key drivers of both IPV and HIV; social norms that reinforce men’s power vis-à-vis their female partners contribute to violence against women and reduce women’s ability to negotiate safe sexual relationships and seek protection from abuse [7]. In addition, there is a higher prevalence of high-risk sexual behavior among male perpetrators of IPV [8]. Research suggests that sub-Saharan Africa, the region most affected by HIV/AIDs, has some of the highest levels of IPV [9,10], including in Ethiopia, where the lifetime prevalence of physical and/or sexual IPV among women is over 70% [10].

A growing number of interventions to prevent and reduce IPV have been rigorously evaluated in sub-Saharan Africa [11], including several interventions that simultaneously target HIV risk [12–14]. Group-based participatory education interventions that address the underlying gender and social norms that contribute to IPV and build skills to support healthy relationships have shown promise [11], and these interventions have generally targeted women. However, male engagement has been noted as critical to the goal of IPV prevention [15], and several studies suggest that promotion of equitable behaviors among men may reduce perpetration of IPV [16–18] and women’s experience of IPV [19]. In addition, qualitative analyses suggest that working with couples may be an effective strategy in IPV prevention [20,21]. Yet, the available body of evidence on men’s and couples’ interventions remains thin. There is also limited evidence on the effect of IPV interventions on a more diverse range of outcomes linked to household gender and power dynamics, such as equitable decision-making and participation in household tasks.

The Unite for a Better Life (UBL) program is a gender-transformative, participatory intervention delivered to men, women, and couples in Ethiopia in the context of the coffee ceremony, a traditional forum for community-based discussion. The program aims to reduce physical and sexual IPV and HIV risk behaviors as well as promote healthier, more equitable relationships. We assessed the program’s effect on women’s past-year experience of physical or sexual IPV, past-year male perpetration of physical or sexual IPV, HIV risk behaviors, and household gender and power dynamics and task-sharing.

## Methods

### Study design

This study was a 4-arm, cluster-randomized controlled trial (cRCT) conducted between December 2014 and March 2018 in rural Ethiopia. In the 2005 WHO Multi-country Study on Women’s Health and Domestic Violence, Ethiopia reported the highest prevalence of IPV in any country surveyed; over 70% of women reported lifetime physical and/or sexual IPV [10]. In addition, the HIV prevalence is 1.2% among women and 0.6% among men [22], but HIV knowledge levels remain low; 20% of women and 38% of men reported comprehensive HIV knowledge in the 2016 Ethiopia Demographic and Health Survey (DHS) [22].

The UBL trial was implemented by the Abdul Latif Jameel Poverty Action Lab (J-PAL) at the Massachusetts Institute of Technology (MIT), in partnership with the Addis Ababa University (AAU) School of Public Health, the Ethiopian Public Health Association (EPHA), and EngenderHealth. Because the intervention was designed for groups of individuals, a village-level cluster design was employed. Sixty-four villages (kebeles) in 4 districts (Mareko, Meskan, Silte, and Sodo) in the Gurague zone of the Southern Nations, Nationalities and People’s Region (SNNPR) were randomly selected for inclusion from the sampling frame of all villages within these districts. Villages were then randomly assigned to one of the 4 study arms (women’s UBL, men’s UBL, couples’ UBL, control) using a parallel randomization design, with an equal allocation ratio and stratification at the district level.

In addition, a second individual-level randomization was conducted. In each village within the 3 treatment arms (n = 48 villages), 80% of individuals enrolled in the trial were sampled to participate in UBL. The remaining 20% were included in baseline and endline data collection only for assessment of intervention spillover effects. In all study villages, data were collected from enrolled individuals at baseline and from enrolled individuals and their spouses at endline, approximately 24 months postintervention.

### Ethics approval and consent to participate

The study protocol was approved by the Committee on the Use of Humans as Experimental Subjects (COUHES) at MIT (protocol number 1211005333) and by the Institutional Review Board at the AAU College of Health Sciences (protocol number 044/12/SPH). The trial was prospectively registered on clinicaltrials.gov (NCT02311699), and in the American Economic Association (AEA) registry (AEARCTR-0000211). A community advisory board comprising ND, key stakeholders, and representatives from study districts convened regularly for supervision and adverse event monitoring. Verbal informed consent was obtained from all participants.

### Participants

All households with a married or cohabiting couple in which the woman was between 18 and 49 years were eligible for inclusion in the trial. Within sampled villages, one subvillage (gotte) was selected via simple random sampling; subvillages without health extension workers (HEWs) were excluded from the sampling frame. If a subvillage did not have an adequate sample size, the most proximate subvillage was added to create one sampling unit. Within each sampling unit, 106 households were randomly selected using the household roster maintained by HEWs and replaced if ineligible when screened. In polygamous households, one woman was selected via simple random sampling.

### Randomization and masking

Random assignment of villages to study arms was conducted using a random number assigned in Stata version 12.0 using a reproducible seed and included district-level stratification. Randomization was conducted by the principal investigators, and allocation by cluster number and name was communicated to the field team. Blinding of sampled individuals in treatment communities was not possible because they were informed of their treatment assignment when invited to participate in the intervention. Individuals in control communities may have been blind to their inclusion in the trial. Data collection staff were blind to treatment assignment at baseline but at endline may have observed materials (workbooks, attendance sheets) linked to the intervention.

Given that ethical recommendations on IPV research state men and women in the same households should not be interviewed about IPV, a second within-village randomization assigned households to a “male survey” subarm or “female survey” subarm at baseline [23]. In each subarm, the specified individual (male or female spouse) was surveyed at baseline, independent of the treatment assignment. Prior to endline, the feasibility of additionally interviewing spouses of baseline individuals was assessed through discussions with experts, local stakeholders, and the local IRB, as well as a review of recent couples research [24], and we concluded that we would be able to safely collect IPV data from both partners within households. The endline survey thus included all baseline individuals and their spouses. When a baseline respondent had multiple wives, simple random sampling was used to select one wife to participate in the endline survey. The team implementing the intervention was blind to the survey subarm assignment.

### Procedures

Data were collected from sampled participants at baseline from December 2014 to March 2015. Following the baseline survey and randomization, the UBL intervention was implemented between April 2015 and October 2015. Follow-up surveys were conducted with baseline respondents and their spouses between March 2017 and October 2017, approximately 24 months postintervention. To minimize attrition, additional endline data collection was conducted between January and March 2018.

#### The intervention

UBL is a gender-transformative intervention delivered within the context of the Ethiopian coffee ceremony, a culturally established forum for community discussion and conflict resolution. A gender-transformative approach addresses the root causes of gender-based inequalities by actively examining and changing inequitable gender norms and imbalances of power. Curricula designed for women, men, and couples were developed together with EngenderHealth, and all 3 curricula were pilot tested in nonstudy villages and refined prior to the trial.

The final curricula included 14 participatory and skills-building sessions (total 38 hours) led by 1 trained, same-sex facilitator for men’s and women’s UBL groups and 1 female and 1 male facilitator for couples’ groups to assist participants in identifying and transforming power imbalances within their relationships and to build skills for healthy, nonviolent, equitable relationships. Because traditionally, women prepare coffee during the ceremony, implementing the curriculum within the coffee ceremonies offered an opportunity to model and promote more equitable behaviors and also increased the cultural relevance of the program. The intervention curricula included specific instructions for when and how to prepare the coffee during each session. Two participants were selected at the end of each session to lead each coffee ceremony for the next session. They would be responsible for preparing the coffee at the start of the session in the venue, as well as pouring it and serving it to all participants. Since the traditional coffee ceremony typically involves brewing 3 cups of coffee per person over the course of several hours, the participants would continue to serve the coffee at the designated time points within each session.

In intervention groups in which men participated in the sessions, either 2 men (in the men’s groups) or a couple (in the couple’s groups) would be selected to lead the next coffee ceremony. In both of these intervention arms, the facilitators would model preparing and serving the coffee in the first 2 sessions prior to requesting that participants manage this responsibility for subsequent sessions. In the women’s arm, 2 female participants would prepare the coffee in each session. The facilitators were carefully trained in how to lead the coffee ceremonies in order to increase the comfort of all participants and set a context for an open and productive discussion by all parties. Part of this training specifically highlighted their role in encouraging both men and women to participate fully in the coffee ceremony in all contexts (gender-segregated or gender-mixed).

UBL was delivered by AAU and the EPHA in twice-weekly in-person sessions including approximately 20 sampled individuals per group in venues provided by each community (such as schools, health facilities, and community centers). Each session included a coffee ceremony in which 2 participants prepared and served the coffee and discussion and interactive activities focused on gender norms, sexuality, communication and conflict resolution, HIV/AIDS, and IPV. All participants, regardless of sex, took turns leading the coffee ceremonies. Table 1 provides further details on curricular content.

**Table 1:** UBL intervention session details.

Session Number	Session Title	Session Objectives
1	Program Introduction & Understanding Gender	To describe the goal of the program; to distinguish between sex and gender; to understand how gender norms affect couples' health and wellbeing
2	Act Like a Man/Act Like a Woman	To describe common gender norms for men and women; to understand how inequitable gender norms can contribute to negative health outcomes; to describe ways to challenge inequitable gender norms
3	Healthy Sexuality	To describe sexuality beyond intercourse and reproduction; to challenge common myths about sexuality; to describe the links between gender norms and individual sexual experience and expression
4	Healthy & Unhealthy Relationships	To describe healthy and unhealthy behaviors within relationships; to state important characteristics of healthy relationships
5	Power in Relationships (Couples’, Women's UBL) Expressing Emotions & Dealing with Anger (Men's UBL)	**Power in relationships:** To define power; to describe factors that contribute to having power and how power can be used; to describe ways to create balance of power within relationships **Expressing emotions & dealing with anger:** To identify differences in the ways men and women express emotions; to explain consequences of not expressing emotions; to identify and practice strategies for reacting constructively and nonviolently when angry
6	Joint Decision-Making	To explain the benefits of joint decision-making; to describe and apply 7 strategies for joint decision-making
7	Negotiating Men's & Women's Roles In & Outside of Home	To identify roles, responsibilities, and workload for men and women in the family; to compare how much time men and women spend caring for themselves and for others; to explore the implications of women's workloads on their health and wellbeing
8	Communicating with Your Partner—Active Listening	To describe communication and its 3 phases; to demonstrate 4 ways to be a good listener; to describe the benefits of being a good listener
9	Talking with Your Partner about Preventing HIV	To understand HIV and STI transmission, prevention, and treatment; to explain the links between inequitable gender norms and HIV vulnerability; to describe the levels of HIV risk associated with various sexual behaviors; to describe the benefits of getting tested for HIV; to be able to demonstrate how to correctly put a condom on a penile model
10	What Is Violence?	To describe violence; to identify forms of violence against women; to identify impact of violence against women on couples, families, and communities; to identify alternatives to violence
11	Setting Personal Boundaries in Relationships & Sexual Consent	To understand own personal boundaries; to define sexual consent; to be able to use assertive communication to consent or not consent to sexual activity
12	Nonviolent Ways to Resolve Conflict	To explain how childhood exposure to conflict influences conflict negotiation style as an adult; to identify 5 fair arguing rules; to be able to make a complaint using an assertive communication style
13	Understanding Violence & Supporting Survivors	To understand the cycle of violence and how it influences help-seeking; to be able to address men who behave violently; to support women who have experienced violence; to identify IPV services in the community; to identify barriers in accessing services
14	Empowering Change & Program Closure	To challenge violence in safe ways; to be able to provide support to those experiencing violence; to identify key learning s from the course and make personal commitments for the future

**Abbreviations:** IPV, intimate partner violence; STI, sexually transmitted infection; UBL, Unite for a Better Life.

Female facilitators moderated women’s groups, male facilitators moderated men’s groups, and 1 male and 1 female facilitator jointly moderated each couples’ group. Facilitators also recorded participants’ attendance and conducted brief postsession questionnaires with 2 participants following each session. Participants received an in-kind incentive (for example, cooking oil, sugar, or spaghetti) valued at approximately $4 USD following full attendance at each set of 4 sessions.

Forty-eight male and female facilitators were recruited from the districts and trained in 2 phases. First, during intervention piloting, they engaged as participants completing all 14 sessions led by master trainers. This enabled facilitators to learn the curriculum, observe high-quality facilitation, and critically examine their own assumptions around gender, sexuality, and IPV. Second, they completed a 10-day facilitator training on participatory learning, facilitation skills, and safety procedures. During implementation, the intervention coordinator (ST) observed sessions and provided ongoing feedback to facilitators to ensure intervention fidelity.

The intervention was first implemented in Meskan and Mareko districts (April to June 2015) and second in Silte and Sodo districts (August to October 2015). Women and men in the control group received a short educational session on IPV and HIV/AIDS prevention.

This study is reported as per the Consolidated Standards of Reporting Trials (CONSORT) guideline (S1 Table) and the intervention as per the Template for Intervention Description and Replication (TIDieR) checklist (S2 Table).

#### Data collection

Baseline and endline data were collected using paper surveys by trained male and female Amharic-speaking enumerators from the study areas. Male respondents were administered questionnaires by male enumerators, and female respondents were administered questionnaires by female enumerators. Verbal consent was obtained and questionnaires were administered in confidential settings, following WHO ethical guidelines for IPV research [18]. Enumerators completed a 4-week training on survey administration, ethics, interviewing skills, and safety and referral protocols and were supervised by a team of supervisors and the local researcher. A list of local medical, legal, and other relevant support services was given to respondents, and referrals for psychological support were provided.

The questionnaire was adapted from the WHO Multi-country Study on Women’s Health and Domestic Violence questionnaire [10] and included modules on sociodemographic information, gender norms and attitudes, household decision-making and task-sharing, HIV, and IPV. An abridged questionnaire was administered at endline to the spouses of baseline respondents because of resource constraints. Questionnaires are available in S3–S6 Text.

### Outcomes

Three sets of outcomes are included: primary outcomes prespecified in the clinical trials registry, secondary outcomes prespecified in the registry, and additional outcomes that were included in a preanalysis plan registered prior to analysis (S2 Text). We hypothesized that the UBL intervention would lead to reductions in IPV in the past 12 months but that it might not affect all forms of IPV in the same way. Therefore, 2 primary outcome measures were prespecified: past-year experience of physical IPV and past-year experience of sexual IPV, both reported by women. Two secondary IPV outcomes were prespecified: past-year male perpetration of physical IPV and past-year male perpetration of sexual IPV. Perpetration of physical or sexual IPV were designated as secondary outcomes since male reported perpetration has been shown to be less reliable than women’s reported experience of IPV in some settings [23,25], and there were limited IPV perpetration data from Ethiopia [26]. However, it should be noted that baseline questionnaire pretesting in the study area did not suggest similar differences in reporting between women and men in this context.

Additional IPV variables prespecified in the preanalysis plan prior to analysis (but not in the trial registry) include past-year experience and perpetration of emotional IPV and composite measures capturing past-year experience and perpetration of physical and/or sexual IPV.

Non-IPV prespecified secondary outcomes included comprehensive HIV/AIDS knowledge and condom use at high-risk sexual intercourse. The latter was deemed infeasible given the low levels of reported high-risk sexual intercourse among married couples within this population; accordingly, we analyzed condom use at last intercourse. Additional outcomes prespecified in the preanalysis plan prior to analysis, but not in the trial registry, include other HIV-related attitudes and behaviors, as well as household task-sharing, decision-making, and gender norms.

When the study design was modified to include additional data collection with spouses of baseline respondents at endline, it enabled post hoc analysis of additional outcomes of household-level IPV combining men’s and women’s reports of past-year IPV within a household. The household-level IPV variables capture any act of violence reported as being experienced or perpetrated by either spouse. Table 2 summarizes the key outcome measures assessed.

**Table 2:** Key outcome measures.

Variable	Respondent	Indicator	Coding
**Experience of IPV**			
Experienced physical violence from partner in past 12 months*	Women	Women were asked 6 items adapted from the WHO multicountry study [10] regarding whether their partner had ever done the following in the past 12 months: 1) slapped you or threw something at you that could hurt you; 2) pushed or shoved you; 3) hit you with a fist or with something that could hurt you; 4) kicked you, dragged you, or beat you up; 5) choked or burned you on purpose; 6) threatened to use or actually used a gun, knife, or other weapon against you. Responses ranged from 0 = no, 1 = yes.	Binary; coded as 1 if responded yes to any of the 6 items and 0 if no to all.
Experienced sexual violence from partner in the past 12 months*	Women	Women were asked 3 items regarding whether their partner had ever done the following in the past 12 months: 1) physically force you to have sexual intercourse with him even when you did not want to; 2) force you to perform sexual acts that you did not want to; 3) did you ever have sexual intercourse because you were intimidated by him or afraid he would hurt you? Responses ranged from 0 = no, 1 = yes.	Binary; coded as 1 if responded yes to any of the 3 items and 0 if no to all.
Experienced physical and/or sexual violence from partner in the past 12 months***	Women	Includes the 6 physical violence items and 3 sexual violence items above.	Binary; coded as 1 if responded yes to any of the 9 items and 0 if no to all.
Experienced emotional violence from partner in the past 12 months***	Women	Women were asked 4 items adapted from the WHO multicountry study [10] regarding whether their partner had ever done the following in the past 12 months: 1) insulted you or made you feel bad about yourself: 2) belittled or humiliated you in front of other people; 3) done things to scare or intimidate you on purpose (for example, by the way he looked at you, by yelling, by smashing things); 4) threatened to hurt you or someone you care about. Responses ranged from 0 = no, 1 = yes.	Binary; coded as 1 if responded yes to any of the 4 items and 0 if no to all.
**Perpetration of IPV**			
Perpetrated physical violence against partner in past 12 months**	Men	Men were asked 6 items adapted from the WHO multicountry study [10] regarding whether they had ever done the following against their partner in the past 12 months: 1) slapped her or threw something at her that could hurt her; 2) pushed or shoved her; 3) hit her with a fist or with something that could hurt her; 4) kicked her, dragged her, or beat her up; 5) choked or burned her on purpose; 6) threatened to use or actually used a gun, knife, or other weapon against her. Responses ranged from 0 = no, 1 = yes.	Binary; coded as 1 if responded yes to any of the 6 items and 0 if no to all.
Perpetrated sexual violence against partner in the past 12 months**	Men	Men were asked 3 items regarding whether they had ever done the following to their partner in the past 12 months: 1) physically force her to have sexual intercourse with him even when she did not want to; 2) force her to perform sexual acts that she did not want to; 3) did she ever have sexual intercourse because she was intimidated by him or afraid he would hurt her? Responses ranged from 0 = no, 1 = yes.	Binary; coded as 1 if responded yes to any of the 3 items and 0 if no to all.
Perpetrated physical and/or sexual violence against partner in the past 12 months***	Men	Includes the 6 physical violence items and 3 sexual violence items above.	Binary; coded as 1 if responded yes to any of the 9 items and 0 if no to all.
Perpetrated emotional violence against partner in the past 12 months***	Men	Men were asked 4 items adapted from the WHO multicountry study [10] regarding whether they had ever done the following against their partner in the past 12 months: 1) insulted her or made her feel bad about yourself; 2) belittled or humiliated her in front of other people; 3) done things to scare or intimidate her on purpose (for example, by the way you looked at her, by yelling, by smashing things); 4) threatened to hurt her or someone she cares about. Responses ranged from 0 = no, 1 = yes.	Binary; coded as 1 if responded yes to any of the 4 items and 0 if no to all.
**HIV knowledge, attitudes, and behaviors**			
Comprehensive knowledge on HIV**	Women; men	Respondents were asked the following questions: 1) can people reduce their chance of getting the AIDS virus by having just one uninfected sex partner who has no other sex partners? 2) Can people get the AIDS virus from mosquito bites? 3) Can people reduce their chance of getting the AIDS virus by using a condom every time they have sex? 4) Can people get the AIDS virus because of witchcraft, God's curse, or other supernatural means? 5) Do you think that a healthy-looking person can have HIV? Responses ranged from 1 = yes, 2 = no, 3 = don't know.	Binary; coded as 1 if answered all questions correctly and 0 if one or more questions answered incorrectly.
Used condom at last intercourse**	Women; men	Respondents were asked: did you use a condom last time you had sex? Responses ranged for 0 = no, 1 = yes.	Binary; coded as 1 if used condom at last sex and 0 if did not use condom at last sex.
Confidence in ability to use a condom***	Women; men	Respondents were asked the following question: how confident are you that you know how to correctly use a condom? Responses ranged from 1 = not at all confident, 2 = somewhat confident, 3 = confident, 4 = very confident.	Binary; coded as 1 if responded confident or very confident, 0 if not at all confident or somewhat confident.
Ever been tested for HIV***	Women; men	Respondents were asked the following question: I don’t want to know the results, but have you ever had a blood test for HIV? Responses ranged from 0 = no, 1 = yes.	Binary; coded as 1 if have had an HIV test and 0 if never had an HIV test.
Discussed HIV risk with partner in the past 12 months***	Women; men	Respondents were asked if they have discussed HIV risk behavior with their partner in the last 12 months. Responses ranged from 0 = no, 1 = yes.	Binary; coded as 1 if have discussed HIV risk and 0 if have not discussed HIV risk.
Discussed sex with partner in the past 12 months***	Women; men	Respondents were asked if they have discussed sex with their partner in the last 12 months. Responses ranged from 0 = no, 1 = yes.	Binary; coded as 1 if have discussed sex and 0 if have not discussed sex.
**Knowledge, attitudes, and behaviors related to IPV**			
Knowledge of laws related to IPV***	Women; men	Respondents were asked 2 questions: 1) according to the law, is a husband who forces his wife to have sex against her will committing a criminal act (that is, the husband can be fined or put in jail)? 2) Are there any laws in your country about violence against women? Responses ranged from 0 = no, 1 = yes, 2 = don't know.	Binary; coded as 1 if responded correctly to both questions (yes to both questions).
Support for gender-equitable norms***	Women; men	Respondents were asked if they agreed with 12 gender-inequitable statements from the Gender Equitable Men's Scale: 1) a man should have the final word on decisions in his home; 2) a woman should obey her husband in all things; 3) it is alright for a man to beat his wife if she is unfaithful; 4) a man can hit his wife if she won't have sex with him; 5) a woman should not initiate sex; 6) a man should be outraged if his wife asks him to use a condom; 7) it is a woman's responsibility to avoid getting pregnant; 8) a woman who has sex before she marries does not deserve respect; 9) women should tolerate violence in order to keep their family together; 10) there are times a woman deserves to be beaten; 11) a man using violence against his wife is a private matter that shouldn't be discussed outside of the couple; 12) it disgusts me when I see a man acting like a woman. Responses ranged from 1 = agree, 2 = partially agree, 3 = do not agree.	A score was generated by summing the responses to all 12 questions. A binary variable generated and coded as 1 if responses totaled 24 or higher.
Do not believe that IPV is justified***	Women; men	Respondents were asked whether they believe a man has a good reason to beat his wife in the following situations: 1) she answers back to him; 2) she neglects taking care of the children; 3) she burns the food; 4) she goes out without telling him; 5) she refuses to have sex with him. Responses ranged from 1 = yes to 2 = no.	Binary; coded as 1 if responded no to all statements and coded as 0 if responded yes to any of the statements.
**Intrahousehold decision-making and gendered division of childcare and household tasks**			
Male involvement in household and childcare tasks***	Women; men	Respondents were asked how they divided 4 household tasks that are typically performed by women: 1) washing clothes; 2) cleaning the house; 3) preparing the food; 4) daily care of the children. Responses ranged from 1 = woman always does the task to 3 = shared equally or done together to 5 = man always does the task.	Binary; coded as 1 if man contributed to 2 or more tasks and 0 if contributed to fewer than 2 tasks.
Men's dominance in decision-making about food and clothing***	Women; men	Respondents were asked who in their household has the final say in how you spend money on food and clothing. Responses ranged from 1 = woman, 2 = man, 3 = both jointly, 4 = someone else.	Binary; coded as 1 if man has final say and as 0 if decision made by woman or made jointly.
Men's dominance in decision-making about purchase of large items***	Women; men	Respondents were asked who in their household has the final say in how you spend money on large investments such as a car or a house or a household appliance. Responses ranged from 1 = woman, 2 = man, 3 = both jointly, 4 = someone else.	Binary; coded as 1 if man has final say and as 0 if decision made by woman or made jointly.
Men's dominance in decision-making about spending time with family and friends***	Women; men	Respondents were asked who in their household has the final say regarding spending time with family or relatives. Responses ranged from 1 = woman, 2 = man, 3 = both jointly, 4 = someone else.	Binary; coded as 1 if man has final say and as 0 if decision made by woman or made jointly.

*Primary outcome measures.

**Secondary outcome measures.

***Additional outcomes not prespecified in trial registry but included in the preanalysis plan registered prior to analysis.

**Abbreviations:** IPV, intimate partner violence.

### Statistical analysis

Sample size calculations for past-year experience of IPV were conducted following Hayes and Bennett [27] and assuming K = 0.2, a type I error (alpha) of 0.10 and power (1-beta) of 0.8, a one-sided test for a 2-sample comparison of proportions, and 25% attrition at follow-up. Using the measured IPV prevalence reported by the WHO Multi-country Study on Women’s Health and Domestic Violence [10] and prevalence of male perpetration reported in Philpart and colleagues [26], the study was powered to detect a 25% decline in past-year experience of physical IPV, a 22% decrease in past-year experience of sexual IPV, a 31% decline in male perpetration of physical IPV, and a 30% decline in male perpetration of sexual IPV for comparisons between each experimental arm and the control arm. However, at endline, the sample size was doubled through the inclusion of spouses of baseline respondents; this change would allow the study to detect smaller reductions in these outcomes.

Women’s and men’s characteristics at baseline were compared using descriptive statistics. To estimate the effect of the intervention on outcomes measured at 24-month follow-up, an intention-to-treat (ITT) analysis was conducted with the 80% sample randomly selected for participation in UBL in the treatment villages and the full sample of participants in the control villages without imputation for missing respondents.

Additional post hoc analyses were also undertaken to assess intervention effects on IPV outcomes at the household level (at which men’s and women’s reports were combined) and among highly adherent respondents (women, men, or couples who attended at least 85% of UBL sessions). Adherence to the intervention was assessed via attendance data collected during each intervention session. Analyses were conducted with Stata version 13.1.

We use logistic regression models fitted with generalized estimating equations and robust standard errors to compare the control to each of the 3 treatment groups. Strata fixed effects for district are included, and standard errors are clustered at the level of the village. Odds ratios (ORs) and 95% confidence intervals (CIs) are reported for unadjusted and adjusted models; the latter included controls for respondent’s age, respondent’s education level, marriage length, polygamy, asset index, wealth quintile, whether they completed the full or short questionnaire at endline, and months between intervention end and endline data collection. When the outcome of interest is common (>10%) and the ORs are more than 2.5 or less than 0.5, they are adjusted using the method of Zhang and Zu [28] to approximate the risk ratio.

## Results

**Fig 1:**
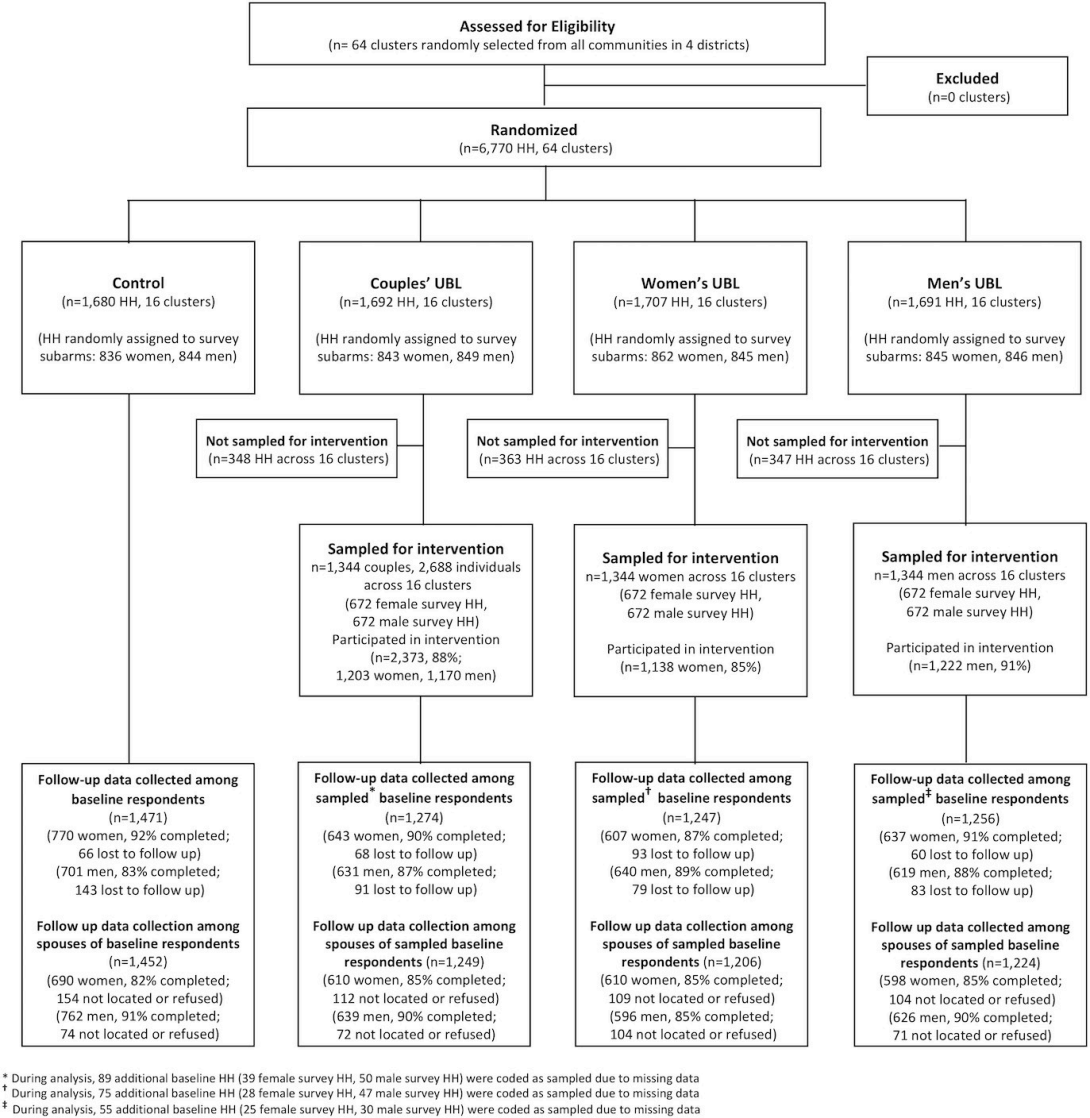
Participant flow diagram. Note: Records around sampling of households for intervention were missing in some intervention communities due to fieldworker error. Any households for which records were missing were coded as sampled for the intervention in order to generate conservative estimates of intervention effects. HH, household; UBL, Unite for a Better Life.

Between December 2014 and March 2015, 6,770 households across 64 randomly selected clusters were enrolled in the study (Fig 1). Random assignment of clusters to the 4 study arms yielded 1,680 households in 16 clusters assigned to the control group, 1,692 households in 16 clusters to the couples’ UBL group, 1,707 households in 16 clusters to the women’s UBL group, and 1,691 households in 16 clusters to the men’s UBL group. Baseline data from 1 spouse in each household were collected according to the study subarm assignment (in total, 3,386 women and 3,384 men). In the intervention arms, a total of 1,058 households were randomly selected for spillover assessment (348 in the couples’ arm, 363 in the women’s arm, and 347 in the men’s arm) and were not invited to participate in the intervention. No harms were reported.

Across the trial, the overall follow-up rate at 24 months among the respondents surveyed at baseline was 88% (87% among men, 90% among women), with the lowest follow-up rate among men in the control arm (83%). The overall follow-up rate for spouses of the baseline respondent at endline was 87% (85% among female spouses, 89% among male spouses). Differential loss to follow-up by arm or by sex was minimal. Reasons for loss to follow-up were primarily inability to find respondents because of relocation or respondent unavailability.

Baseline characteristics of women and men across the 4 study groups were broadly similar (Table 3). Women were on average 32 years of age, while men were 37 years. Roughly 75% of women had no formal schooling, and 16% of women reported being in polygamous relationships. Men and women reported on average 4 living children, and approximately 61% of households were Muslim.

**Table 3:** Baseline characteristics of women (N = 2,944) and men (N = 2,987) in the study sample by treatment arm.

	Women				Men			
	Control Group (N = 836)	Couples' UBL (N = 711)	Women's UBL (N = 700)	Men's UBL (N = 697)	Control Group (N = 844)	Couples' UBL (N = 722)	Women’s UBL (N = 719)	Men's UBL (N = 702)
**Respondent's age category**								
<30 years	304 (36.4)	266 (37.4)	249 (35.6)	259 (37.2)	137 (16.2)	144 (19.9)	129 (17.9)	120 (17.1)
30–39 years	386 (46.2)	308 (43.3)	315 (45.0)	310 (44.5)	350 (41.5)	308 (42.7)	313 (43.5)	281 (40.0)
>39 years	146 (17.5)	137 (19.3)	136 (19.4)	128 (18.4)	357 (42.3)	270 (37.4)	277 (38.5)	302 (43.0)
**Spouse's age category**								
<30 years	62 (7.4)	64 (9.0)	59 (8.4)	63 (9.0)	375 (44.4)	344 (47.7)	304 (42.3)	304 (43.3)
30–39 years	319 (38.2)	258 (36.3)	254 (36.3)	257 (36.9)	315 (37.3)	269 (37.3)	323 (44.9)	294 (41.8)
>39 years	455 (54.4)	389 (54.7)	387 (55.3)	377 (54.1)	154 (18.3)	109 (15.1)	92 (12.8)	105 (14.9)
**Respondent's level of education**								
None	641 (76.7)	544 (76.5)	556 (79.4)	535 (76.9)	311 (37.0)	287 (39.8)	278 (38.7)	300 (42.8)
Primary	182 (21.8)	157 (22.1)	139 (19.9)	150 (21.6)	499 (59.3)	397 (55.1)	410 (57.0)	367 (52.4)
Secondary	12 (1.4)	9 (1.3)	5 (0.7)	10 (1.4)	27 (3.2)	28 (3.9)	28 (3.9)	25 (3.6)
Higher	1 (0.1)	1 (0.1)	0 (0.0)	1 (0.1)	4 (0.5)	9 (1.3)	3 (0.4)	9 (1.3)
**Spouse's level of education**								
None	348 (41.7)	365 (51.3)	341 (48.7)	321 (46.1)	585 (69.5)	464 (64.5)	493 (68.7)	479 (68.2)
Primary	426 (51.1)	308 (43.3)	326 (46.6)	339 (48.6)	240 (28.5)	248 (34.4)	215 (29.9)	214 (30.5)
Secondary	49 (5.9)	29 (4.1)	29 (4.1)	34 (4.9)	14 (1.7)	8 (1.1)	10 (1.4)	9 (1.3)
Higher	11 (1.32)	9 (1.3)	4 (0.6)	3 (0.4)	3 (0.4)	0 (0.0)	0 (0.0)	0 (0.0)
**Religion**								
Muslim	485 (58.2)	509 (71.7)	400 (57.4)	418 (60.2)	468 (55.7)	518 (72.0)	420 (58.4)	435 (61.9)
Orthodox	279 (33.5)	183 (25.8)	249 (35.7)	202 (29.1)	311 (37.0)	177 (24.6)	233 (32.4)	231 (32.9)
Protestant	57 (6.8)	9 (1.3)	32 (4.6)	66 (9.5)	45 (5.4)	13 (1.8)	49 (6.8)	29 (4.1)
Catholic	12 (1.4)	9 (1.3)	16 (2.3)	8 (1.2)	16 (1.9)	11 (1.5)	17 (2.4)	8 (1.1)
Other	0 (0.0)	0 (0.0)	0 (0.0)	0 (0.0)	1 (0.1)	0 (0.0)	0 (0.0)	0 (0.0)
**Polygamous household**								
Yes	124 (14.8)	129 (18.1)	105 (15.0)	106 (15.2)	56 (6.6)	52 (7.2)	42 (5.8)	44 (6.3)
**Marriage length**								
<1 year	8 (1.0)	2 (0.3)	4 (0.6)	1 (0.1)	67 (7.9)	67 (7.9)	72 (10.0)	58 (8.3)
1–5 years	84 (10.1)	96 (13.5)	77 (11.0)	101 (14.5)	652 (77.3)	569 (78.8)	537 (74.7)	527 (75.0)
5–10 years	168 (20.1)	105 (14.8)	139 (19.9)	138 (19.8)	91 (10.8)	58 (8.0)	77 (10.7)	66 (9.4)
10–15 years	188 (22.5)	143 (20.1)	142 (20.3)	123 (17.7)	5 (0.6)	5 (0.7)	4 (0.6)	3 (0.4)
>15 years	388 (46.4)	365 (51.3)	338 (48.3)	334 (47.9)	29 (3.4)	23 (3.2)	29 (4.0)	49 (7.0)
**Number of living children**								
0	26 (3.1)	21 (3.0)	23 (3.3)	18 (2.6)	52 (6.2)	56 (7.8)	54 (7.5)	40 (5.7)
1	72 (8.6)	70 (9.9)	63 (9.0)	85 (12.2)	84 (10.0)	84 (11.6)	68 (9.5)	74 (10.5)
2–3	222 (26.6)	174 (24.5)	179 (25.6)	179 (25.7)	241 (28.6)	159 (22.0)	176 (24.5)	161 (22.9)
4+	516 (61.7)	446 (62.7)	435 (62.1)	415 (59.5)	467 (55.3)	423 (58.6)	421 (58.6)	428 (60.9)
**Asset index**								
<2	128 (15.3)	139 (19.6)	137 (19.6)	130 (18.7)	148 (17.5)	105 (14.5)	147 (20.5)	133 (18.9)
2–4	387 (46.3)	330 (46.4)	330 (47.1)	345 (49.5)	400 (47.4)	343 (47.5)	322 (44.8)	350 (49.8)
5–6	185 (22.1)	159 (22.4)	142 (20.3)	146 (21.0)	224 (25.5)	197 (27.3)	173 (24.1)	157 (22.3)
>6	136 (16.3)	83 (11.7)	91 (13.0)	74 (10.9)	72 (8.5)	77 (10.7)	77 (10.7)	63 (9.0)
**Wealth quintile**								
1^st^ (poorest)	454 (54.4)	421 (59.4)	423 (60.4)	458 (65.7)	518 (61.5)	432 (60.0)	458 (63.8)	446 (63.8)
2^nd^	0 (0.0)	0 (0.0)	0 (0.0)	0 (0.0)	0 (0.0)	0 (0.0)	0 (0.0)	0 (0.0)
3^rd^	33 (4.0)	36 (5.1)	36 (5.1)	17 (2.4)	0 (0.0)	0 (0.0)	0 (0.0)	0 (0.0)
4^th^	290 (34.8)	199 (28.1)	203 (29.0)	177 (25.4)	254 (30.2)	231 (32.1)	205 (28.5)	188 (26.9)
5^th^ (wealthiest)	57 (6.8)	57 (8.0)	38 (5.4)	45 (6.5)	70 (8.3)	57 (7.9)	56 (7.8)	65 (9.3)

Note that at baseline, only one respondent per household was interviewed according to study subarm assignment. **Abbreviations:** UBL, Unite for a Better Life.

Table 4 presents IPV outcomes by treatment arm among men and women. Crude and adjusted odds ratios (AORs) and 95% CIs are presented for each outcome comparing the prevalence in each intervention arm versus the control arm as per the ITT analysis. Adjusted models include controls for respondent’s age, respondent’s education level, marriage length, polygamy, asset index, wealth quintile, whether they completed the full or short questionnaire at endline, and months between intervention end and endline data collection. At follow-up, IPV prevalence was high: 20% of women in the control arm reported experiencing physical IPV in the last year, 37% reported sexual IPV in the last year, and 43% reported any physical and/or sexual IPV in the last year. Reported prevalence of male perpetration in the control arm was similar. The intracluster correlation at 24 months in the control arm for past-year experience of physical IPV in the control was 0.01 (95% CI: 0.00–0.03) and for past-year experience of sexual IPV was 0.07 (95% CI: 0.02–0.13). The intracluster correlation at 24 months for past-year perpetration of physical IPV was 0.07 (95% CI: 0.01–0.12) and for past-year perpetration of sexual IPV was 0.10 (95% CI: 0.03–0.16).

**Table 4:** Effect of the UBL intervention on primary and secondary IPV outcomes among women and men at 24-month follow-up: ITT analysis.

	Summary Statistics				Intervention Effect					
	Control Group	Couples' UBL	Women's UBL	Men's UBL	Couples' UBL		Women’s UBL		Men's UBL	
	N (%)	N (%)	N (%)	N (%)	OR	AOR*	OR	AOR*	OR	AOR*
**Primary IPV outcomes**										
**Experience of IPV—Women’s reports**										
Past-year physical IPV	292/1,452 (20.1)	255/1,249 (20.4)	267/1,211 (22.1)	268/1,233 (21.7)	1.01 (0.79–1.28), p = 0.940	1.00 (0.77–1.30), p = 0.973	1.11 (0.88–1.42), p = 0.379	1.11 (0.87–1.42), p = 0.414	1.10 (0.89–1.35), p = 0.386	1.02 (0.81–1.28), p = 0.865
Past-year sexual IPV	542/1,451 (37.4)	424/1,248 (34.0)	494/1,212 (40.8)	430/1,228 (35.0)	0.86 (0.65–1.15), p = 0.310	0.86 (0.62–1.20), p = 0.378	1.14 (0.91–1.44), p = 0.255	1.15 (0.89–1.50), p = 0.291	0.88 (0.72–1.08), p = 0.231	0.80 (0.63–1.01), p = 0.062
**Secondary IPV outcomes**										
**Perpetration of IPV—Men’s reports**										
Past-year physical IPV	313/1,459 (21.5)	272/1,268 (21.5)	309/1,230 (25.1)	242/1,244 (19.5)	0.98 (0.70–1.36), p = 0.884	0.97 (0.70–1.35), p = 0.866	1.23 (0.90–1.68), p = 0.192	1.21 (0.88–1.67), p = 0.232	0.87 (0.67–1.12), p = 0.281	0.85 (0.65–1.09), p = 0.200
Past-year sexual IPV	427/1,459 (29.3)	347/1,268 (27.4)	382/1,229 (31.1)	303/1,244 (24.4)	0.87 (0.62–1.24), p = 0.447	0.87 (0.61–1.25), p = 0.462	1.08 (0.86–1.36), p = 0.506	1.07 (0.84–1.37), p = 0.570	0.76 (0.59–0.97), p = 0.026	0.73 (0.56–0.94), p = 0.014
**Additional IPV outcomes**										
**Experience of IPV—Women’s reports**										
Past-year physical and/or sexual IPV	627/1,435 (43.2)	496/1,249 (39.7)	549/1,211 (45.3)	497/1,230 (40.4)	0.86 (0.67–1.11), p = 0.257	0.87 (0.65–1.15), p = 0.326	1.08 (0.86–1.36), p = 0.509	1.09 (0.84–1.40), p = 0.525	0.88 (0.73–1.05), p = 0.142	0.81 (0.66–0.99), p = 0.036
Past-year emotional IPV	886/1,460 (60.7)	741/1,253 (59.1)	712/1,217 (58.5)	717/1,236 (58.0)	0.92 (0.67–1.26), p = 0.599	0.92 (0.65–1.31), p = 0.655	0.90 (0.67–1.20), p = 0.464	0.90 (0.66–1.23), p = 0.499	0.88 (0.69–1.13), p = 0.318	0.81 (0.62–1.05), p = 0.114
**Perpetration of IPV—Men’s reports**										
Past-year physical and/or sexual IPV	566/1,459 (38.8)	462/1,268 (36.4)	526/1,229 (42.8)	430/1,244 (34.6)	0.87 (0.63–1.20), p = 0.397	0.87 (0.62–1.21), p = 0.406	1.18 (0.94–1.49), p = 0.151	1.17 (0.91–1.50), p = 0.212	0.81 (0.64–1.03), p = 0.080	0.78 (0.62–0.98), p = 0.037
Past-year emotional IPV	819/1,463 (56.0)	711/1,270 (56.0)	749/1,236 (60.6)	695/1,246 (55.8)	0.98 (0.75–1.28), p = 0.890	0.99 (0.76–1.29), p = 0.922	1.21 (0.94–1.56), p = 0.134	1.20 (0.93–1.55), p = 0.168	0.98 (0.78–1.23), p = 0.886	0.97 (0.78–1.22), p = 0.801

**Abbreviations:** AOR, adjusted OR; IPV, intimate partner violence; ITT, intention to treat; OR, odds ratio; UBL, Unite for a Better Life.

*Adjusted for respondent’s age, respondent’s schooling category, marriage length, polygamous household, socioeconomic status, whether completed the full or short survey at endline, and number of months between end of intervention and endline interview.

For the primary IPV outcomes, there was no effect of the intervention on experience of past-year physical IPV among women across any of the treatment arms (couples’ UBL arm AOR = 1.00, 95% CI: 0.77–1.30, p = 0.973; women’s UBL arm AOR = 1.11, 95% CI 0.87–1.42, p = 0.414; men’s UBL arm AOR = 1.02, 95% CI: 0.81–1.28, p = 0.865). However, there was a decline in past-year experience of sexual IPV among women in the men’s UBL arm that was marginally significant at the 10% level in the adjusted model only (men’s UBL arm AOR = 0.80, 95% CI: 0.63–1.01, p = 0.062), while no effects were observed in the couples’ or women’s arms (couples’ UBL arm AOR = 0.86, 95% CI: 0.62–1.20, p = 0.378; women’s UBL arm AOR = 1.15, 95% CI: 0.89–1.50, p = 0.291). For secondary IPV outcomes, the men’s UBL intervention significantly reduced male perpetration of past-year sexual IPV (AOR = 0.73; 95% CI: 0.56–0.94, p = 0.014), but there was no effect in the couples’ or women’s arms (couples’ UBL arm AOR = 0.87, 95% CI: 0.61–1.25, p = 0.462; women’s UBL arm AOR = 1.07, 95% CI: 0.84–1.37, p = 0.570). The intervention had no statistically significant effect on perpetration of physical IPV in any of the arms (couples’ UBL arm AOR = 0.97, 95% CI: 0.70–1.35, p = 0.866; women’s UBL arm AOR = 1.21, 95% CI: 0.88–1.67, p = 0.232; men’s UBL arm AOR = 0.85, 95% CI: 0.65–1.09, p = 0.200).

Non-IPV secondary outcomes are presented in Table 5 for women and in Table 6 for men. Among women, there were significant changes in both prespecified secondary HIV outcomes. The couples’ UBL intervention led to a statistically significant increase in comprehensive HIV knowledge among women (AOR = 1.56; 95% CI: 1.02–2.39, p = 0.040), but there were no effects in the men’s (AOR = 0.86; 95% CI: 0.54–1.37, p = 0.525) or women’s UBL arms (AOR = 1.26; 95% CI: 0.82–1.93, p = 0.300). There was also a significant increase in women’s reported condom use at last sexual intercourse in the women’s arm (AOR = 4.52; 95% CI: 1.85–11.01, p = 0.001) and in the couples’ arm (AOR = 3.12; 95% CI: 1.36–7.13, p = 0.007), but not in the men’s UBL arm (AOR = 1.35; 95% CI: 0.42–4.38, p = 0.615). For prespecified secondary HIV outcomes among men, there was no change in comprehensive HIV knowledge in any intervention arm (couples UBL arm AOR = 1.22; 95% CI: 0.83–1.79, p = 0.315; women’s UBL arm AOR = 1.28; 95% CI: 0.85–1.94, p = 0.238; men’s UBL arm AOR = 1.19; 95% CI: 0.86–1.66, p = 0.291). However, men reported increased condom use at last sexual intercourse in the couples’ UBL arm, though the effect is only marginally significant at the 0.1 alpha level (AOR: 1.80; 95% CI = 0.93–3.50, p = 0.082); there was no significant effect in the women’s (AOR: 1.20; 95% CI = 0.61–2.35, p = 0.600) or men’s UBL arm (AOR = 0.60; 95% CI = 0.25–1.45, p = 0.253).

**Table 5:** Effect of the UBL intervention on IPV knowledge, attitudes, norms, household task division and decision-making, and HIV outcomes among women: ITT analysis.

	Summary Statistics				Intervention Effect					
	Control Group	Couples' UBL	Women's UBL	Men's UBL	Couples' UBL		Women’s UBL		Men's UBL	
	N (%)	N (%)	N (%)	N (%)	OR	AOR*	OR	AOR*	OR	AOR*
**Secondary HIV outcomes**										
Comprehensive knowledge on HIV	95/770 (12.3)	116/643 (18.0)	92/607 (15.2)	71/637 (11.2)	1.58 (1.03–2.41), p = 0.036	1.56 (1.02–2.39), p = 0.040	1.27 (0.82–1.96), p = 0.285	1.26 (0.82–1.93), p = 0.300	0.88 (0.54–1.42), p = 0.604	0.86 (0.54–1.37), p = 0.525
Condom use at last intercourse	5/769 (0.65)	16/639 (2.5)	11/606 (1.8)	5/633 (0.8)	3.9 (1.62–9.42), p = 0.002	4.52 (1.85–11.01), p = 0.001	2.87 (1.31–6.30), p = 0.009	3.12 (1.36–7.13), p = 0.007	1.24 (0.39–3.90), p = 0.719	1.35 (0.42–4.38), p = 0.615
**Knowledge, attitudes, household decision-making, and task-sharing outcomes**										
**IPV knowledge, attitudes, norms**†										
Knowledge of IPV laws	413/770 (53.6)	364/643 (56.6)	353/607 (58.2)	381/637 (59.9)	1.13 (0.89–1.43), p = 0.312	1.12 (0.88–1.42), p = 0.352	1.21 (0.99–1.47), p = 0.058	1.20 (0.99–1.45), p = 0.056	1.28 (0.99–1.66), p = 0.053	1.31 (1.02–1.68), p = 0.034
Support for gender-equitable norms	305/770 (39.6)	281/643 (43.7)	280/607 (46.1)	236/637 (37.1)	1.18 (0.95–1.48), p = 0.138	1.21 (0.97–1.52), p = 0.092	1.29 (1.01–1.67), p = 0.045	1.32 (1.04–1.68), p = 0.020	0.89 (0.65–1.21), p = 0.455	0.89 (0.65–1.21), p = 0.455
Do not believe IPV is justified	299/770 (38.8)	277/643 (43.1)	266/607 (43.8)	237/637 (37.2)	1.20 (0.94–1.54), p = 0.144	1.21 (0.93–1.58), p = 0.150	1.22 (0.94–1.57), p = 0.140	1.22 (0.94–1.58), p = 0.137	0.93 (0.72–1.19), p = 0.554	0.93 (0.73–1.18), p = 0.537
**Household decision-making and division of childcare and household tasks**										
Male involvement (household & childcare tasks)	84/1,460 (5.8)	101/1,253 (8.1)	76/1,217 (6.2)	85/1,236 (6.9)	1.45 (1.06–2.00), p = 0.021	1.47 (1.08–2.01), p = 0.016	1.08 (0.72–1.62), p = 0.704	1.06 (0.70–1.61), p = 0.777	1.21 (0.88–1.68), p = 0.244	1.18 (0.84–1.66), p = 0.350
Male dominance in decision-making (food & clothing)	787/1,442 (54.6)	643/1,241 (51.8)	623/1,200 (51.9)	663/1,224 (54.2)	0.89 (0.70–1.14), p = 0.350	0.89 (0.70–1.13), p = 0.333	0.90 (0.73–1.11), p = 0.331	0.90 (0.73–1.05), p = 0.303	0.99 (0.79–1.24), p = 0.939	1.00 (0.80–1.25), p = 0.985
Male dominance in decision-making (large item purchases)	776/1,451 (53.5)	617/1,242 (49.6)	599/1,205 (49.7)	620/1,224 (50.7)	0.85 (0.67–1.09), p = 0.200	0.85 (0.66–1.08), p = 0.173	0.86 (0.69–1.07), p = 0.185	0.86 (0.69–1.07), p = 0.176	0.90 (0.72–1.12), p = 0.350	0.90 (0.73–1.12), p = 0.338
Male dominance in decision-making (spending time with family/friends)	608/1,453 (41.8)	512/1,245 (41.1)	496/1,206 (41.1)	540/1,227 (44.0)	0.96 (0.73–1.26), p = 0.775	0.95 (0.72–1.25), p = 0.726	0.97 (0.75–1.25), p = 0.801	0.96 (0.74–1.23), p = 0.731	1.10 (0.85–1.43), p = 0.460	1.13 (0.87–1.46), p = 0.368
**HIV knowledge, attitudes, behaviors**†										
Confidence in ability to use a condom	180/770 (23.4)	228/643 (35.5)	215/607 (35.4)	151/637 (23.7)	1.85 (1.35–2.52), p < 0.001	1.96 (1.45–2.67), p < 0.001	1.85 (1.26–2.74), p = 0.002	1.94 (1.31–2.88), p = 0.001	1.01 (0.72–1.41), p = 0.953	1.06 (0.77–1.45), p = 0.730
Been tested for HIV	564/768 (73.4)	512/643 (80.0)	465/605 (76.9)	509/635 (80.2)	1.41 (0.93–2.15), p = 0.108	1.48 (0.97–2.27), p = 0.069	1.19 (0.81–1.76), p = 0.370	1.24 (0.81–1.87), p = 0.320	1.45 (0.99–2.10), p = 0.053	1.66 (1.14–2.43), p = 0.009
Discussed HIV risk with partner	326/770 (42.3)	303/643 (47.1)	306/607 (50.4)	266/637 (41.8)	1.21 (0.86–1.70), p = 0.273	1.26 (0.90–1.76), p = 0.178	1.39 (1.04–1.84), p = 0.026	1.47 (1.09–1.99), p = 0.013	0.96 (0.73–1.27), p = 0.791	0.93 (0.72–1.20), p = 0.581
Discussed sex with partner	254/770 (33.0)	270/643 (42.0)	278/607 (45.8)	254/637 (39.9)	1.50 (1.09–1.98), p = 0.011	1.61 (1.20–2.15), p = 0.001	1.72 (1.28–2.32), p < 0.001	1.84 (1.37–2.48), p < 0.001	1.33 (1.02–1.73), p = 0.037	1.40 (1.08–1.81), p = 0.010

**Abbreviations:** AOR, adjusted OR; IPV, intimate partner violence; ITT, intention to treat; OR, odds ratio; UBL, Unite for a Better Life.

*Adjusted for respondent’s age, respondent’s schooling category, marriage length, polygamous household, socioeconomic status, whether completed the full or short survey at endline, and number of months between end of intervention and endline interview.

^†^Not assessed among spouses of baseline respondents in short endline questionnaire.

**Table 6:** Effect of the UBL intervention on IPV knowledge, attitudes, norms, household task division and decision-making, and HIV outcomes among men: ITT analysis.

	Summary Statistics				Intervention Effect					
	Control Group	Couples' UBL	Women's UBL	Men's UBL	Couples' UBL		Women’s UBL		Men's UBL	
	N (%)	N (%)	N (%)	N (%)	OR	AOR*	OR	AOR*	OR	AOR*
**Secondary HIV outcomes**										
Comprehensive knowledge on HIV	209/701 (29.8)	213/631 (33.8)	224/640 (35.0)	207/620 (33.4)	1.22 (0.82–1.80), p = 0.327	1.22 (0.83–1.79), p = 0.315	1.28 (0.82–1.97), p = 0.274	1.28 (0.85–1.94), p = 0.238	1.17 (0.82–1.67), p = 0.383	1.19 (0.86–1.66), p = 0.291
Condom use at last intercourse	17/697 (2.4)	29/629 (4.6)	19/636 (3.0)	9/617 (1.5)	1.92 (0.97–3.80), p = 0.061	1.80 (0.93–3.50), p = 0.082	1.22 (0.62–2.41), p = 0.565	1.20 (0.61–2.35), p = 0.600	0.59 (0.24–1.41), p = 0.231	0.60 (0.25–1.45), p = 0.253
**Knowledge, attitudes, household decision-making, and task-sharing outcomes**										
**IPV knowledge, attitudes, norms**†										
Knowledge of IPV laws	409/701 (58.4)	391/631 (62.0)	403/640 (63.0)	402/620 (64.8)	1.17 (0.90–1.53), p = 0.250	1.15 (0.87–1.51), p = 0.336	1.22 (0.99–1.52), p = 0.064	1.21 (0.96–1.52), p = 0.099	1.32 (1.08–1.63), p = 0.007	1.36 (1.10–1.68), p = 0.005
Support for gender-equitable norms	304/701 (43.4)	333/631 (52.8)	290/640 (45.3)	314/620 (50.7)	1.49 (1.08–2.05), p = 0.015	1.47 (1.06–2.03), p = 0.021	1.10 (0.78–1.54), p = 0.589	1.10 (0.78–1.53), p = 0.594	1.36 (0.99–1.87), p = 0.060	1.38 (1.01–1.89), p = 0.040
Do not believe IPV is justified	373/701 (53.2)	349/631 (55.3)	345 (53.9)	349 (56.3)	1.09 (0.75–1.60), p = 0.642	1.09 (0.74–1.61), p = 0.658	1.04 (0.73–1.50), p = 0.818	1.04 (0.72–1.50), p = 0.853	1.14 (0.81–1.60), p = 0.447	1.14 (0.82–1.59), p = 0.443
**Household decision-making and division of childcare and household tasks**										
Male involvement (household & childcare tasks)	135/1,463 (9.2)	247/1,270 (19.5)	143/1,236 (11.6)	222/1,246 (17.8)	2.44 (1.76–3.37), p = 0.000	2.52 (1.78–3.55), p < 0.001	1.30 (0.95–1.78), p = 0.104	1.29 (0.93–1.80), p = 0.137	2.18 (1.59–3.00), p < 0.001	2.37 (1.69–3.33), p < 0.001
Male dominance in decision-making (food & clothing)	743/1,456 (51.0)	568/1,268 (44.8)	594/1,232 (48.2)	552/1,244 (44.4)	0.77 (0.64–0.92), p = 0.005	0.73 (0.59–0.91), p = 0.006	0.89 (0.76–1.04), p = 0.152	0.92 (0.76–1.12), p = 0.404	0.76 (0.64–0.91), p = 0.002	0.70 (0.57–0.87), p = 0.001
Male dominance in decision-making (large item purchases)	951/1,455 (65.4)	686/1,268 (54.1)	727/1,226 (59.3)	676/1,241 (54.5)	0.62 (0.49–0.79), p = 0.000	0.61 (0.48–0.77), p = 0.000	0.77 (0.61–0.97), p = 0.028	0.76 (0.60–0.97), p = 0.027	0.63 (0.51–0.78), p < 0.001	0.61 (0.49–0.75), p < 0.001
Male dominance in decision-making (spending time with family/friends)	875/1,455 (60.1)	618/1,264 (48.9)	662/1,226 (54.0)	584/1,242 (47.0)	0.62 (0.46–0.83), p = 0.001	0.60 (0.45–0.81), p = 0.001	0.77 (0.58–1.04), p = 0.088	0.76 (0.57–1.02), p = 0.069	0.58 (0.46–0.75), p < 0.001	0.57 (0.45–0.72), p < 0.001
**HIV knowledge, attitudes, behaviors**†										
Confidence in ability to use a condom	291/701 (41.5)	408/631 (64.7)	287/640 (44.8)	330/620 (53.2)	2.65 (1.95–3.62), p < 0.001 [1.57 (1.40–1.73)]^‡^	2.76 (1.98–3.84), p < 0.001 [1.60 (1.41–1.76)]^‡^	1.14 (0.85–1.53), p = 0.391	1.13 (0.83–1.53), p = 0.443	1.61 (1.17–2.22), p = 0.004	1.74 (1.26–2.41), p = 0.001
Been tested for HIV	539/700 (77.0)	524/630 (83.2)	518/640 (80.9)	496/620 (80.0)	1.49 (1.02–2.16), p = 0.039	1.48 (1.04–2.11), p = 0.028	1.27 (0.90–1.78), p = 0.169	1.27 (0.91–1.78), p = 0.158	1.18 (0.81–1.73), p = 0.385	1.27 (0.88–1.82), p = 0.197
Discussed HIV risk with partner	479/701 (68.3)	519/631 (82.3)	487/640 (76.1)	475/620 (76.6)	2.14 (1.50–3.06), p < 0.001	2.14 (1.50–3.07), p < 0.001	1.47 (1.04–2.07), p = 0.027	1.46 (1.03–2.07), p = 0.031	1.50 (1.09–2.05), p = 0.012	1.65 (1.23–2.23), p = 0.001
Discussed sex with partner	513/701 (73.2)	531/631 (84.2)	500/640 (78.1)	512/620 (82.6)	1.96 (1.45–2.65), p < 0.001	1.92 (1.42–2.60), p < 0.001	1.32 (0.98–1.76), p = 0.067	1.28 (0.95–1.72), p = 0.102	1.75 (1.25–2.45), p = 0.001	1.90 (1.33–2.72), p < 0.001

**Abbreviations:** AOR, adjusted OR; IPV, intimate partner violence; ITT, intention to treat; OR, odds ratio; UBL, Unite for a Better Life.

*Adjusted for respondent’s age, respondent’s schooling category, marriage length, polygamous household, socioeconomic status, whether completed the full or short survey at endline, and number of months between end of intervention and endline interview.

^†^Not assessed among spouses of baseline respondents in short endline questionnaire.

^‡^ORs adjusted to approximate the relative risk as per methodology of Zhang and Khai [28] when the outcome of interest is common (>10% in control group) and OR > 2.5 or OR < 0.5.

Assessing the additional IPV outcomes not prespecified in the clinical trial registry, there is evidence of a statistically significant reduction in the composite indicators of IPV, including in women’s experience of past-year physical and/or sexual IPV (AOR = 0.81, 95% CI: 0.66–0.99, p = 0.036) in the men’s UBL intervention arm and in men’s perpetration of past-year physical and/or sexual IPV (AOR = 0.78; 95% CI: 0.62–0.98, p = 0.037) in the men’s UBL intervention arm. There was no significant association between exposure to UBL and these variables in any of the other arms. There was also no significant association between exposure to UBL and experience of or perpetration of emotional IPV across any of the arms.

For additional outcomes as reported by women (Table 5), there was a statistically significant increase in knowledge on IPV-related laws in the men’s UBL arm and in support for equitable norms in the women’s UBL arm. Women also reported a statistically significant increase in male involvement in childcare and household tasks in the couples’ UBL arm, but no changes in male dominance in household decision-making. Finally, there were significant increases in HIV testing among women in the men’s UBL arm and in discussing sex with their partner in all 3 intervention arms.

The UBL intervention was also associated with changes in knowledge, attitudes, decision-making and task-sharing outcomes among men (Table 6). This includes improved IPV knowledge in the male UBL arm and increased support for gender-equitable norms in both the couples’ and the men’s UBL arms. In addition, there were statistically significant changes in all male involvement and household decision-making outcomes as reported by men in the couples’ and men’s UBL arms. There were also significant improvements in HIV testing (men in the couples’ UBL arm) and in discussing sex with their partner (men in the couples’ and men’s UBL arm).

Table 7 presents the effect of the UBL interventions on IPV outcomes at the household level when men’s reports of IPV perpetration and women’s reports of IPV experience in each household were combined. In this post hoc analysis, there was a statistically significant reduction in past-year sexual IPV (AOR = 0.79, 95% CI: 0.64–0.98, p = 0.032) in the men’s UBL arm but no reduction in the couples’ (AOR = 0.88, 95% CI: 0.64–1.19, p = 0.392) or women’s (AOR = 1.11, 95% CI: 0.88–1.42, p = 0.379) UBL arms. There was no decline in past-year physical IPV at the household level in any of the intervention arms (couples UBL arm AOR = 1.02, 95% CI: 0.82–1.27, p = 0.833; women’s UBL arm AOR = 1.18, 95% CI: 0.94–1.49, p = 0.146; men’s UBL arm AOR = 1.01, 95% CI: 0.84–1.21, p = 0.946). For the composite indicator, there is a decline in past-year physical and/or sexual IPV in the men’s UBL arm that is marginally significant at the 10% level (AOR = 0.81, 95% CI: 0.66–1.01, p = 0.059) but no reduction in the couples (AOR = 0.90, 95% CI: 0.67–1.20, p = 0.465) or women’s (AOR = 1.13, 95% CI: 0.86–1.48, p = 0.398) intervention arms.

**Table 7:** Effect of the UBL intervention on IPV outcomes at the household level at 24-month follow-up: ITT analysis.

	Summary Statistics				Intervention Effect					
	Control Group	Couples' UBL	Women's UBL	Men's UBL	Couples' UBL		Women’s UBL		Men's UBL	
	N (%)	N (%)	N (%)	N (%)	OR	AOR*	OR	AOR*	OR	AOR*
Household-level IPV—Combined women’s and men’s reports										
Past-year physical IPV	523/1,508 (34.7)	454/1,300 (34.9)	481/1,270 (37.9)	454/1,276 (35.6)	1.03 (0.84–1.27), p = 0.754	1.02 (0.82–1.27), p = 0.833	1.20 (0.95–1.50), p = 0.119	1.18 (0.94–1.49), p = 0.146	1.05 (0.89–1.25), p = 0.541	1.01 (0.84–1.21), p = 0.946
Past-year sexual IPV	786/1,508 (52.1)	638/1,300 (49.1)	689/1,270 (54.3)	621/1,276 (48.7)	0.89 (0.68–1.17), p = 0.402	0.88 (0.64–1.19), p = 0.392	1.13 (0.89–1.42), p = 0.316	1.11 (0.88–1.42), p = 0.379	0.85 (0.70–1.05), p = 0.131	0.79 (0.64–0.98), p = 0.032
Past-year physical and/or sexual IPV	922/1,508 (61.1)	762/1,300 (58.6)	800/1,270 (63.0)	740/1,276 (58.0)	0.92 (0.70–1.20), p = 0.525	0.90 (0.67–1.20), p = 0.465	1.13 (0.86–1.47), p = 0.380	1.13 (0.86–1.48), p = 0.398	0.87 (0.70–1.07), p = 0.190	0.81 (0.66–1.01), p = 0.059
Past-year emotional IPV	1,188/1,511 (78.6)	1,001/1,303 (76.8)	993/1,274 (77.9)	992/1,277 (77.7)	0.90 (0.62–1.30), p = 0.581	0.91 (0.63–1.34), p = 0.645	0.98 (0.67–1.43), p = 0.898	0.99 (0.66–1.48), p = 0.965	0.95 (0.72–1.26), p = 0.737	0.92 (0.69–1.22), p = 0.562

**Abbreviations:** AOR, adjusted OR; IPV, intimate partner violence; ITT, intention to treat; OR, odds ratio; UBL, Unite for a Better Life.

*Adjusted for respondent’s age, respondent’s schooling category, marriage length, polygamous household, socioeconomic status, whether completed the full or short survey at endline, and number of months between end of intervention and endline interview.

The results of the sensitivity analysis in which the main adjusted models (as presented in Table 4) are compared with adjusted models that include the baseline IPV outcome values are presented in S3 Table. There was no notable difference between the primary adjusted model and the sensitivity analysis.

Finally, a post hoc analysis of highly adherent respondents was conducted. Overall, 72% of intervention participants completed at least 85% of intervention sessions and are classified as highly adherent. This includes 72% in the couples’ UBL arm (including only couples for which both spouses completed 85% of sessions), 85% in the women’s UBL arm, and 62% in the men’s UBL arm. Analysis of outcomes among the highly adherent sample (Table 8) yields relatively larger reductions in IPV outcomes in the men’s UBL arm among men, women, and at the household level, though it should be noted that since the adherence measure is not reported in the control arm, this analysis has the potential for bias. Among highly adherent respondents, the men’s UBL intervention is associated with an almost 50% reduction in the odds of perpetrating past-year sexual IPV and a 42% reduction in the odds of past-year perpetration of physical and/or sexual IPV. A statistically significant reduction in perpetration of past-year physical IPV in the men’s UBL arm was also observed (AOR = 0.71, CI = 0.51–0.98, p = 0.039). Reductions in IPV were also higher at the household level and among the women partnered with highly adherent men in the men’s UBL arm.

**Table 8:** Effect of the UBL intervention on IPV outcomes among women, men, and at the household level at 24-month follow-up: Analysis among those who participated in at least 85% of UBL sessions.

	Summary Statistics				Intervention Effect					
	Control Group	Couples' UBL	Women's UBL	Men's UBL	Couples' UBL		Women’s UBL		Men's UBL	
	N (%)	N (%)	N (%)	N (%)	OR	AOR*	OR	AOR*	OR	AOR*
**Experience of IPV—Women’s reports**										
Past-year physical IPV	292/1,452 (20.1)	124/598 (20.7)	158/688 (23.0)	147/655 (22.4)	1.03 (0.77–1.38), p = 0.835	1.04 (0.76–1.41), p = 0.810	1.18 (0.88–1.57), p = 0.275	1.17 (0.86–1.58), p = 0.329	1.13 (0.90–1.41), p = 0.287	1.04 (0.82–1.33), p = 0.727
Past-year sexual IPV	542/1,451 (37.4)	216/597 (36.2)	279/688 (40.6)	226/653 (34.6)	0.92 (0.65–1.31), p = 0.658	0.94 (0.64–1.38), p = 0.755	1.10 (0.86–1.40), p = 0.444	1.08 (0.81–1.43), p = 0.604	0.83 (0.64–1.07), p = 0.148	0.76 (0.58–1.00), p = 0.054
Past-year physical and/or sexual IPV	627/1,453 (43.2)	248/598 (41.5)	311/688 (45.2)	264/654 (40.4)	0.91 (0.67–1.25), p = 0.560	0.92 (0.66–1.29), p = 0.638	1.05 (0.81–1.35), p = 0.713	1.03 (0.78–1.37), p = 0.825	0.84 (0.67–1.05), p = 0.135	0.78 (0.62–0.99), p = 0.044
Past-year emotional IPV	886/1,460 (60.7)	362/600 (60.3)	423/691 (61.2)	389/656 (59.3)	0.96 (0.68–1.35), p = 0.815	0.98 (0.67–1.45), p = 0.936	0.97 (0.73–1.31), p = 0.865	0.99 (0.71–1.37), p = 0.936	0.89 (0.66–1.20), p = 0.442	0.82 (0.60–1.12), p = 0.218
**Perpetration of IPV—Men’s reports**										
Past-year physical IPV	313/1,459 (21.5)	120/599 (20.3)	156/663 (23.5)	121/689 (17.6)	0.88 (0.59–1.30), p = 0.529	0.88 (0.59–1.30), p = 0.517	1.11 (0.79–1.56), p = 0.531	1.08 (0.77–1.51), p = 0.645	0.73 (0.52–1.01), p = 0.059	0.71 (0.51–0.98), p = 0.039
Past-year sexual IPV	427/1,459 (29.3)	142/599 (23.7)	198/662 (29.9)	135/689 (19.6)	0.72 (0.46–1.13), p = 0.152	0.73 (0.45–1.16), p = 0.179	0.98 (0.78–1.24), p = 0.884	0.97 (0.76–1.23), p = 0.784	0.53 (0.40–0.72), p < 0.001	0.52 (0.39–0.69), p < 0.001
Past-year physical and/or sexual IPV	566/1,459 (38.8)	194/599 (32.4)	275/662 (41.5)	204/689 (29.6)	0.72 (0.49–1.06), p = 0.092	0.72 (0.49–1.07), p = 0.106	1.09 (0.87–1.35), p = 0.457	1.07 (0.85–1.34), p = 0.576	0.60 (0.46–0.79), p < 0.001	0.58 (0.44–0.76), p < 0.001
Past-year emotional IPV	819/1,463 (56.0)	318/599 (53.1)	398/663 (60.0)	383/689 (55.6)	0.87 (0.61–1.24), p = 0.426	0.86 (0.60–1.22), p = 0.401	1.15 (0.91–1.45), p = 0.238	1.12 (0.89–1.42), p = 0.342	0.92 (0.70–1.20), p = 0.522	0.90 (0.69–1.17), p = 0.433
**Household-level IPV—Combined women’s and men’s reports**										
Past-year physical IPV	523/1,508 (34.7)	211/600 (35.2)	268/691 (38.8)	237/678 (35.0)	1.00 (0.99–1.28), p = 0.988	1.00 (0.77–1.28), p = 0.973	1.18 (0.93–1.50), p = 0.180	1.16 (0.91–1.48), p = 0.218	0.99 (0.80–1.24), p = 0.956	0.94 (0.75–1.19), p = 0.627
Past-year sexual IPV	786/1,508 (52.1)	306/600 (51.0)	383/691 (55.4)	314/678 (46.3)	0.91 (0.65–1.28), p = 0.586	0.91 (0.64–1.31), p = 0.615	1.07 (0.85–1.35), p = 0.557	1.05 (0.82–1.34), p = 0.710	0.72 (0.57–0.92), p = 0.007	0.68 (0.54–0.86), p = 0.001
Past-year physical and/or sexual IPV	922/1,508 (61.1)	358/600 (59.7)	443/691 (64.1)	382/678 (56.3)	0.89 (0.65–1.22), p = 0.474	0.89 (0.64–1.23), p = 0.474	1.10 (0.83–1.38), p = 0.602	1.06 (0.80–1.39), p = 0.690	0.76 (0.59–0.97), p = 0.030	0.71 (0.55–0.91), p = 0.008
Past-year emotional IPV	1,188/1,511 (78.6)	467/600 (77.8)	556/691 (80.5)	547/678 (80.7)	0.88 (0.59–1.32), p = 0.545	0.90 (0.59–1.36), p = 0.617	1.02 (0.71–1.45), p = 0.931	1.03 (0.70–1.53), p = 0.871	1.08 (0.75–1.55), p = 0.674	1.04 (0.72–1.51), p = 0.832

**Abbreviations:** AOR, adjusted OR; IPV, intimate partner violence; OR, odds ratio; UBL, Unite for a Better Life.

*Adjusted for respondent’s age, respondent’s schooling category, marriage length, polygamous household, socioeconomic status, whether completed the full or short survey at endline, and number of months between end of intervention and endline interview

## Discussion

The UBL intervention did not have significant effects on women’s reported experience of physical IPV or sexual IPV at approximately 24-month follow-up. However, when delivered to men, the intervention significantly reduced men’s reported perpetration of sexual IPV. In addition, the UBL intervention when delivered to men was associated with a significant reduction in the composite IPV indicators including women’s experience of past-year physical and/or sexual IPV as well as male perpetration of past-year physical and/or sexual IPV. However, there was no impact on IPV when UBL was delivered to couples or to women. In addition, there was no evidence of any reduction in experience or perpetration of emotional IPV alone in any of the intervention arms. These results are consistent across men’s, women’s, and combined household reports, suggesting the findings are robust and do not merely reflect social desirability bias in reporting. In addition, the UBL intervention was associated with a significant improvement in comprehensive HIV knowledge and a reduction in HIV risk behaviors, as well as more equitable intrahousehold decision-making and task-sharing, but these effects varied based on UBL intervention arm and participant sex.

The findings are broadly consistent with other IPV prevention trials [19,29,30] that have demonstrated positive impacts of individual-level gender-transformative programming in reducing IPV. However, our results are notable in several ways. First, the significant reductions in IPV in this trial were driven primarily by reductions in sexual IPV. A reduction in physical violence is observed only among the highly adherent sample in the men’s arm and not in the ITT analysis, unlike in several other trials [14,19,31]. Both the 10-session MAISHA program and the SASA! community-level intervention trials reported the opposite pattern, with reductions in physical IPV but limited effects on sexual IPV [12,32]. Researchers have hypothesized that attitudes and behaviors regarding sexual IPV may be harder to shift than those related to physical IPV [12]. The reductions in sexual IPV among UBL participants were observed in conjunction with increased knowledge of IPV laws and improved couples’ communications, including specifically discussions on sexuality, and are consistent with the fact that the UBL curriculum included substantial content on healthy sexuality, sexual relationships, consent, and pleasure. In addition, they may represent an increase in women’s negotiating power in sexual relationships, consistent with the observed increases in reported condom use.

The absence of an effect of the UBL intervention on physical IPV may be surprising, but it should be noted that there was also no effect of the intervention on men’s and women’s attitudes justifying or accepting the use of physical IPV. Some research has reported reductions in violence and behavior change without corresponding changes in attitudes and beliefs [21]. However, quantitative and qualitative research from the SASA! trial suggests that the pathway through which that intervention led to reductions in physical IPV was through changes in norms around acceptability of IPV and improved communication within relationships [33,34]. The UBL intervention was successful in significantly improving couples’ communication and support for gender-equitable norms among men and women but did not generate any shift in the acceptability of physical violence. This may help to explain the findings on physical IPV; however, a significant reduction in perpetration of physical IPV was associated with the men’s UBL intervention in the highly adherent sample that completed at least 12 of the 14 intervention sessions. This suggests that a higher exposure to the UBL intervention may be needed in order to achieve reductions in physical IPV in this particular setting.

The lack of intervention effect on emotional IPV is similar to the MAISHA and the Safe Homes and Respect for Everyone (SHARE) intervention trials, both of which reported no effect on emotional abuse [14, 32]. In the UBL trial, emotional abuse was highly prevalent, with approximately 60% of women reporting past-year emotional IPV. Emotional abuse is linked with poor mental health outcomes that are distinct from physical or sexual IPV [35], and further research is needed to understand how to address this important but neglected form of IPV [36].

Our findings demonstrate that IPV outcomes were only impacted by UBL when men alone received the intervention; neither the couples’ nor the women’s UBL led to reductions in any IPV outcomes. While couples’ interventions have been identified as promising in existing literature [20,21], limited rigorous testing of such interventions has been conducted. A pilot trial of a couples’ intervention delivered to women and their male partners in Côte d’Ivoire found no significant impact on IPV outcomes [29]. A different couples’ intervention, the Bandebereho program, generated large and significant reductions in experience of physical and sexual IPV among women when evaluated in an RCT in Rwanda [19]. However, this intervention did not involve couples participating together in all sessions as in the UBL couples’ program. Rather, couples participated together in 8 of 15 sessions, and the remaining 7 sessions included only male participants. In terms of direct beneficiary exposure to content, Bandebereho is therefore intermediate between the couples’ and men’s UBL programs. One other trial of a couples’ intervention in India that reported a reduction in sexual IPV delivered 2 of the 3 sessions to men alone [37].

Given this broader evidence base, our findings suggest that it is critical for IPV interventions to allow sufficient time for men to engage with their peers in same-sex groups, especially for sensitive topics, in order to enable reflection, challenge norms, and support and reinforce the desired outcomes. While the couples’ UBL intervention did incorporate some separate same-sex discussions, the frequency and length of these same-sex discussions may not have been sufficient to elicit change. It is also important to note there were some differences in content between the men’s and couples’ UBL programs, including the fifth session (anger management in men’s UBL versus power in relationships in couples’ UBL).

The absence of any significant effects of the women’s UBL intervention on IPV outcomes also differs from some existing evidence. An intervention delivered to women in India reduced sexual coercion, though not IPV [38]. A 10-session IPV prevention intervention delivered to women in conjunction with microfinance in South Africa, evaluated in the Intervention With Microfinance for AIDS and Gender Equity (IMAGE) trial, also reduced experience of past-year physical and/or sexual IPV among women by 55% at 24-month follow-up [25]. UBL did not have an economic empowerment component, and this could have influenced its potential for effectiveness among women; however, evidence suggests that the positive impacts of the IMAGE trial were driven primarily by the gender/HIV component [39]. Nevertheless, the relationship between economic factors and IPV risk is complex and appears to be context-specific. While not statistically significant, there were increased coefficients for several of the IPV outcomes in the women’s UBL arm that warrant further discussion. This may have been due to changes in reporting patterns as women gained awareness of IPV, but the pattern is visible among the male spouses in this arm who were not exposed to the intervention. The potential negative effects of the women’s UBL program require further assessment, possibly through qualitative interviews.

Importantly, the UBL intervention was also associated with a significant change in a variety of HIV outcomes in both the couples’ and the men’s arms, including the prespecified secondary outcomes (comprehensive HIV knowledge and condom use at last intercourse) and additional secondary outcomes (HIV testing and discussing sex with their partner). It is not clear whether these behavioral changes resulted in reduced HIV transmission because this evaluation did not have sufficient power to assess effects on HIV incidence given current prevalence rates in Ethiopia [22]. The increase in communication and knowledge around sexual behavior and HIV is consistent with the reduction in violence observed primarily for sexual violence and suggests that the intervention may have been particularly effective at shifting norms around couples’ sexual relationships. UBL roleplays and skills development on sexual consent and setting boundaries potentially contributed to these effects. Ethiopia has a higher prevalence of sexual IPV than other settings [10], and it is unclear whether UBL may lead to reductions in sexual violence in lower-prevalence contexts.

The UBL intervention also contributed to improvements in several other additional outcomes, including men’s reported involvement in household tasks and childcare and dominance in decision-making in the couples’ and men’s arms. These are important findings given that male dominance in decision-making is associated with poor health outcomes for women and children and contributes to inequitable relationship dynamics, which is an important driver of IPV [40]. The increased participation of men in domestic tasks suggests some softening of traditional gender roles around division of labor and, together with the increase in joint decision-making and communication, is indicative of positive changes in relationships. These findings confirm that the intervention was successful in altering the unequal gendered power dynamics underlying partner violence and creating an environment in which violence is less likely.

It should be noted that our analysis of trial findings entails comparison of multiple outcomes (the prespecified outcomes of interest include 2 primary outcomes and 4 secondary outcomes), each analyzed in 3 intervention arms. Accordingly, the results should be interpreted cautiously in light of challenges around multiple hypothesis testing [41,42]; the CONSORT guidelines for reporting randomized controlled trials note that “Authors should exercise special care when evaluating the results of trials with multiple comparisons” [41]. In interpretation, it is important to emphasize the broad pattern of consistency across related outcomes. For example, generally comparable results are observed for experience and perpetration of sexual IPV and experience and perpetration of physical IPV. There is also a broad pattern of consistency in the results for different arms with the men’s UBL intervention uniformly observed to be the most effective arm in reducing IPV. Accordingly, we feel confident in interpreting these patterns as credible evidence of statistically significant experimental effects, even given the evaluation of multiple coefficient estimates.

This trial has a number of strengths and weaknesses. To our knowledge, this is the first RCT to systematically compare the relative effectiveness of delivering a gender-transformative IPV prevention intervention to women, men, and couples. The trial included data from both men and women, collected by separately interviewing both spouses within the sampled households. This is a novel strategy in IPV research that allows evaluation of the consistency of changes in outcomes as reported by both members of the couple. Equally important, given that only 1 spouse participated in the intervention in 2 of the trial arms, data from the nonparticipating spouse are presumably not affected by reporting biases linked to intervention exposure. This again allows assessment of the consistency of reported effects of the intervention.

Other strengths of the trial include the collection of data on a range of different outcomes and the inclusion of a large sample size, a standardized questionnaire, and a well-trained team to minimize potential measurement error. There was a high level of participation in the intervention, which was confirmed through independent observational attendance records. Households were randomly selected for participation in the intervention, enabling a representative sample population, and loss to follow-up was minimal.

This trial also has several limitations. First, since spouses of baseline respondents were only surveyed at endline, their baseline data are not available. However, information about the spouses’ background and other household characteristics was reported by baseline respondents, enabling the inclusion of baseline controls for the entire endline sample. Second, we were unable to mask intervention assignment from participants or from endline enumerators. Third, the trial includes 2 primary outcomes and 4 secondary outcomes, and the analysis of multiple comparisons should accordingly be interpreted with caution in line with existing guidance [41,42].

Fourth, as with other behavioral trials, our outcomes are self-reported and may be subject to recall and social desirability bias. Under-reporting of IPV is common, and it is possible that exposure to the intervention could have led to increased or decreased reporting of IPV among women and men in UBL arms. This would have resulted in either a lower or higher estimated intervention effect than the true impact. Some studies have suggested that men are more prone to social desirability bias related to IPV than women and that these differential biases may be further exacerbated by intervention exposure [12,14,25]. However, in this trial, endline IPV prevalence in each arm is similar among men and women’s reports (though sexual IPV is slightly higher via women’s reports), indicating minimal differential reporting.

If the observed reductions in IPV reflect primarily social desirability bias and differential reporting rather than true intervention effects, we would expect to observe reported reductions among participants directly exposed to the intervention, but not among their unexposed spouses, in all treatment arms. However, we see no reported reductions in IPV in the couples’ or women’s arms. In addition, if reporting biases were substantial, we would expect a larger reduction in violence to be observed in the reports of the male spouses in the men’s arm relative to the reports of the unexposed female spouses. However, in fact, we observe that the effect sizes for IPV outcomes in the men’s UBL arm are of similar magnitude for men’s reports, women’s reports, and combined household reports.

Further evidence that the observed IPV reductions are true intervention effects can be found from the consistency between the ITT analysis and the analysis of the highly adherent sample. As would be expected, there are stronger effects among highly adherent participants/households within the men’s UBL arm, providing further evidence of the robustness of the results. Together, the consistency, directionality, and statistical significance of findings across a broad range of outcomes provide compelling evidence of the effectiveness of the UBL intervention.

In summary, this trial demonstrates the effectiveness of a 14-session in-person gender-transformative intervention delivered to groups of men within the context of the traditional coffee ceremony in reducing perpetration of IPV in a rural Ethiopian setting, with additional evidence supporting relationships between the intervention and men’s perpetration and women’s experience of sexual and/or physical IPV. Our study makes unique contributions to the existing evidence base around IPV prevention and highlights the relative effectiveness of working with men compared to couples or women in this context. Further research is needed to understand why couples’ and women’s UBL interventions were not effective in reducing IPV in this context and the potential mechanisms through which the men’s UBL program led to change. Further research should also focus on understanding the optimal number of sessions needed to elicit positive outcomes and the role of same-sex versus mixed-sex discussions within couples’ programming. Beyond IPV, the UBL intervention was associated with positive effects on a range of other outcomes, including HIV risk behaviors, intrahousehold decision-making, and male involvement in household tasks. The intervention demonstrates promise as a strategy that could be replicated and tested in other settings and that could help accelerate progress towards achieving gender equality and combating HIV/AIDS.

## Supporting information

S1 Table CONSORT checklist. CONSORT, Consolidated Standards of Reporting Trials.
(DOCX)

S2 Table TIDieR checklist. TIDieR, Template for Intervention Description and Replication.
(DOCX)

S3 Table Results of sensitivity analysis. (DOCX)

S1 Text Trial protocol. (DOC)

S2 Text Preanalysis plan. (PDF)

S3 Text Baseline women’s questionnaire. (PDF)

S4 Text Baseline men’s questionnaire. (PDF)

S5 Text Endline women’s questionnaire. (PDF)

S6 Text Endline men’s questionnaire. (PDF)

## Abbreviations

AAU: Addis Ababa University
AEA: American Economic Association
AOR: adjusted OR
CI: confidence interval
CONSORT: Consolidated Standards of Reporting Trials
COUHES: Committee on the Use of Humans as Experimental Subjects
cRCT: cluster-randomized controlled trial
DHS: Demographic and Health Survey
EPHA: Ethiopian Public Health Association
HAPCO: HIV/AIDS Prevention and Control Office
HEW: health extension worker
IMAGE: Intervention With Microfinance for AIDS and Gender Equity
IPV: intimate partner violence
ITT: intention to treat
J-PAL: Abdul Latif Jameel Poverty Action Lab
MIT: Massachusetts Institute of Technology
OR: odds ratio
RCT: randomized controlled trial
SHARE: Safe Homes and Respect for Everyone
SNNPR: Southern Nations, Nationalities and People’s Region
STI: sexually transmitted infection
TIDieR: Template for Intervention Description and Replication
UBL: Unite for a Better Life
