# Supplementary material for: Effectiveness of a culturally appropriate intervention to prevent intimate partner violence and HIV transmission among men, women, and couples in rural Ethiopia: Findings from a cluster-randomized controlled trial
Source: PLoS Med. 2020 Aug 18;17(8):e1003274. doi: 10.1371/journal.pmed.1003274 (PMC7433859; doi:10.1371/journal.pmed.1003274)
Supplement: S3 Table — (DOCX) [file pmed.1003274.s003.docx]

Table S3: Effect of the UBL intervention on IPV outcomes among women and men at 24–months follow–up; ITT analysis comparing models without and with adjustment for baseline IPV measures.

|  | **Summary Statistics** | | | | **Intervention Effect** | | | | | |
| --- | --- | --- | --- | --- | --- | --- | --- | --- | --- | --- |
|  | **Control Group** | **Couples' UBL** | **Women's UBL** | **Men's**  **UBL** | **Couples' UBL** | | **Women’s UBL** | | **Men's UBL** | |
|  | N (%) | N (%) | N (%) | N (%) | AOR^*^ | AOR^**^ | AOR^*^ | AOR^**^ | AOR^*^ | AOR^**^ |
| **PRIMARY IPV OUTCOMES** | | | | | | | | | | |
| **Experience of IPV—Women’s Reports** | | | | | | | | | | |
| Past-year physical IPV | 292/1,452  (20.1) | 255/1,249  (20.4) | 267/1,211  (22.1) | 268/1,233  (21.7) | 1.00 (0.77–1.30), p=0.973 | 1.02 (0.79–1.33), p=0.860 | 1.11 (0.87–1.42), p=0.414 | 1.12 (0.88–1.43), p=0.369 | 1.02 (0.81–1.28), p=0.865 | 1.03 (0.82–1.29), p=0.795 |
| Past-year sexual IPV | 542/1,451  (37.4) | 424/1,248  (34.0) | 494/1,212  (40.8) | 430/1,228  (35.0) | 0.86 (0.62–1.20), p=0.378 | 0.87 (0.63–1.20), p=0.390 | 1.15 (0.89–1.50), p=0.291 | 1.15 (0.88–1.50), p=0.302 | 0.80 (0.63–1.01), p=0.062 | 0.80 (0.63–1.01), p=0.063 |
| **SECONDARY IPV OUTCOMES** | | | | | | | | | | |
| **Perpetration of IPV—Men’s Reports** | | | | | | | | | | |
| Past-year physical IPV | 313/1,459  (21.5) | 272/1,268  (21.5) | 309/1,230  (25.1) | 242/1,244  (19.5) | 0.97 (0.70–1.35), p=0.866 | 0.99 (0.71–1.37), p=0.937 | 1.21 (0.88–1.67), p=0.232 | 1.22 (0.90–1.66), p=0.219 | 0.85 (0.65–1.09), p=0.200 | 0.86 (0.66–1.11), p=0.238 |
| Past-year sexual IPV | 427/1,459  (29.3) | 347/1,268  (27.4) | 382/1,229  (31.1) | 303/1,244  (24.4) | 0.87 (0.61–1.25), p=0.462 | 0.88 (0.61–1.25), p=0.467 | 1.07 (0.84–1.37), p=0.570 | 1.07 (0.84–1.36), p=0.586 | 0.73 (0.56–0.94), p=0.014 | 0.73 (0.57–0.93), p=0.013 |
| **ADDITIONAL IPV OUTCOMES** | | | | | | | | | | |
| **Perpetration of IPV—Women’s Reports** | | | | | | | | | | |
| Past-year physical and/or sexual IPV | 627/1,435  (43.2) | 496/1,249  (39.7) | 549/1,211  (45.3) | 497/1,230  (40.4) | 0.87 (0.65–1.15), p=0.326 | 0.88 (0.67–1.18), p=0.364 | 1.09 (0.84–1.40), p=0.525 | 1.09 (0.84–1.40), p=0.515 | 0.81 (0.66–0.99), p=0.036 | 0.81 (0.66–0.99), p=0.035 |
| Past-year emotional IPV | 886/1,460  (60.7) | 741/1,253  (59.1) | 712/1,217  (58.5) | 717/1,236  (58.0) | 0.92 (0.65–1.31), p=0.655 | 0.93 (0.66–1.32), p=0.694 | 0.90 (0.66–1.23), p=0.499 | 0.89 (0.65–1.22), p=0.470 | 0.81 (0.62–1.05), p=0.114 | 0.82 (0.63–1.06), p=0.132 |
| **Perpetration of IPV—Men’s Reports** | | | | | | | | | | |
| Past-year physical and/or sexual IPV | 566/1,459  (38.8) | 462/1,268  (36.4) | 526/1,229  (42.8) | 430/1,244  (34.6) | 0.87 (0.62–1.21), p=0.406 | 0.88 (0.63–1.23), p=0.453 | 1.17 (0.91–1.50), p=0.212 | 1.18 (0.92–1.50), p=0.190 | 0.78 (0.62–0.98), p=0.037 | 0.79 (0.62–0.99), p=0.048 |
| Past-year emotional IPV | 819/1,463  (56.0) | 711/1,270  (56.0) | 749/1,236  (60.6) | 695/1,246  (55.8) | 0.99 (0.76–1.29), p=0.922 | 0.99 (0.76–1.28), p=0.934 | 1.20 (0.93–1.55), p=0.168 | 1.20 (0.93–1.56), p=0.163 | 0.97 (0.78–1.22), p=0.801 | 0.98 (0.78–1.22), p=0.850 |

**Abbreviations:** AOR, adjusted odds ratio; IPV, intimate partner violence; ITT, intention to treat; UBL, Unite for a Better Life

*Adjusted for respondent’s age, respondent’s schooling category, marriage length, polygamous household, socioeconomic status, whether completed the full or short survey at endline, and number of months between end of intervention and endline interview (as in Table 4)

**Adjusted for respondent’s age, respondent’s schooling category, marriage length, polygamous household, socioeconomic status, whether completed the full or short survey at endline, and number of months between end of intervention and endline interview and baseline experience/perpetration of IPV
