# Supplementary material for: Effectiveness of a culturally appropriate intervention to prevent intimate partner violence and HIV transmission among men, women, and couples in rural Ethiopia: Findings from a cluster-randomized controlled trial
Source: PLoS Med. 2020 Aug 18;17(8):e1003274. doi: 10.1371/journal.pmed.1003274 (PMC7433859; doi:10.1371/journal.pmed.1003274)
Supplement: S2 Text — (PDF) [file pmed.1003274.s005.pdf]

Statistical Analysis Plan: Unite for a Better Life Trial

Version Date: May 2018

A. Overview

The objective of this analysis plan is to summarize the planned analysis for the project "Using cultural ceremonies to reduce intimate partner violence and HIV transmission." This analysis plan represents a full overview of all the planned analysis; this may be published in multiple papers.

B. Sample

The sample will include the full sample of women surveyed (including both baseline respondents and the spouses of baseline male respondents) and men surveyed (including both baseline respondents and the spouses of baseline female respondents.) Baseline information at the individual level will not be available for spouses who were not previously surveyed. As a robustness check, we may also explore specifications estimated using the restricted sample of individuals surveyed at baseline, and adding baseline control variables.

C. Defining primary outcomes of interest

We can define primary categories of outcomes for the study as follows. In addition to reporting results for each individual outcome, we will report average standardized effects for each category of outcome.

**Intervention exposure**	
Attendance at sessions	Women's Long Survey<br>Women's Short Survey<br>Men's Long Survey<br>Men's Short Survey
Reports remembering session content	Women's Long Survey<br>Women's Short Survey<br>Men's Long Survey<br>Men's Short Survey
Reports sharing information obtained with others	Women's Long Survey<br>Women's Short Survey<br>Men's Long Survey<br>Men's Short Survey
Reports continued interactions with group members	Women's Long Survey<br>Women's Short Survey<br>Men's Long Survey<br>Men's Short Survey
**Violence against women: experience / perpetration**	
Any experience of physical IPV<br>(past year)	Women's Long Survey<br>Women's Short Survey

Any experience of sexual IPV<br>(past year)	Women's Long Survey<br>Women's Short Survey
Any experience of physical and/or sexual IPV (past year)	
Any experience of emotional IPV (past year)	Women's Long Survey<br>Women's Short Survey
Any experience of controlling behaviors (past year)	Women's Long Survey
Ever disclosed IPV (since intervention)	Women's Long Survey<br>Women's Short Survey
Any perpetration of emotional violence	Men's Long Survey<br>Men's Short Survey
Any perpetration of physical violence	Men's Long Survey<br>Men's Short Survey
Any perpetration of sexual violence	Men's Long Survey<br>Men's Short Survey
Any perpetration of physical and/or sexual violence	
**Secondary indices related to IPV vulnerability**	
Couples communication	Men's Long Survey
Frequency of marital discord	Women's Long Survey<br>Men's Long Survey
Conflict resolution	Women's Long Survey<br>Men's Long Survey
Knowledge about laws relative to IPV	Women's Long Survey<br>Men's Long Survey
Attitudinal index around gender norms and violence	Women's Long Survey<br>Men's Long Survey<br>Men's Short Survey
**Alternate measures of violence**	
Reports experience of violence via individual quasi-list question	Women's Long Survey<br>Women's Short Survey
Reports perpetration of violence via individual quasi-list question	Men's Long Survey<br>Men's Short Survey

Community-level estimate of violence prevalence constructed using list experiment	Women's Long Survey<br>Women's Short Survey
Community-level estimate of violence perpetration constructed using list experiment	Men's Long Survey<br>Men's Short Survey
Community-level estimate of violence prevalence constructed using anonymous picture method	Women's Long Survey<br>Women's Short Survey
Community-level estimate of violence perpetration constructed using anonymous picture method	Men's Long Survey<br>Men's Short Survey
Community-level estimate of violence prevalence constructed using neighborhood method	Women's Long Survey<br>Men's Long Survey<br>Women's Short Survey<br>Men's Short Survey
**HIV**	
Attitudes toward people living with HIV	Women's Long Survey<br>Men's Long Survey
Exhibits comprehensive knowledge on HIV prevention	Women's Long Survey<br>Men's Long Survey
Number of sexual partners in past year/six months	Women's Long Survey<br>Men's Long Survey
Used a condom at last sex	Women's Long Survey<br>Men's Long Survey
Confidence in convincing the partner to use a condom	Women's Long Survey<br>Men's Long Survey
Confidence in using a condom	Women's Long Survey<br>Men's Long Survey
Ever received voluntary counseling and testing for HIV	Women's Long Survey<br>Men's Long Survey
Ever discussed sexuality (HIV risk and/or sex)	Women's Long Survey<br>Men's Long Survey
**Gender perceptions, social norms, and perceived social norms**	
Perceived rates of IPV in community	Women's Long Survey<br>Women's Short Survey<br>Men's Long Survey<br>Men's Short Survey

Perceptions of others' beliefs and behaviors with respect to female autonomy and acceptance toward IPV	Women's Long Survey<br>Men's Long Survey<br>Men's Short Survey
Attitudes toward IPV specifically and potential justifications	Women's Long Survey<br>Men's Long Survey<br>Men's Short Survey
Perception of violence against women as a major problem in the village	Women's Long Survey<br>Women's Short Survey<br>Men's Long Survey<br>Men's Short Survey
**Intrahousehold decision-making**	
Experience of financial controlling behaviors (past year)	Women's Long Survey<br>Men's Long Survey
Intrahousehold bargaining power of women	Women's Long Survey<br>Women's Short Survey<br>Men's Long Survey<br>Men's Short Survey
Division of household labor	Women's Long Survey<br>Women's Short Survey<br>Men's Long Survey<br>Men's Short Survey
**General wellbeing and psychological status**	
Depression score as measured using the PHQ-9	Women's Long Survey
Effect of depression on daily functioning	Women's Long Survey
**Violence severity**	
Experience of physical injuries due to violence (past year)	Women's Long Survey<br>Women's Short Survey
Any experience of IPV during pregnancy	Women's Long Survey
Report of health care needed to treat consequences of abuse (past year)	Women's Long Survey<br>Women's Short Survey
Experience of severe physical IPV (past year)	Women's Long Survey<br>Women's Short Survey
Experience of severe sexual IPV (past year)	Women's Long Survey<br>Women's Short Survey
Experience of severe emotional IPV (past year)	Women's Long Survey

	Women's Short Survey
Perpetration of severe physical IPV (past year)	Men's Long Survey<br>Men's Short Survey
Perpetration of severe sexual<br>IPV (past year)	Men's Long Survey<br>Men's Short Survey
Perpetration of severe emotional IPV (past year)	Men's Long Survey<br>Men's Short Survey

D. Analysis to be conducted

First, we will report balance tests for baseline characteristics comparing across treatment and control communities, focusing on baseline demographic characteristics as well as baseline values of the outcomes of interest. We will also report a joint test for balance across all characteristics examined.

Second, the primary results will be estimated by regressing the outcomes of interest on dummy variables for the three treatment arms; we will also estimate a joint p-value testing whether there is an effect of any treatment on the outcome of interest. Logistic specifications will be employed for dependent variables that are zero/one indicator variables. All regressions will include district fixed effects, given that randomization was conducted at the level of the district; standard errors will be clustered at the kebele level. Statistical significance will be considered at a p-value of <0.05, while p-values between $0.05<p<0.1$ will be reported as marginally significant in order to balance disciplinary conventions.

Secondary analyses will include analysis examining heterogeneity comparing those individuals within communities who were invited to participate in the intervention and those individuals who were not invited, as well as heterogeneity correlated with reported attendance.
