# Supplementary material for: Effectiveness of a culturally appropriate intervention to prevent intimate partner violence and HIV transmission among men, women, and couples in rural Ethiopia: Findings from a cluster-randomized controlled trial
Source: PLoS Med. 2020 Aug 18;17(8):e1003274. doi: 10.1371/journal.pmed.1003274 (PMC7433859; doi:10.1371/journal.pmed.1003274)
Supplement: S3 Text — (PDF) [file pmed.1003274.s006.pdf]

Women's Health and Relationships Project in Ethiopia.

Collaborative Study by

The School of Public Health, Addis Ababa University;

The Ethiopian Public Health Association

and

The Abdul Latif Jameel Poverty Action Lab.

Women's Questionnaire
የሴቶች መጠይቅ

Addis Ababa, Ethiopia
Nov 2014

Eligibility of study subject

1. Woman in the age between 18-49 years
2. Currently living in marriage or cohabiting with a man
3. Residing in the selected village for at least 6 months

QUESTIONS ABOUT THE HOUSEHOLD

	QUESTIONS & FILTERS	CODING CATEGORIES	
1.	If you don't mind, I would like to ask you a few questions about your household.<br>What is the main source of drinking water for your household?	TAP/PIPED WATER IN RESIDENCE .... 01<br>OUTSIDE TAP (PIPED WATER) .... 02<br>PUBLIC TAP .... 03<br>WELL WATER, WITHIN RESIDENCE .... 04<br>OUTSIDE / PUBLIC WELL .... 05<br>SPRING WATER .... 06<br>RIVER / STREAM / POND / LAKE / DAM .... 08<br>RAINWATER .... 09<br>TANKER / TRUCK / WATER VENDOR .... 10<br>OTHER: ________________ ... 96<br>DON'T KNOW/DON'T REMEMBER .... 98<br>REFUSED/NO ANSWER 99	
2.	What are the main materials used in the roof?<br>RECORD OBSERVATION	ROOF FROM NATURAL MATERIALS .... 1<br>RUDIMENTARY ROOF .... 2<br>TILED OR CONCRETE ROOF .... 3<br>CORRUGATED IRON .... 4<br>OTHER: ________________ ..... 6<br>DON'T KNOW/DON'T REMEMBER .... 8<br>REFUSED/NO ANSWER 9	
3.	Does your household have:<br>a) Electricity<br>b) A radio<br>c) A television<br>d) A telephone<br>e) A refrigerator	YES NO<br>a) ELECTRICITY 1 2<br>b) RADIO 1 2<br>c) TELEVISION 1 2<br>d) TELEPHONE 1 2<br>e) REFRIGERATOR 1 2	DK<br>8<br>8<br>8<br>8<br>8
4.	Do people in your household own any land?	YES .... 1<br>NO .... 2<br>DON'T KNOW/DON'T REMEMBER .... 8<br>REFUSED/NO ANSWER 9	
5.	How many rooms in your household are used for sleeping?	NUMBER OF ROOMS .... [ ][ ]<br>DON'T KNOW/DON'T REMEMBER .... 8<br>REFUSED/NO ANSWER 9	
6.	Are you member of a social banking system called "Ekub"	YES .... 1<br>NO 2	
7.	Are you member of a social support system called "edir"	YES .... 1<br>NO 2	
8	If you got something that made you happy, who would come to participate your happiness	Most of Kebele members .... 1<br>Most of your neighbors .... 2<br>Some of your neighbors .... 3<br>No one will participate 4	
9	If you sustained a heavy problem (eg death of a family member), who would participate to support you	Most of Kebele members .... 1<br>Most of your neighbors .... 2<br>Some of your neighbors .... 3<br>No one will participate 4	
10	In the last week, lack of money, is there a person in a family who sustained hunger per a day?	YES .... 1<br>NO 2	
11	Based on your perception, how do you rank your economically with your neighbors	Higher .... 1<br>Moderate .... 2<br>Lower 3	
12	Based on your perception, how do you rank your social status (being heard) with your neighbors	Higher .... 1<br>Moderate .... 2<br>Lower 3	

BASELINE SURVEY

Identity Number: |__|__|__| |__|__|__| |__|__|__|
Woreda Gotte House No

Enumerator Information

1.	Names and Codes of enumerators	______________________ |__|__|__|
2.	Name and codes of the supervisors	______________________ |__|__|__|

Identification

3.	District (Woreda)	W01 Meskan<br>W02 Mareko<br>W03 Silte<br>W04 Sodo	|__|__|__|
4.	Village (Kebele), name and code		|__|__|__|
5.	Subvillage (Gote), name and code		|__|__|__|
6.	Name of respondent	|____|____|____| Name Father's Name Grand name	
7.	Name of spouse	|____|____|____| Name Father's Name Grand name	
8.	Address (write down names of road, alley, house number) Given by supervisor (computer identity)	|__|__|__|	
9.	Identity Number	|__|__|__| |__|__|__| |__|__|__| Woreda Gotte House No	
10.	Telephone/Cellphone<br>Self…. Spouse .Family… Neighbor..<br>If no phone number put 000000	|__|__|__|__|__|__|__|__|__|__| 1. Self 2. Spouse 3 Family 4.Neighbor 5. None	

Results of Visits

11.		First Visit	Second Visit	Third Visit
12.	Date	|__|__| / |__|__| / 20|__|__| Date Month Year	|__|__| / |__|__| / 20|__|__| Date Month Year	|__|__| / |__|__| / 20|__|__| Date Month Year
13.	Time Interview Started	|__|__| : |__|__| Hour Minute	|__|__| : |__|__| Hour Minute	|__|__| : |__|__| Hour Minute
14.	Time interview completed	|__|__| : |__|__| Hour Minute	|__|__| : |__|__| Hour Minute	|__|__| : |__|__| Hour Minute
15.	Results of Visits	1. [ ] Completed<br>2. [ ] Partly Completed<br>3. [ ] Respondent not at home<br>4. [ ] Respondent Declined<br>5. [ ] Respondent not available in the village<br>6. [ ] Other (Specify)_____<br>Make appointment if 2 or 3 answered	1. [ ] Completed<br>2. [ ] Partly Completed<br>3. [ ] Respondent not at home<br>4. [ ] Respondent Declined<br>5. [ ] Respondent not available in the village<br>6. [ ] Other (Specify)_____<br>1. Make appointment if 2 or 3 answered	1. [ ] Completed<br>2. [ ] Partly Completed<br>3. [ ] Respondent not at home<br>4. [ ] Respondent Declined<br>5. [ ] Respondent not available in the village<br>6. [ ] Other (Specify)_____

A. Background Info/Education/Community Participation/SES

A1.	What is your relationship to the head of the household?	1. [ ] Head<br>2. [ ] Wife<br>3. [ ] Co-wife
A2.	In what day, month and year were you born?<br><br>If she does not know her birth date – write 01/01/ for date and month, and use year	└─┴─┘ / └─┴─┘ / └─┴─┴─┴─┘<br>Day month Year
A3.	How old were you at your last birthday?	└─┴─┘ Years
A4.	Are you able to read?	1. [ ] Yes<br>2. [ ] No **>>>>>SKIP TO A9**
A5.	Have you ever attended school?	1. [ ] Yes<br>2. [ ] No **>>>SKIP to A8**
A6.	What is the highest level of school you attended- primary, secondary or higher?	1. [ ] Primary (grade 1-8)<br>2. [ ] Secondary (grade 9-12)<br>3. [ ] Higher (>=13)
A7.	What is the highest class/form/year you completed at that level?	└─┴─┘
A8.	How often do you read a newspaper or magazine?	1. [ ] Almost every day<br>2. [ ] At least once a week<br>3. [ ] Less than once a week<br>4. [ ] Not at all
A9.	How often do you listen to the radio? ?	1. [ ] Almost every day<br>2. [ ] At least once a week<br>3. [ ] Less than once a week<br>4. [ ] Not at all
A10.	How often do you watch television?	1. [ ] Almost every day<br>2. [ ] At least once a week<br>3. [ ] Less than once a week<br>4. [ ] Not at all
A11.	What is your religion?	1. [ ] Muslim<br>2. [ ] Orthodox<br>3. [ ] Protestant<br>4. [ ] Catholic<br>5. [ ] Other _______________
A12.	How many years have you lived in this village?	└─┴─┘ years └─┴─┘ Months
A13.	How was your marriage ceremony conducted?	1.[ ] Municipality Wedding<br>2.[ ] Religious ceremony<br>3.[ ] By elderly people<br>4.[ ] co-habiting (not legally married)
A14.	Including you, how many wives does your husband have?	└─┴─┘ wives
A15.	What number wife are you?	└─┴─┘
A16.	If currently married or cohabiting, how long have you been living with your spouse or sexual	└─┴─┘ months └─┴─┘ Years

	partner?	
A17.	How many times have you been married (civil, religious, common, customary law marriage)?	└─┴─┘ times<br>**IF 00 SKIP TO A20**
A18.	How old were you when you first got married?	└─┴─┘ Years old
A19.	The building that you sleep in – of what material are the walls made?<br>*(Tick all that apply)*<br>To be filled by observation	1. [ ] Stone, solid cement<br>2. [ ] Fired brick<br>3. [ ] Sun-dried bricks<br>4. [ ] Mud, cement<br>5. [ ] Mud, sticks, reeds<br>6. [ ] Other (specify)_______________
A20.	What is the roof of this building made of?<br>*(Tick all that apply)*<br>To be filled by observation	1. [ ] Iron<br>2. [ ] Grass or reeds<br>3. [ ] Mud, branches<br>4. [ ] Mud, cement<br>5. [ ] Other (specify)_______________
A21.	What is the floor of your house made of?<br>*(Tick all that apply)*<br>To be filled by observation	1. [ ] Cement<br>2. [ ] Mud<br>3. [ ] Cow dung/soil<br>4. [ ] Other (specify) ______________
A22.	Do you own a latrine?	1. [ ] Yes, outdoor pit latrine, shared<br>2. [ ] Yes, outdoor pit latrine, private<br>3. [ ] Yes, indoor latrine<br>4. [ ] Yes, outdoor pit latrine and indoor latrine<br>5. [ ] No, neither type of latrine **>>>skip to A25**
A23.	If you have a latrine at home, do you use the latrine nearly always, for long call only, or rarely?	1. [ ] Use it always<br>2. [ ] Use it for long call only<br>3. [ ] Use it rarely
A24.	Does your household have:<br><br>a. Electricity?<br>b. Wall Watch/clock?<br>c. A radio?<br>d. A television?<br>e. A mobile phone?<br>f. An ox?<br>g. A cow?<br>h. A table?<br>i. A chair?<br>j. A bed?<br>k. A cotton/sponge/spring mattress?	 <br>a. 1. [ ] Yes 2. [ ] No<br>b. 1. [ ] Yes 2. [ ] No<br>c. 1. [ ] Yes 2. [ ] No<br>d. 1. [ ] Yes 2. [ ] No<br>e. 1. [ ] Yes 2. [ ] No<br>f. 1. [ ] Yes 2. [ ] No<br>g. 1. [ ] Yes 2. [ ] No<br>h. 1. [ ] Yes 2. [ ] No<br>i. 1. [ ] Yes 2. [ ] No<br>j. 1. [ ] Yes 2. [ ] No<br>k. 1. [ ] Yes 2. [ ] No
A25.	If one walked at an average pace, approximately how many minutes would it take to walk from your home to the nearest place where you can catch a bus?	|__|__|__| minutes

B. Husband background information, Employment status and Women's status

B1.	How old was your husband/partner on his last birthday?	└─┴─┘ Years
B2.	Did your husband/partner ever attend school?	1. [ ] Yes<br>2. [ ] No **>>>>>>>>>skip to B5**
B3.	What was the highest level of school he attended: primary, secondary or higher?	1. [ ] Primary(grade 1-8)<br>2. [ ] Secondary (grade 9-12)<br>3. [ ] Higher (>=13)
B4.	What was the highest grade/form/year that he completed at that level?	└─┴─┘
B5.	What is your husband's/partner's occupation? That is what kind of work does he mainly do?	1. [ ] Farming<br>2. [ ] Trade<br>3. [ ] Employed (Gov/ private)<br>4. [ ] Daily laborer<br>5. [ ] Other______________
B6.	Does this household own any agricultural land?	1. [ ] Yes<br>2. [ ] No**>>>>>SKIP TO B9**
B7.	How much agricultural land does the household own?(1 Timmad is 0.25 Hectare)	|__|__| . |__|__| Timmad
B8.	What crop do you mainly grow?	1. [ ] Maize<br>2. [ ] Barely<br>3. [ ] Pepper<br>4. [ ] Teff<br>5. [ ] False banana<br>6. [ ] Bean<br>7. [ ] Khat<br>8. [ ] Coffee<br>9. [ ] Other______________
B9.	What is your husband's average monthly income?	|__|__|__|__|__| Birr
B10.	Have you ever worked or done any activities for income?	1. [ ] Yes<br>2. [ ] No **>>>>>SKIP TO B18**
B11.	What kind of work or activities to earn income have you done in the last 12 months?	__________________
B12.	Do you do this work for a member of your family, for someone else, or are yourself?	1. [ ] For family member<br>2. [ ] For someone else<br>3. [ ] For herself
B13.	Do you usually work throughout the year, or do you work seasonally, or only once in a while? Clarify	1. [ ] Throughout the year<br>2. [ ] Seasonally<br>3. [ ] Once in a while
B14.	Are you paid in cash or kind for this work or are you not paid at all?<br>Clarify	1. [ ] Cash only<br>2. [ ] In kind only<br>3. [ ] Cash and in Kind
B15.	What is your average daily wage?<br>If in Kind change to a monetary equivalent	|__|__|__| Birr

B16.	Who usually decides how the money you earn will be used: mainly you, mainly your husband/partner or you and your husband/partner jointly?	1. [ ] Respondent<br>2. [ ] Husband/partner<br>3. [ ] Respondent and husband/partner jointly<br>4. [ ] Other______________
B17.	Would you say that the money that you earn is more than what your husband/partner earns, less than what he earns or about the same?	1. [ ] More than him<br>2. [ ] Less than him<br>3. [ ] About the same<br>4. [ ] Husband/partner doesn't bring in money<br>5. [ ] Don't know
B18.	Who usually decides how your husband's/ partner's earnings will be used: you, your husband/partner, or you and your husband/partner jointly?	1. [ ] Respondent<br>2. [ ] Husband/partner<br>3. [ ] Respondent and husband/partner jointly<br>4. [ ] Other______________
B19.	How many times per day or week you participate in a traditional coffee ceremony?<br>If displayed in days, put 00 on the per-week<br>If displayed in weeks, put 00 on the per day	└─┴─┘ per day └─┴─┘ per week
B20.	Who usually participates in the coffee ceremony with you?<br><br>Tick all that apply.	1. [ ] Husband/partner<br>2. [ ] Family members<br>3. [ ] Neighbors<br>4. [ ] Village leader<br>5. [ ] Religious leader<br>6. [ ] Friends<br>7. [ ] Other______________
B21.	What do you discuss with others during a coffee ceremony?<br>Tick all that apply.	1. [ ] Family or personal issues<br>2. [ ] Issues related to your community<br>3. [ ] National or political news<br>4. [ ] Health challenges<br>5. [ ] Problems with farming or cattle<br>6. [ ] Other
B22.	Who usually prepares the coffee during the traditional coffee ceremony?	1. [ ] Men<br>2. [ ] Women
B23.	Has your husband ever prepared the coffee during the traditional coffee ceremony?	1. [ ] Yes<br>2. [ ] No
B24.	Would you be happy to participate in a coffee ceremony if a man prepared the coffee?	1. [ ] Yes<br>2. [ ] No**>>>>>SKIP TO B26**
B25.	If yes, why?	1. [ ] Men and women are equal.<br>2. [ ] Both men and women can participate fully in the coffee ceremony.<br>3. [ ] I enjoy participating without having to make coffee.<br>4. [ ] Other: ________________
B26.	If not, why not?	1. [ ] This is not customary<br>2. [ ] I prefer the company of other women.

		3. [ ] Men should not perform women's role in a coffee ceremony.<br>4. [ ] Other: ________________

B27	Now if you permit me, I will ask some more questions about your relationship with your husband.<br><br>In the last twelve months, did your husband ever:	Yes	No	Yes, how often?		
				Often	Sometimes	Rarely
	a) Expect that you ask permission to purchase large items?	1	2	1	2	3
	b) Expect that you ask permission to purchase small items?	1	2	1	2	3
	c) Take your earnings or savings from you against your will?	1	2	1	2	3
	d) Refuse to give you money for household expenses, even when he had money for other things?	1	2	1	2	3
	e) Require that you give up or refuse a job for money outside the home because your partner did not want you to work?	1	2	1	2	3
	f) Make important financial decisions without talking to you about them?	1	2	1	2	3
	g) Decide how you could spend money?	1	2	1	2	3
	h) Demand to know how you spent money?	1	2	1	2	3
	i) Hide money from you?	1	2	1	2	3
	j) Spent money you needed for rent, food or clothes on things for him?	1	2	1	2	3
	k) Threaten to not give you money or take it away from you?	1	2	1	2	3
	l) Given you little money or reduced your spacing when he is angry?	1	2	1	2	3
	m) Stopped giving you money for food?	1	2	1	2	3
	n) Taken away or used your belongings against your will?	1	2	1	2	3

B28.	How often does your husband/partner drink alcohol?	1. [ ] Every day or nearly every day<br>2. [ ] Once or twice a week<br>3. [ ] 1-3 times in a month<br>4. [ ] Rarely<br>5. [ ] Never drinks >>>>>>>> Skip to B31
B29.	In the past 12 months how often have you seen your husband/partner drunk? Would you say most days, weekly, once a month, less than once a month or never?	1. [ ] Most days<br>2. [ ] Weekly<br>3. [ ] Once a month<br>4. [ ] Very Rarely<br>5. [ ] Never drinks >>>>>>>> Skip to B31
B30.	In the past 12 months, have you experienced any of the following problems, related to your husband/partner's drinking?<br><br>a. Money problems<br>b. Health problems<br>c. Conflict with family or friends<br>d. Problems with authorities (police etc)<br>e. Any other problems (specify)	a. 1. [ ] Yes 2. [ ] No<br>b. 1. [ ] Yes 2. [ ] No<br>c. 1. [ ] Yes 2. [ ] No<br>d. 1. [ ] Yes 2. [ ] No<br><br>e. Other(Specify)_________________
B31.	How often does your husband/partner chew khat?<br>1. Every day or nearly every day<br>2. Once or twice a week<br>3. 1-3 times a month<br>4. Occasionally, less than once a month<br>5. Never	1. [ ] Every day or nearly every day<br>2. [ ] Once or twice a week<br>3. [ ] 1-3 times in a month<br>4. [ ] Very Rarely<br>5. [ ] Never >>>>>>>> Skip to C1
B32.	In the past 12 months, have you experienced any of the following problems, related to your husband/partner's khat chewing?<br><br>a. Money problems<br>b. Health problems<br>c. Conflict with family or friends<br>d. Problems with authorities (police etc)<br>Any other problems (specify)	a. 1. [ ] Yes 2. [ ] No<br>b. 1. [ ] Yes 2. [ ] No<br>c. 1. [ ] Yes 2. [ ] No<br>d. 1. [ ] Yes 2. [ ] No<br>e. Other(Specify)_________________

C. General Health and Well-being

Enumerator: *Next we would like to ask you about your general health, history of pregnancy, your experience with pregnancy and childbirth, family planning and nutrient. We assure you that the information you provide will be kept confidentially.*

C1.	On most days of the last week, how did you feel?<br>***(Read out options)***	1. [ ] Well<br>2. [ ] Tired/weak<br>3. [ ] Sick<br>4. [ ] Extremely sick
C2.	Overall, in the last 30 days, how much difficulty did you have with moving around?<br>***(Read out options)***	1. [ ] None<br>2. [ ] Mild<br>3. [ ] Moderate<br>4. [ ] Severe
C3.	In the last 30 days how much difficulty did you have in doing vigorous activities (such as walking long distances or fetching numerous buckets of water from the well).<br>***(Read out options)***	1. [ ] None<br>2. [ ] Mild<br>3. [ ] Moderate<br>4. [ ] Severe
C4.	Overall, in the last 30 days, how much difficulty did you have with concentrating or remembering things?<br>***(Read out options)***	1. [ ] None<br>2. [ ] Mild<br>3. [ ] Moderate<br>4. [ ] Severe
**FOR NEXT QUESTIONS ASK ABOUT MOST SEVERE/PERSISTENT SYMPTOM / DISEASE IN LAST 30 DAYS**		
C5.	Have you suffered from any symptoms of illness/injury in the past 30 days?	1. [ ] Yes<br>2. [ ] No **>>>>SKIP TO D1**

C6. What symptoms did you suffer from? ***(Use code below)***	C7. How many days did you suffer from this symptom?	C8. Did you seek any type of medical treatment for your health problem?	C9. Where did you seek treatment?<br>1. Own house/other home<br>2. community health worker<br>3. Health post<br>4. Primary health center<br>5. Government hospital<br>6. Private hospital<br>7. Private drug store<br>8. Other (specify)	C10. If not, why not?<br>1. Problem not serious<br>2. Treatment too costly<br>3. Distance is too far<br>4. Afraid to find serious problem<br>5 Didn't have permission<br>6. Nobody to care for kids<br>7. Didn't want to go alone<br>8. Didn't know where to go<br>9. Other	C11. Did you suffer from any other symptom of illness/injury in the past 30 days?
a. |__|__| **>> If 61 (other), specify** ____________________________	|__|__|	1. [ ] Yes **>>**<br>2. [ ] No<br>**Skip to C10**	|__| >> If 8 (other), specify: ____________________________	|__| **>>** If 9 (other), specify ____________________________	1. [ ] Yes > **Cont. to C6**<br>2. [ ] No **> Skip to D1**
b. |__|__| **>> If 61 (other), specify** ____________________________	|__|__|	1. [ ] Yes >><br>2. [ ] No<br>**Skip to C10**	|__| >> If 8 (other), specify: ____________________________	|__| >> If 9 (other), specify ____________________________	1. [ ] Yes > **Cont. to C6**<br>2. [ ] No **> Skip to D1**
c. |__|__| **>> If 61 (other), specify** ____________________________	|__|__|	1. [ ] Yes >><br>2. [ ] No<br>**Skip to C10**	|__| >> If 8 (other), specify: ____________________________	|__| >> If 9 (other), specify ____________________________	1**Skip to D1**

**GENERAL**	**GASTROINTESTINAL**	**RESPIRATORY TRACT**	**MUSCULOSKELTAL/**	**IMMUNIZABLE**
Weakness 01	Upper abdomen Pain - 15	Catarrh - 24	**NEUROLOGICAL**	Chicken pox - 46
Headache 02	Lower abdomen Pain - 16	Cough - 26	Joint pains - 37	Measles - 47
Back pain 03	Dyspepsia - 17	Cough with blood - 27	Broken bones - 38	Mumps - 48
Generalized body ache - 04	Flatulence - 18	Blood in spit - 28	Fully paralysis - 39	Tetanus - 49
Unexplained weight loss 05	Vomiting - 19	Difficulty with breathing - 29	Partial paralysis - 40	Yellow fever - 50
Unexplained Fever - 06	Diarrhea - 20	Chest pain - 30	Convulsion - 41	
Night sweats - 07	Constipation - 21	Asthma - 31	Epilepsy - 42	**OTHER**
Chills - 08	Rectal/anal pain - 22		Memory loss - 43	Anemia (iron) - 51
Swelling ankles - 09	Rectal prolapse – 23	**GENITO-URINARY**		Goiter (iodine) - 52
Eye problems - 10		Bladder/Urinary Tract - 32	Physically Disabled - 60	ARI - 53
Ear problems - 11	Blood in stool - 25	Infection - 33		Cut/ burn - 54
Throat problems - 12		Genital Ulcers - 34	**PARASITIC**	Jaundice– - 55
Skin problems - 13		STIs - 35	Worms in stool - 44	Diabetic - 56
Dental problem– - 14		Uterus (genitourinary) prolapse – 36	Fever with chills (Malaria) - 45	Boil 57
		Painful urination - 59		Tumor - 58
				[illegible]

D. Pregnancy history

D1.	How many living biological children do you have, whether or not they are living with you?	|__|__| children<br>If no child, enter 00
D2.	How many boys and how many girls?<br>If no child, enter 00 without asking this question	|__|__| boys |__|__| girls
D3.	How many biological children do you have who have died?<br>If no child, enter 00 without asking this question	|__|__| children
D4.	How old were you when you first became pregnant?<br>If no child, enter 00 without asking this question	|__|__| years<br>Enter 00, if never pregnant
D5.	How old were you when you last became pregnant?<br>If no child, enter 00 without asking this question	|__|__| years<br>Enter 00, if never pregnant
D6.	Have you ever had a miscarriage?	1. [ ] Yes<br>2. [ ] No>>>>>>>>>>>>>SKIP TO D8
D7.	If yes, how many?	|__|__|
D8.	Have you ever had a stillbirth?	1. [ ] Yes<br>2. [ ] No>>>>>>>>>>>>>SKIP TO D10
D9.	If yes, how many?	|__|__|
D10.	Are you pregnant now?	1. [ ] Yes<br>2. [ ] No
D10b	How many of the pregnancies resulted to twin or triple children?	|__|__|
D11	This means you have been pregnant xx times, is that correct?<br>*Enumerator:*<br>*Add response to [(D1+ D3 + D7.+ D9 + D10) -D10b] to fill in xx.*	1. [ ] Yes<br>2. [ ] No<br>*If incorrect answer, go to D1 and ask again.*
D12	What age is your pregnancy now?<br>**If the answer to D10 is Yes, ask this question**<br>If answered in weeks, convert to months	|__|__| months<br>If currently not pregnant skip to D17
D13	During your pregnancy, who have you seen for antenatal care?	1. [ ] Female health professional<br>2. [ ] Male health professional<br>3. [ ] Health extension worker<br>4. [ ] No one >>>>>>SKIP TO D16<br>5. [ ] Other ________________________

D14	Where have you received antenatal care?	1. [ ] Govt hospital<br>2. [ ] Govt. health center<br>3. [ ] Govt health post<br>4. [ ] Private clinic<br>5. [ ] Private hospital<br>6. [ ] Own house/relative house<br>7. [ ] Other ______________________
D15	How many times have you received antenatal care?	└─┴─┘ times
D16	Have you suffered from the following symptoms during your pregnancy?<br>Tick all that apply.	1. [ ] Convulsions<br>2. [ ] Swelling of legs, body, or face<br>3. [ ] Excessive fatigue<br>4. [ ] Vaginal bleeding<br>5. [ ] Night blindness<br>6. [ ] Difficulty with vision during daylight<br>7. [ ] Other (specify)<br>____________________<br>8. [ ] No symptom
D17	Have you been pregnant within the last 3 years?	1. [ ] Yes<br>2. [ ] No>>>>>>>>>>>>>SKIP TO E1
D18	During your last pregnancy, who did you see for antenatal care?	1. [ ] Female health professional<br>2. [ ] Male health professional<br>3. [ ] Health extension worker<br>4. [ ] No one >>>>>>SKIP TO D16<br>5. [ ] Other ______________________
D19	Where did you receive antenatal care?	1. [ ] Govt hospital<br>2. [ ] Govt. health center<br>3. [ ] Govt health post<br>4. [ ] Private clinic<br>5. [ ] Private hospital<br>6. [ ] Own house/relative house<br>7. [ ] Other ______________________
D20	How many times did you receive antenatal care?	└─┴─┘ times
D21	Did you suffer from the following symptoms during your pregnancy?<br>Tick all that apply.	1. [ ] Convulsions<br>2. [ ] Swelling of legs, body, or face<br>3. [ ] Excessive fatigue<br>4. [ ] Vaginal bleeding<br>5. [ ] Night blindness<br>6. [ ] Difficulty with vision during daylight<br>7. [ ] Other (specify)_________________<br>8. [ ] None
D22	What was the outcome of your pregnancy?	1. [ ] Miscarriage>>>>>>>>>SKIP TO E1<br>2. [ ] Stillbirth>>>>>>>>>>>SKIP TO E1<br>3. [ ] Live birth
D23	What was the name given to this child?<br>Is (NAME) a boy or a girl?	Name: ________________<br>a) [ ] Boy<br>b) [ ] Girl

D24	Is (NAME) still alive?	1. [ ] Yes<br>2. [ ] No>>>>>>>>>>>>SKIP TO D26
D25	How old was (NAME) at last birthday	└─┴─┘ years<br>└─┴─┘ months old if less than 1 year<br><br>└─┴─┘ days old if less than 1 month
D26	Where did you give birth?	1. [ ] Govt hospital<br>2. [ ] Govt. health center<br>3. [ ] Govt health post<br>4. [ ] Private clinic<br>5. [ ] Private hospital<br>6. [ ] Own house/relative house<br>7. [ ] Other _______________________
D27	Who assisted in the delivery?	1. [ ] Female health professional<br>2. [ ] Male health professional<br>3. [ ] Health extension worker<br>4. [ ] Traditional Birth Attendant<br>5. [ ] No one<br>5. [ ] Other _______________________
D28	Was your child delivered by Caesarean section?	1. [ ] Yes<br>2. [ ] No
D29	How was the delivery process (Delivery using Cesarean session)?<br>Only if she delivers in a hospital and continue for presence of a scare on abdomen	1. [ ] Normal<br>2. [ ] Complicated

E. Family planning

E1.	Now I would like to talk to you about family planning- the various ways or methods that a couple can use to delay or avoid a pregnancy. Which methods have you heard about?<br><br>***(Do not read out options. Record all mentioned.)***	1. [ ] Pill<br>2. [ ] Intrauterine Device (IUD) or Loop<br>3. [ ] Norplant/Implant<br>4. [ ] Injectables<br>5. [ ] Diaphragm<br>6. [ ] Male condom<br>7. [ ] Female condom<br>8. [ ] Rhythm method<br>9. [ ] Withdrawal<br>10. [ ] Lactational amenorrhoea<br>11. [ ] Emergency contraception<br>12. [ ] Traditional Herbs<br>13. [ ] Other ______________________<br>14. [ ] Don't know >>>>>>>>>>>SKIP TO E4<br>15. [ ] Refused to answer
E2.	Are you or your husband/partner using family planning methods to postpone or prevent pregnancy?	1. [ ] Yes<br>2. [ ] No **>>>>>>>>>>SKIP TO E4**<br>3. [ ] Refused to answer **>>>>>SKIP TO E4**
E3.	What types of methods are you/your husband	1. [ ] Pill

	using?<br>***(Do not read our options. Record all mentioned.)***<br>***(If injectables, ask how frequently the woman has to take the injection.)***	2. [ ] Intrauterine Device (IUD) or Loop<br>3. [ ] Norplant/Implant<br>4. [ ] Diaphragm<br>5. [ ] 3-monthly injection<br>6. [ ] Male condom<br>7. [ ] Female condom<br>8. [ ] Rhythm method<br>9. [ ] Withdrawal<br>10. [ ] Lactational amenorrhoea<br>11. [ ] Emergency contraception<br>12. [ ] Traditional Herbs<br>13. [ ] Other ______________________<br>14. [ ] Don't know >>>>>>>>>>><br>15. [ ] Refused to answer
E4.	Do you think you will use a contraceptive method to delay or avoid pregnancy at any time in the future?	1. [ ]Yes **>>>>SKIP TO E6**<br>2. [ ]No<br>3. [ ]Don't know<br>4. [ ]Refused to answer **>>>> SKIP TO E6**
E5.	What is the main reason you think you will not use a contraceptive method at any time in the future?<br><br>Only one answer allowed	1. [ ] Infrequent sex/no sex<br>2. [ ] Menopausal/hysterectomy<br>3. [ ] Subfecund/infecund<br>4. [ ] Wants more children<br>5. [ ] Respondent opposed to use<br>6. [ ] Husband opposed to use<br>7. [ ] Others opposed to use<br>8. [ ] Religious prohibition<br>9. [ ] Knows no method<br>10. [ ] Knows no source<br>11. [ ] Health concerns<br>12. [ ] Fear of side effects<br>13. [ ] Lack of access/too far<br>14. [ ] Costs too much<br>15. [ ] Inconvenient to use<br>16. [ ] Lactational amenorrhea<br>17. [ ] Other _________________
E6.	If you could go back to the time prior to your marriage and choose the number of children to have in your whole life, how many would that be?	|__|__| children

F. Gender and Domestic Violence

Enumerator: Now, I am going to read you a list of statements. For each statement, I would like you to first tell me if you agree with this statement yourself, and next tell me if you think a majority (more than half) of your friends and family agree with this statement. For example, I could read the statement "It is beneficial for all children to attend primary school." First, you would say "Yes" if you agree with this statement yourself. Next, if you believe that the majority of your friends and families also agree, you would say "Yes" again.

		(a) Do you agree with this statement?	(b) Do your friends and family agree with this statement?
F1.	A man should have the final word on decisions in his home.	1. [ ]Yes<br>2. [ ]No	1. [ ]Yes<br>2. [ ]No
F2.	A woman should obey her husband in all things.	1. [ ]Yes<br>2. [ ]No	1. [ ]Yes<br>2. [ ]No
F3.	It must be the man who provides for the family.	1. [ ]Yes<br>2. [ ]No	1. [ ]Yes<br>2. [ ]No
F4.	The husband should decide what major household items to buy	1. [ ]Yes<br>2. [ ]No	1. [ ]Yes<br>2. [ ]No
F5.	If a wife does something wrong her husband has the right to punish her.	1. [ ]Yes<br>2. [ ]No	1. [ ]Yes<br>2. [ ]No
F6.	A woman should be able to choose her own friends even if her husband disapproves	1. [ ]Yes<br>2. [ ]No	1. [ ]Yes<br>2. [ ]No
F7.	A woman should be able to get help from a skilled birth attendant when she needs it, even without her husband's approval.	1. [ ]Yes<br>2. [ ]No	1. [ ]Yes<br>2. [ ]No
F8.	A husband and wife should make decisions together about health care for themselves.	1. [ ]Yes<br>2. [ ]No	1. [ ]Yes<br>2. [ ]No
F9.	A husband and wife should make decisions together about health care for their children.	1. [ ]Yes<br>2. [ ]No	1. [ ]Yes<br>2. [ ]No
F10.	Village assemblies and local development committee members should be mainly men because women don't have a voice in village matters.	1. [ ]Yes<br>2. [ ]No	1. [ ]Yes<br>2. [ ]No
F11.	A man needs other women, even if things with his wife are fine.	1. [ ]Yes<br>2. [ ]No	1. [ ]Yes<br>2. [ ]No
F12.	You don't talk about sex, you just do it.	1. [ ]Yes<br>2. [ ]No	1. [ ]Yes<br>2. [ ]No
F13.	Women who carry condoms on them are easy.	1. [ ]Yes<br>2. [ ]No	1. [ ]Yes<br>2. [ ]No
F14.	Men need sex more than women do.	1. [ ]Yes<br>2. [ ]No	1. [ ]Yes<br>2. [ ]No
F15.	Men are always ready to have sex.	1. [ ]Yes<br>2. [ ]No	1. [ ]Yes<br>2. [ ]No

F16.	A woman should not initiate sex.	1. [ ]Yes<br>2. [ ]No	1. [ ]Yes<br>2. [ ]No
F17.	It is the man who decides what type of sex to have.	1. [ ]Yes<br>2. [ ]No	1. [ ]Yes<br>2. [ ]No
F18.	A man should be outraged if his wife asks him to use a condom.	1. [ ]Yes<br>2. [ ]No	1. [ ]Yes<br>2. [ ]No
F19.	A woman can suggest using condoms just like a man can.	1. [ ]Yes<br>2. [ ]No	1. [ ]Yes<br>2. [ ]No
F20.	A married woman should not refuse to have sex if her husband wants to.	1. [ ]Yes<br>2. [ ]No	1. [ ]Yes<br>2. [ ]No
F21.	It is a woman's responsibility to avoid getting pregnant.	1. [ ]Yes<br>2. [ ]No	1. [ ]Yes<br>2. [ ]No
F22.	A couple should decide together if they want to have children.	1. [ ]Yes<br>2. [ ]No	1. [ ]Yes<br>2. [ ]No
F23.	If a man gets a woman pregnant, the child is the responsibility of both.	1. [ ]Yes<br>2. [ ]No	1. [ ]Yes<br>2. [ ]No
F24.	A woman who has sex before she marries does not deserve respect.	1. [ ]Yes<br>2. [ ]No	1. [ ]Yes<br>2. [ ]No
F25.	Only when a woman has a child is she a real woman.	1. [ ]Yes<br>2. [ ]No	1. [ ]Yes<br>2. [ ]No
F26.	A real man produces a male child.	1. [ ]Yes<br>2. [ ]No	1. [ ]Yes<br>2. [ ]No
F27.	Parents should be happy if their daughter is going to be married at any age – even if she is younger than 18 years old.	1. [ ]Yes<br>2. [ ]No	1. [ ]Yes<br>2. [ ]No
F28.	Women are sometimes to blame for violence against them.	1. [ ]Yes<br>2. [ ]No	1. [ ]Yes<br>2. [ ]No
F29.	Women should not tolerate violence just in order to keep her family together.	1. [ ]Yes<br>2. [ ]No	1. [ ]Yes<br>2. [ ]No
F30.	No one outside the couple should intervene if a husband beats up his wife.	1. [ ]Yes<br>2. [ ]No	1. [ ]Yes<br>2. [ ]No
F31.	There are times a woman deserves to be beaten.	1. [ ]Yes<br>2. [ ]No	1. [ ]Yes<br>2. [ ]No
F32.	A woman who intervenes when a husband is beating his wife is meddling.	1. [ ]Yes<br>2. [ ]No	1. [ ]Yes<br>2. [ ]No

FO: Now, some people may believe that a man has a good reason to hit his wife in certain situations. I am going to read you a list of situations. For each one, please tell me if you believe that a man has a good reason to hit his wife in this situation. Please also tell me if you think most of your friends and families agree that a man would have a good reason to hit his wife in this situation.

	Good reason for a husband to beat his wife…..	(a) Do you agree a man would have a good reason to hit his wife in this situation?	(b) Would your friends and family agree a man has a good reason to hit his wife in this situation?
F33.	She disobeys him.	1. [ ]Yes<br>2. [ ]No	1. [ ]Yes<br>2. [ ]No
F34.	She answers back to him.	1. [ ]Yes<br>2. [ ]No	1. [ ]Yes<br>2. [ ]No
F35.	She disrespects his relatives.	1. [ ]Yes<br>2. [ ]No	1. [ ]Yes<br>2. [ ]No
F36.	He suspects that she is unfaithful.	1. [ ]Yes<br>2. [ ]No	1. [ ]Yes<br>2. [ ]No
F37.	He finds out that she has been unfaithful.	1. [ ]Yes<br>2. [ ]No	1. [ ]Yes<br>2. [ ]No
F38.	She spends her time gossiping with neighbors.	1. [ ]Yes<br>2. [ ]No	1. [ ]Yes<br>2. [ ]No
F39.	She neglects taking care of the children.	1. [ ]Yes<br>2. [ ]No	1. [ ]Yes<br>2. [ ]No
F40.	She does not complete her household work to his satisfaction.	1. [ ]Yes<br>2. [ ]No	1. [ ]Yes<br>2. [ ]No
F41.	She accuses him of infidelity.	1. [ ]Yes<br>2. [ ]No	1. [ ]Yes<br>2. [ ]No
F42.	She tells his secrets to others in the community.	1. [ ]Yes<br>2. [ ]No	1. [ ]Yes<br>2. [ ]No
F43.	He is angry with her.	1. [ ]Yes<br>2. [ ]No	1. [ ]Yes<br>2. [ ]No
F44.	She burns the food.	1. [ ]Yes<br>2. [ ]No	1. [ ]Yes<br>2. [ ]No
F45.	She goes out without telling him.	1. [ ]Yes<br>2. [ ]No	1. [ ]Yes<br>2. [ ]No

FO: Now I would like to ask you questions about some other important aspects of a woman's life. I know some of these questions are very personal. However, your answers are crucial for helping to understand the condition of women in Ethiopia. Let me assure you that your answers are completely confidential and will not be told to anyone and no one else will know that you were asked these questions.

F46.	Do you think violence against women is a major problem in your village?	1. [ ] YES<br>2. [ ] NO<br>3. [ ] Don't Know
F47.	Out of 10 women living in your village, how many do you think are experiencing any type of physical violence from their husbands?	└─┴─┘ women
F48.	Out of 10 women living in your village, how many do you think are experiencing any type of sexual violence from their husbands?	└─┴─┘ women
F49.	What are some of the consequences of violence against women?<br>Tick all that are mentioned.<br>(**Do not read options**)	1. [ ] Psychological outcomes<br>2. [ ] Physical injuries<br>3. [ ] Sexual and reproductive health problems<br>4. [ ] Suicide or death<br>5. [ ] Health or psychological risks for children<br>6. [ ] HIV or STI<br>7. [ ] Other (Specify)__________________<br>8. [ ] Don't Know
F50.	How does violence against women increase their vulnerability to HIV/AIDS?<br>Tick all that are mentioned.<br>(**Do not read options**)	1. [ ] Limits ability to negotiate safe sex<br>2. [ ] Violent men may engage in risky sexual behaviors and put their spouses at risk<br>3. [ ] Limits access to information and treatment<br>4. [ ] Increases risk of transmission to infants<br>5. [ ] Other (Specify)_________________
F51.	If a woman in your village was experiencing violence from her husband, who could she go to for help?<br>Tick all that are mentioned.<br>(**Do not read options**)	1. [ ] Female relative/friend<br>2. [ ] Male relative<br>3. [ ] Police officer<br>4. [ ] Health worker<br>5. [ ] Religious leader<br>6. [ ] Community leader<br>7. [ ] Neighbor<br>8. [ ] Other: ________________________
F52.	If a woman in your village told you that she was experiencing violence from her husband, what would you do? Tick all that apply.	1. [ ] Discuss the problem of violence with her<br>2. [ ] Encourage her to visit a health facility for treatment<br>3. [ ] Encourage her to report violence to a police officer<br>4. [ ] Encourage her to discuss this problem with traditional / religious leaders<br>5. [ ] Inform your husband about your conversation<br>6. [ ] Inform other women in the village about your

		conversation<br>7 [ ] Speak to the woman's husband or family about the problem of violence.<br>8. [ ] Nothing, since we should not interfere in other people's private lives.<br>9. [ ] Tell her that violence is common/normal and she should accept it.<br>10. [ ] Discourage her from telling other people<br>11. [ ] Advise her to resolve conflict elicited with her husband<br>12. [ ] Other: ______________________________
F53.	I am going to read some statements about violence against women. For each statement, please state whether you agree or disagree.<br><br>a) If a man does not give his wife money for household necessities, even though he spends money on things for himself, it is an example of violence against women.<br><br>b) If a man forbids his wife from seeing his parents and other family members, or friends, it is not an example of violence against women.<br><br>c) If a man slaps, pushes, shoves, kicks or drags a woman, including his wife, it is not an example of violence against women.<br><br>d) If a man threatens to hit a woman, but does not actually hit her, it is an example of violence against women.<br><br>e) If a man forces a woman he does not know to have sex, it is an example of violence against women.<br><br>f) If a man forces his wife to have sex, it is not an example of violence against women.<br><br>g) If a man shouts at his wife in public and embarrasses her, it is an example of violence against women.	a) 1. [ ] Agree 2. [ ] Disagree<br><br>b) 1. [ ] Agree 2. [ ] Disagree<br><br>c) 1. [ ] Agree 2. [ ] Disagree<br><br>d) 1. [ ] Agree 2. [ ] Disagree<br><br>e) 1. [ ] Agree 2. [ ] Disagree<br><br>f) 1. [ ] Agree 2. [ ] Disagree<br><br>g) 1. [ ] Agree 2. [ ] Disagree

<table>
<tr><td></td><td colspan="6">When two people marry or live together, they usually share both good and bad moments. I would now like to ask you some questions about your current and past relationships and how your husband/partner treats you. If anyone interrupts us I will change the topic of conversation. I would again like to assure you that your answers will be kept secret, and that you do not have to answer any questions that you do not want to. May I continue?</td></tr>
<tr><td>F54.</td><td>In general, do you and your husband/partner discuss the following topics together:
a) things that have happened to him in the day
b) Things that happened to you during the day
c) Your worries or feelings
d) His worries or feelings</td><td colspan="5">a) 1. [ ] Yes 2. [ ] No
b) 1. [ ] Yes 2. [ ] No
c) 1. [ ] Yes 2. [ ] No
d) 1. [ ] Yes 2. [ ] No</td></tr>
<tr><td>F55.</td><td>In your relationship with your husband/partner, how often would you say that you quarreled? Would you say rarely, sometimes or often?</td><td colspan="5">1. [ ] Rarely
2. [ ] Sometimes
3. [ ] Often</td></tr>
<tr><td>F56.</td><td>I am now going to ask you about some situations that are true for many women. Thinking about your current husband/partner, would you say it is generally true that he:
a) Tries to keep you from seeing your friends?
b) Tries to restrict contact with your family of birth?
c) Insists on knowing where you are at all times?
d) Ignores you and treats you indifferently?
e) Gets angry if you talked to other men?
f) Is often suspicious that you are unfaithful?
g) Expects you to ask his permission before leaving home?
h) Expects you to ask his permission before seeking health care for yourself?
i) Prevent you from expressing your opinion in public?</td><td colspan="5">a) 1. [ ] Yes 2. [ ] No
b) 1. [ ] Yes 2. [ ] No
c) 1. [ ] Yes 2. [ ] No
d) 1. [ ] Yes 2. [ ] No
e) 1. [ ] Yes 2. [ ] No
f) 1. [ ] Yes 2. [ ] No
g) 1. [ ] Yes 2. [ ] No
h) 1. [ ] Yes 2. [ ] No
i) 1. [ ] Yes 2. [ ] No</td></tr>
<tr><td rowspan="2">F57.</td><td rowspan="2">Now, I'd like to ask you some questions about how your husband interacts with you. In the <u>last twelve months</u>, did your husband ever:</td><td colspan="2"></td><td colspan="3">If YES, How often did this happen <u>in the last 12 months</u>: often, only sometimes or rarely.</td></tr>
<tr><td>YES</td><td>N
O</td><td>Often</td><td>Sometimes</td><td>Rarely</td></tr>
</table>

	a) Insulted you or made you feel bad about yourself<br><br>b) Belittled or humiliated you in front of other people?<br><br>c) Do things to scare or intimidate you on purpose (eg by the way he looked at you, by yelling and smashing things)?<br><br>d) Threatened to hurt you or someone you care about?	a) 1. [ ] Yes<br>2. [ ] No<br><br>b) 1. [ ] Yes<br>2. [ ] No<br><br>c) 1. [ ] Yes<br>2. [ ] No<br><br>d) 1. [ ] Yes<br>2. [ ] No	aa) 1. [ ] Often<br>2. [ ] Sometimes<br>3. [ ] Rarely<br><br>bb) 1. [ ] Often<br>2. [ ] Sometimes<br>3. [ ] Rarely<br><br>cc) 1. [ ] Often<br>2. [ ] Sometimes<br>3. [ ] Rarely<br><br>dd) 1. [ ] Often<br>2. [ ] Sometimes<br>3. [ ] Rarely

F58.	Has your your husband ever done any of the following things to you?	A.<br>**If Yes, continue with B. If NO, skip to next item.**	B.<br>Has this happened in the past 12 months?<br><br>**(If YES ask C only. If NO ask D only.)**	C.<br>**In the past 12 months** would you say this has happened<br>1 many time<br>2.few times<br>3 once,?<br>**(After answering C, skip D)**	D.<br>**Before the past 12 months** would you say that this happened<br>1 many time<br>2.few times<br>3 once,?
				**1 2 3**<br>**Many Few One**	**1 2 3**<br>**Many Few One**
	a) Slapped you or thrown something at you that could hurt you?	a) 1. [ ] Yes<br>2. [ ] No	a) 1. [ ] Yes<br>2. [ ] No	a) 1. [ ]<br>2. [ ]<br>3. [ ]	a) 1. [ ]<br>2. [ ]<br>3. [ ]
	b) Pushed you or shoved you?	b) 1. [ ] Yes<br>2. [ ] No	b) 1. [ ] Yes<br>2. [ ] No	b) 1. [ ]<br>2. [ ]<br>3. [ ]	b) 1. [ ]<br>2. [ ]<br>3. [ ]
	c) Hit you with his fist or something that could hurt you?	c) 1. [ ] Yes<br>2. [ ] No	c) 1. [ ] Yes<br>2. [ ] No	c) 1. [ ]<br>2. [ ]<br>3. [ ]	c) 1. [ ]<br>2. [ ]<br>3. [ ]
	d) Kicked you, dragged you or beaten you up?	d) 1. [ ] Yes<br>2. [ ] No	d) 1. [ ] Yes<br>2. [ ] No	d) 1. [ ]<br>2. [ ]<br>3. [ ]	d) 1. [ ]<br>2. [ ]<br>3. [ ]
	e) Choked or burnt you on purpose?	e) 1. [ ] Yes<br>2. [ ] No	e) 1. [ ] Yes<br>2. [ ] No	e) 1. [ ]<br>2. [ ]<br>3. [ ]	e) 1. [ ]<br>2. [ ]<br>3. [ ]

	f) Threatened to use or actually used a gun, knife or other weapon against you?<br><br>g) Physically force you to have sexual intercourse with him even when you did not want to?<br><br>h) Force you to perform sexual acts you did not want to?<br><br>i) Did you ever have sexual intercourse because you were intimidated by him or afraid he would hurt you?<br><br>**Note: If the respondent reports no violence, SKIP**	f) 1. [ ] Yes<br>2. [ ] No<br><br>g) 1. [ ] Yes<br>2. [ ] No<br><br>h) 1. [ ] Yes<br>2. [ ] No<br><br>i) 1. [ ] Yes<br>2. [ ] No	f) 1. [ ] Yes<br>2. [ ] No<br><br>g) 1. [ ] Yes<br>2. [ ] No<br><br>h) 1. [ ] Yes<br>2. [ ] No<br><br>i) 1. [ ] Yes<br>2. [ ] No	f) 1. [ ]<br>2. [ ]<br>3. [ ]<br><br>g) 1. [ ]<br>2. [ ]<br>3. [ ]<br><br>h) 1. [ ]<br>2. [ ]<br>3. [ ]<br><br>i) 1. [ ]<br>2. [ ]<br>3. [ ]	f) 1. [ ]<br>2. [ ]<br>3. [ ]<br><br>g) 1. [ ]<br>2. [ ]<br>3. [ ]<br><br>h) 1. [ ]<br>2. [ ]<br>3. [ ]<br><br>i) 1. [ ]<br>2. [ ]<br>3. [ ]
F59.	Did your husband ever beat or assault you while you were pregnant?	1. [ ] YES<br>2. [ ] NO			
F60.	As the result of actions by your husband, have you suffered any of the following in the last twelve months?<br><br>a) Cuts, bruises or aches?<br><br>b) Burns?<br><br>c) Deep wounds, broken bones, broken teeth or any other serious injury<br><br>d) Loss of consciousness or passing out?<br><br>e) Other	a) 1. [ ] Yes 2. [ ] No<br><br>b) 1. [ ] Yes 2. [ ] No<br><br>c) 1. [ ] Yes 2. [ ] No<br><br>d) 1. [ ] Yes 2. [ ] No<br><br>e) Specify ____________________			
F61.	In the past twelve months, were you ever treated badly enough by your husband that you needed health care (even if you didn't receive it)?	1. [ ] YES<br>2. [ ] NO			

F62.	In the past twelve months, have you ever had to spend any nights in a hospital due to the injury/injuries?	1. [ ] YES<br>2. [ ] NO
F63.	Are there any particular situations that tend to lead to violence? **Do not read options.**<br><br>Probe: Any other situation?<br><br>MARK ALL MENTIONED	1. [ ] No particular reason<br>2. [ ] When man drunk<br>3. [ ] Money problems<br>4. [ ] Difficulties at his work<br>5. [ ] When he is unemployed<br>6. [ ] No food at home<br>7. [ ] Problems with his or her family<br>8. [ ] She is pregnant<br>9. [ ] He is jealous of her<br>10. [ ] She refuses sex<br>11. [ ] She is disobedient<br>12. [ ] Other:________________________
F64.	In the past twelve months, were your children ever present when your husband mistreated you?	1. [ ] YES<br>2. [ ] NO
F65.	In the past twelve months, did mistreatment by your partner result in any of the following consequences?<br>a) The violence disrupted your household work?<br><br>b) The violence disrupted work outside the household or income-generating activities?<br><br>c) The violence disrupted your ability of take care of your children?<br><br>d) The violence caused your children to miss school?<br><br>e) The violence caused you to leave home, even if only for a night?	a) 1. [ ] Yes 2. [ ] No<br><br>b) 1. [ ] Yes 2. [ ] No<br><br>c) 1. [ ] Yes 2. [ ] No<br><br>d) 1. [ ] Yes 2. [ ] No<br><br>e) 1. [ ] Yes 2. [ ] No
F66.	Have you ever disclosed to anyone that your husband mistreats you?	1. [ ] YES<br>2. [ ] NO **>>>>SKIP TO F69**
F67.	When did you disclose this information?<br>*If more than once, record the date of the most recent disclosure.*	└─┴─┘ months └─┴─┘ years
F68.	Who did you disclose this mistreatment to?	1. [ ] No one<br>2. [ ] Friends

	**>>>>If respondent reports disclosure, SKIP TO F70**<br><br>[Check all that apply]	3. [ ] Parents<br>4. [ ] Uncle or aunt<br>5. [ ] Husband/partner's family<br>6. [ ] Children<br>7. [ ] Neighbors<br>8. [ ] Police<br>9. [ ] Doctor/Health worker<br>10. [ ] Priest/religious leader<br>11. [ ] Counselor<br>12. [ ] NGO/Women's organization<br>13. [ ] Local leader<br>14. [ ] Other ___________________________
F69.	If you did not disclose this mistreatment, which of these reasons were relevant to your decision not to disclose? For each statement, please state whether or not this was a factor in your decision.<br>a) You don't have anyone to confide in.<br>b) You don't know where to go for help.<br>c) You were embarrassed by what took place.<br>d) You didn't want other community members to know.<br>e) You never considered it a big problem.<br>f) You fear people will blame you.<br>g) You were afraid it would worsen the situation.<br>h) You felt you could take care of it yourself.<br>i) Family/friends discouraged getting help.<br>j) You believed the violence would stop.<br>k) You believe violence is a normal point of relationships.	a) 1. [ ] Yes 2. [ ] No<br>b) 1. [ ] Yes 2. [ ] No<br>c) 1. [ ] Yes 2. [ ] No<br>d) 1. [ ] Yes 2. [ ] No<br>e) 1. [ ] Yes 2. [ ] No<br>f) 1. [ ] Yes 2. [ ] No<br>g) 1. [ ] Yes 2. [ ] No<br>h) 1. [ ] Yes 2. [ ] No<br>i) 1. [ ] Yes 2. [ ] No<br>j) 1. [ ] Yes 2. [ ] No<br>k) 1. [ ] Yes 2. [ ] No<br>l) 1. [ ] Yes 2. [ ] No<br>m) 1. [ ] Yes 2. [ ] No<br>n) 1. [ ] Yes 2. [ ] No<br><br>>>>>>>>>> SKIP to F71

	l) You were afraid it would end your relationship.<br><br>m) You were afraid you would lose children.<br>n) You were watched by your partner all the time.<br>If F69 is answered or tried to be answered	
F70.	DId anyone ever tried to help you? If yes, who helped you?<br><br>MARK ALL MENTIONED<br><br>PROBE: Anyone else?	1. [ ] Village elders<br>2. [ ] Friends<br>3. [ ] Parents<br>4. [ ] Uncle or aunt<br>5. [ ] Husband/partner's family<br>6. [ ] Children<br>7. [ ] Neighbors<br>8. [ ] Police<br>9. [ ] Doctor/Health worker<br>10. [ ] Priest<br>11. [ ] Counselor/religious leader<br>12. [ ] NGO/Women's organization<br>13. [ ] Local leader<br>14. [ ] Other ___________________________<br>15. [ ] No one
F71.	Did you ever go to any of the following for help?<br><br>READ EACH ONE, AND MARK ALL MENTIONED	1. [ ] Police<br>2. [ ] Hospital/health center<br>3. [ ] Social services<br>4. [ ] Legal Services<br>5. [ ] Legal advice center<br>6. [ ] Court<br>7. [ ] Shelter<br>8. [ ] Local Leader<br>9. [ ] Women's organization: ______________________<br>10. [ ] Priest/religious leader<br>11. [ ] Elsewhere: _________________________________<br>12. [ ] No where >>>>>> **SKIP TO F73**
F72.	What were the reasons that made you go for help?<br><br>**Do not read options aloud, tick all mentioned.**	1. [ ] Encouraged by friends/family<br>2. [ ] Could not endure more<br>3. [ ] Badly injured/afraid he would kill her<br>4. [ ] He threated or tried to kill her<br>5. [ ] He threatened or hit children<br>6. [ ] Saw that children suffering<br>7. [ ] Thrown out of home<br>8. [ ] Afraid he would kill her<br>9. [ ] Other: ______________________<br>If any of the above is answered SKIP TO F74
F73.	Why did you not go to any of these?<br><br>**Do not read options aloud, tick all mentioned.**	1. [ ] Don't know/no answer<br>2. [ ] Fear of threats/consequences/more violence<br>3. [ ] Violence normal/not serious<br>4. [ ] Embarrassed/ashamed/afraid would not be believed or would be blamed

		5. [ ] Afraid would end relationship<br>6. [ ] Afraid would lose children<br>7. [ ] Bring bad name to family<br>8. [ ] Other:____________________
F74.	Did you ever leave, even if only overnight because of the violence? If yes, how many times?	|__|__| times<br>00 If never left home<br>If 00 is entered, skip to F78
F75.	Where did you go last time?<br><br>Mark only one.	1. [ ] Her relatives<br>2. [ ] His relatives<br>3. [ ] Her friends/neighbors<br>4. [ ] Hotel/lodging<br>5. [ ] Street<br>6. [ ] Church/mosque<br>7. [ ] Shelter<br>8. [ ] Other:_________________________
F76.	How long did you stay away the last time?	Number of days |__|__| Number of months |__|__|
F77.	Why did you return?<br><br>**Do not read options aloud, tick all mentioned.**	1. [ ] Didn't want to leave children<br>2. [ ] Sanctity of marriage<br>3. [ ] For sake of family/children<br>4. [ ] Couldn't support children<br>5. [ ] Loved him<br>6. [ ] He asked her to come back<br>7. [ ] Family said to return<br>8. [ ] Forgave him<br>9. [ ] Thought he would change<br>10. [ ] Threatened her/children<br>11. [ ] Could not stay where she went<br>12. [ ] Other: _________________
	In their lives, many women experience different forms of violence from their relatives, other people that they know and/or from strangers. If you don't mind I would like to briefly ask you about some of these situations. Everything that you say will be kept private. May I continue?	
F78.	From the time you were 15 years old has anyone other than your husband hit, slapped, kicked or done anything else to hurt you physically?	1. [ ] YES<br>2. [ ] NO **>>>>>>>>>>>SKIP TO F81**<br>3. [ ] Refused to answer/no answer **>>>SKIP TO F81**
F79.	Who has hurt you in this way? Anyone else?<br>Record all mentioned	1. [ ] Mother/step-mother<br>2. [ ] Father/Step-father<br>3. [ ] Sister/Brother<br>4. [ ] Daughter/Son<br>5. [ ] Other Relative<br>6. [ ] Former husband/partner<br>7. [ ] Current boyfriend<br>8. [ ] Former boyfriend<br>9. [ ] Mother-in-Law

		10. [ ] Father-in-Law<br>11. [ ] Other In-Law<br>12. [ ] Teacher<br>13. [ ] Employer/Someone at work<br>14. [ ] Police/Soldier<br>15. [ ] Other ______________________
F80.	In the last 12 months, how often have you been hit, slapped, kicked, or physically hurt by this/these people: often, only sometimes, or not at all?	1. [ ] Often<br>2. [ ] Sometimes<br>3. [ ] Rarely<br>4. [ ] Not at all
F81.	At any time in your life before you were fifteen years old, did anyone ever forced you in any way to have sexual intercourse or perform any other sexual acts?	1. [ ] YES<br>2. [ ] NO **>>>>>>>>>>>>SKIP TO F83**<br>3. [ ] Refused to answer/no answer
F82.	Who was the person who was forcing you at that time?	1. [ ] Mother/step-mother<br>2. [ ] Father/Step-father<br>3. [ ] Sister/Brother<br>4. [ ] Daughter/Son<br>5. [ ] Other Relative<br>6. [ ] Former husband/partner<br>7. [ ] Current boyfriend<br>8. [ ] Former boyfriend<br>9. [ ] Mother-in-Law<br>10. [ ] Father-in-Law<br>11. [ ] Other In-Law<br>12. [ ] Teacher<br>13. [ ] Employer/Someone at work<br>14. [ ] Police/Soldier<br>15. [ ] Other ______________________
F83.	At any time in your life since you were fifteen years old, did anyone ever force you in any way to have sexual intercourse or perform any other sexual acts?	1. [ ] YES<br>2. [ ] NO **>>>>>>>>>>>>SKIP TO F85**<br>3. [ ] Refused to answer/no answer
F84.	Who was the person who was forcing you at that time?	1. [ ] Mother/step-mother<br>2. [ ] Father/Step-father<br>3. [ ] Sister/Brother<br>4. [ ] Daughter/Son<br>5. [ ] Other Relative<br>6. [ ] Former husband/partner<br>7. [ ] Current boyfriend<br>8. [ ] Former boyfriend<br>9. [ ] Mother-in-Law<br>10. [ ] Father-in-Law<br>11. [ ] Other In-Law<br>12. [ ] Teacher<br>13. [ ] Employer/Someone at work<br>14. [ ] Police/Soldier<br>15. [ ] Other ___________________

F85.	Do you have any co-wives (does your husband have any other wives)?	1. [ ] YES<br>2. [ ] NO **>>>>>>>>>>>SKIP TO F87**
F86.	In the last twelve months, did your co-wife tell you that she experienced any physical or sexual violence at the hands of your husband?	1. [ ] YES<br>2. [ ] NO<br>3. [ ] I don't know
F87.	Do you have any married sisters?	1. [ ] YES<br>2. [ ] NO **>>>>>>>>>>>SKIP TO F91**
F88.	How many married sisters do you have?	└─┴─┘
F89.	In the last twelve months, did any of your married sisters tell you that she experienced any physical or sexual violence at the hands of their husbands?	1. [ ] YES<br>2. [ ] NO<br>3. [ ] I don't know
F90.	How many of your married sisters experienced physical or sexual violence from their husbands?	└─┴─┘
F91.	Think about the four neighboring houses closest to you.<br>List all of the married or cohabitating women that live in those 4 houses (List names)	1. ____________________<br>2. ____________________<br>3. ____________________<br>4. ____________________<br>5. ____________________<br>6. ____________________
F92.	In the last 12 months, did any of the neighbors you listed experience any physical or sexual violence at the hands of their husbands?	1. [ ] YES<br>2. [ ] NO<br>3. [ ] I don't know
F93.	Of the neighbors you listed, how many experienced any physical or sexual violence at the hands of their husbands in the last 12 months?	└─┴─┘

	These next questions are about your childhood and your family. These questions will ask you about your life when you were growing up and the relationship you had with your parents or the people who cared for you.	
F94	When you were growing up, would you say that your biological mother was never at home, rarely at home or often at home?	1. [ ] Often at home<br>2. [ ] Rarely at home<br>3. [ ] Never at home
F95	When you were growing up, would you say that your biological father was never at home, rarely at home or often at home?	1. [ ] Often at home<br>2. [ ] Rarely at home<br>3. [ ] Never at home

F96	Apart from your biological father, were there other important male figures in your life when you were growing up?	1. [ ] YES<br>2. [ ] NO
F97	Who had the final word in your household about decisions involving you and your brothers and sisters (their schooling, their activities)? Would you say your mother, father or both equally?	1. [ ] Mother<br>2. [ ] Father<br>3. [ ] Both equally<br>4. [ ] Other<br>___________________
F98	Who had the final word about decisions involving how your family spent money on food and clothing? Would you say your mother, father or both equally?	1. [ ] Mother<br>2. [ ] Father<br>3. [ ] Both equally<br>4. [ ] Other<br>___________________
F99	Who had the final word about decisions involving how your family spent money on large investments such as buying a car, a house or a household appliance? Would you say your mother, father or both equally?	1. [ ] Mother<br>2. [ ] Father<br>3. [ ] Both equally<br>4. [ ] Other<br>___________________
F100	According to the law, is a husband who forces his wife to have sex against her will committing a criminal act (That is, the husband can be fined or put in jail)?	1. [ ] YES<br>2. [ ] NO<br>3. [ ] I don't know
F101	Are there any laws in your country about violence against women?	1. [ ] YES<br>2. [ ] NO<br>3. [ ] I don't know
F102	Have you ever heard of any campaigns or activities in your community or workplace that talk about preventing violence against women?	1. [ ] YES<br>2. [ ] NO<br>3. [ ] I don't know
F103	Have you ever seen an advertisement or public service announcement on television about violence against women?	1. [ ] YES<br>2. [ ] NO<br>3. [ ] I don't know
F104	Have you ever participated in an activity (group session, rally, etc.) in your community or workplace on violence against women?	1. [ ] YES<br>2. [ ] NO<br>3. [ ] I don't know
F105	How many of the following statements truthfully describe your life in the last twelve months? Please do not tell me which statements are true, just how many.<br>1. I delivered a new baby.<br>2. I attended a wedding.<br>3. I received some health service at a hospital.<br>4. I purchased new clothing for myself.	|__|__| statements
F106	How many of the following statements truthfully describe your life in the last twelve months? Please do not tell me which	

	statements are true, just how many.<br>1. I delivered a new baby.<br>2. I attended a wedding.<br>3. I received some health service at a hospital.<br>4. I purchased new clothing for myself.<br>5. My husband slapped me or threw something at me that could hurt me.	└─┴─┘ statements

F107. Anonymous picture technique

I would now like to give you a card with photos. On this card are two pictures, but no other message. The first picture is of a happy face, the second is of a sad face.

No matter what you have already told me!! I would like you to put a mark next to the sad picture if your husband has ever committed any act of physical or sexual violence against you. Please put a mark next to the happy face if this has never happened to you.

Once you have marked the card, please fold it over and put it in an envelope and then on this bag, along with many other women's responses from this village. This will ensure that I do not know your answer.

GIVE RESPONDENT CARD AND PEN. DO NOT LOOK AT RESPONSE. ONCE CARD IS FOLDED, ASK REPONDENT TO PUT IT IN AN ENVELOPE AND INTO A BAG THAT ALSO CONTAINS OTHER COMPLETED CARDS. DO NOT RECORD DETAILS OF QUESTIONNAIRE IDENTIFICATION ON CARD.

Section G.
(a) Knowledge about HIV/AIDS

G1.	Now I would like to talk to you about something else. Have you ever heard of an illness called AIDS?	1. [ ] YES<br>2. [ ] NO
G2.	Where did you hear about it?<br><br>Tick all that are mentioned	1. [ ] Media (tv, radio, newspaper)<br>2. [ ] Pamphlet/leaflet/poster<br>3. [ ] Spouse<br>4. [ ] Neighbor/friend<br>5. [ ] Community Conversations program<br>6. [ ] Other program (ie NGO)<br>7. [ ] Health Worker<br>8. [ ] Other:_____________________
G3.	Have you ever discussed HIV/AIDS with anyone?	1. [ ] YES<br>2. [ ] NO **>>>>>>>>>>> SKIP TO QUESTION G5**
G4.	Who have you discussed HIV/AIDS with?	1.____<br>2.____<br>3.____<br>4.____
G5.	Please mention all of the ways you believe a person can get infected with HIV/AIDS.<br><br>***Do NOT read the list, tick off all that are mentioned.***	1. [ ] Sexual intercourse<br>2. [ ] Sharing needles (drug use)<br>3. [ ] Unclean medical equipment<br>4. [ ] Blood transfusions<br>5. [ ] During pregnancy<br>6. [ ] During birth<br>7. [ ] Through breast milk<br>8. [ ] Mosquito/insect bites<br>9. [ ] Contact with blood of infected person<br>10. [ ] Contact with infected person's toothbrush<br>11. [ ] Casual contact with infected person (i.e. sharing food, cup, glass, handshake, hugging, clothes)<br>12. [ ] Other (specify)____ ______________________<br>13. [ ] Has not heard about HIV/AIDS<br>14. [ ] Don't know/Don't remember
G6.	Is there anything a person can do to avoid getting infected with HIV, the virus that causes AIDS?	1. [ ] YES<br>2. [ ] NO **>>>>>>>>>>>SKIP TO QUESTION G8**<br>3. [ ] Don't Know

G7.	Can you tell me all the ways you know of that people can protect themselves from HIV?<br><br>***Tick all that are mentioned.***	1. [ ] Abstinence<br>2. [ ] Being faithful<br>3. [ ] Using condoms<br>4. [ ] Using condoms correctly and consistently<br>5. [ ] Going for VCT before engaging in sex<br>6. [ ] Not sharing sharp objects<br>7. [ ] Avoiding drugs/alcohol/anything which hampers judgment<br>8. [ ] Avoiding bad company<br>9. [ ] Avoiding prostitution<br>10. [ ] Avoiding walking alone at night<br>11. [ ] Avoiding contact between bloody wounds and skin<br>12. [ ] Avoiding circumcision with unsafe tools<br>13. [ ] Avoiding wife-inheritance<br>14. [ ] Avoiding sugar daddies/mummies<br>15. [ ] Avoiding multiple sexual partners<br>16. [ ] Voluntary male circumcision<br>17. [ ] Other (specify___________________________<br>18. [ ] Don't know/ Don't remember
G8.	Do you think that a healthy-looking person can have HIV?	1. [ ] YES<br>2. [ ] NO<br>3. [ ] Don't Know
G9.	Can a pregnant woman infected with HIV transmit the virus to her baby?	1. [ ] YES<br>2. [ ] NO<br>3. [ ] Don't Know
G10.	Can a mother infected with HIV transmit the virus to her newborn baby through breastfeeding?	1. [ ] YES<br>2. [ ] NO<br>3. [ ] Don't Know
G11.	Can a person get the HIV virus by sharing a meal with someone who is infected?	1. [ ] YES<br>2. [ ] NO<br>3. [ ] Don't Know
G12.	Can people protect themselves from getting the HIV virus by avoiding mosquito bites?	1. [ ] YES<br>2. [ ] NO<br>3. [ ] Don't Know

(b) Attitudes about HIV/AIDS

G13.	Do you personally know anyone who has HIV/AIDS or who died from AIDS?	1. [ ] YES<br>2. [ ] NO<br>3. [ ] Don't Know
G14.	If a member of your family got infected with the AIDS virus, would you want it to remain a secret or not?	1. [ ] YES, remain a secret<br>2. [ ] NO<br>3. [ ] Don't Know/not sure/depends
G15.	If a member of your family got infected with the AIDS virus, would you be wiling to care for her or him in your own household?	1. [ ] YES,<br>2. [ ] NO<br>3. [ ] Don't Know/not sure/depends
G16.	Would you buy fresh vegetables from a shopkeeper or vendor if you knew that the person had the AIDS virus?	1. [ ] YES<br>2. [ ] NO<br>3. [ ] Don't Know
G17.	In your opinion, if a female teacher has the AIDS virus but is not sick, should she be allowed to continue teaching in the school?	1. [ ] YES<br>2. [ ] NO<br>3. [ ] Don't Know
G18.	Do you know of a place where people can go to get tested for the AIDS virus?	1. [ ] YES<br>2. [ ] NO<br>3. [ ] Don't Know
G19.	Where is that? Any other place?<br><br>(Tick all that are mentioned)	1. [ ] Hospital<br>2. [ ] Health center<br>3. [ ] Standalone VCT center<br>4. [ ] Family planning clinic<br>5. [ ] Mobile clinic<br>6. [ ] Pharmacy<br>7. [ ] Other
G20.	I don't want to know the results, but have you ever had a blood test for HIV?	1. [ ] YES<br>2. [ ] NO **>>>>>>>>>>SKIP TO G24**
G21.	How many times have you had a blood test for HIV?	|__|__| times
G22.	When was the last time you had a blood test for HIV/AIDS?	|__|__| month |__|__| year<br>If the month not known enter 01
G23.	I don't want to know the results, but did you get the results of the test?	1. [ ] YES<br>2. [ ] NO
G24.	Do you know a place where you can get condoms?	1. [ ] YES<br>2. [ ] NO **>>>>>>>>>>SKIP TO G26**

G25.	Where can you get condoms?<br><br>(tick all that are mentioned)	1. [ ] Hospital<br>2. [ ] Health center/clinic<br>3. [ ] Mobile clinic<br>4. [ ] Community health worker<br>5. [ ] Pharmacy<br>6. [ ] Street vendor<br>7. [ ] Bars/clubs<br>8. [ ] Shop<br>9. [ ] Friends/relative<br>10 [ ] Church<br>11. [ ] NGO<br>12. [ ] Other (specify)_________

FO: The objective of the next section is to find out your opinions about HIV/AIDS. I will read a statement. Please tell me if you strongly agree/agree/neither agree nor disagree/ disagree or strongly disagree with each statement.

Statements		Answer					
		Strongly Agree	Agree	Neither Agree nor Disagree	Disagree	Strongly Disagree	Don't know
G26.	Using condoms reduces sexual pleasure.	1	2	3	4	5	0
G27.	It is embarrassing to buy or ask for condoms.	1	2	3	4	5	
G28.	Using a condom is a sign of not trusting your partner.	1	2	3	4	5	
G29.	Men need to have more than one sexual partner, often at the same time.	1	2	3	4	5	
G30.	HIV/AIDS is a punishment for bad behavior.	1	2	3	4	5	
G31.	It is women prostitutes that spread HIV in our community.	1	2	3	4	5	
G32.	It is promiscuous men that spread HIV in our community.	1	2	3	4	5	

(c) Behavior Questions

FO: Now I am going to ask you some more questions. These ones will be about some topics that may seem embarrassing. Again, this is totally confidential and I won't share this information anyone else.

G33.	How old were you when you had sexual intercourse for the first time?	|__|__| years
G34.	In your **lifetime**, how many sexual partners have you had? Including the current spouse	|__|__| partners
G35.	In the **last 6 months**, how many sexual partners have you had? Including the current spouse	|__|__| partners

G36.	Have you **ever** used a condom?	1. [ ] YES<br>2. [ ] NO **>>>>>>>> skip to question G40**
G37.	Did you use a condom **last time you had sex**?	1. [ ] YES<br>2. [ ] NO **>>>>>>>> skip to question G39**<br>3. [ ] Not sure
G38.	What were the reasons you used a condom?<br>*(tick all that are mentioned)*	1. [ ] Own concern to prevent pregnancy<br>2. [ ] Own concern to prevent STI/HIV<br>3. [ ] Partner insisted/ partner's choice<br>4. [ ] Other (specify)<br>If any of the above answered**>> skip to G40**
G39.	What were the reasons you did not use a condom?<br>*(tick all that are mentioned)*	1. [ ] Condoms are uncomfortable<br>2. [ ] Embarrassing to use condoms<br>3. [ ] Unable to get condoms<br>4. [ ] Had sex with a partner who was not a sex worker<br>5. [ ] Had sex with my regular partner<br>6. [ ] Partner did not want<br>7. [ ] Other (specify)_______________
G40.	Would you like to use a condom next time you have sex?	1. [ ] YES<br>2. [ ] NO<br>3. [ ] I Don't Know
G41.	Who will make the decision about whether to use a condom?	1. [ ] Self<br>2. [ ] Partner<br>3. [ ] Both<br>4. [ ] Not sure
G42.	How confident are you that you could convince your partner to use a condom if you wanted to use one?	1. [ ] Not at all confident<br>2. [ ] Somewhat confident<br>3. [ ] Confident<br>4. [ ] Very confident
G43.	What would you do if your partner refused or declined to use a condom?	1. [ ] Have sex without condom<br>2. [ ] Talk it over and use condom<br>3. [ ] Talk it over and not use condom<br>4. [ ] Would not have sex<br>5. [ ] Other______________________<br>6. [ ] Don't Know
G44.	Could you say no to your partner if you did not want to have sexual intercourse?	1. [ ] Yes<br>2. [ ] No<br>3. [ ] Depends/Not sure
G45.	Have you ever discussed HIV risk behaviors with your partner?	1. [ ] YES<br>2. [ ] NO

G46.	Now I would like to ask you some questions about your health in the last 12 months. During the last 12 months, have you had a disease which you got through sexual contact?	1. [ ] YES<br>2. [ ] NO<br>3. [ ] Don't Know
G47.	Sometimes women experience a bad smelling abnormal genital discharge. During the last 12 months, have you had a bad smelling abnormal genital discharge?	1. [ ] YES<br>2. [ ] NO<br>3. [ ] Don't Know
G48.	Sometimes women have a genital sore or ulcer. During the past 12 months, have you had a genital sore or ulcer?	1. [ ] YES<br>2. [ ] NO<br>3. [ ] Don't Know
G48b	*Enumerator Check: Did the respondent answer yes to question either G46, G47, OR D48?*	1. [ ] YES<br>2. [ ] **NO >>>>>>>>SKIP TO G51**
G49.	The last time you had (PROBLEM FROM G46/G47/G48) did you seek any kind of advice or treatment?	1. [ ] YES<br>2. [ ] NO<br>3. [ ] Don't Know
G50.	Where did you go?	1. [ ] Community health worker<br>2. [ ] Health post<br>3. [ ] Primary health center<br>4. [ ] Government hospital<br>5. [ ] Private hospital<br>6. [ ] Private drug store<br>7. [ ] Other (specify)
G51.	Have you ever heard of female circumcision?<br>IF NO PROBE: Have you ever heard of the practice in which a girl may have parts of her genitals cut	1. [ ] YES<br>2. [ ] NO
G52.	Have you yourself ever been circumcised?	1. [ ] YES<br>2. [ ] NO<br>3. [ ] Don't Know
G53.	In some parts of Ethiopia, there is a type of circumcision where the genital area is sewn closed. Was this done to you?	1. [ ] YES<br>2. [ ] NO<br>3. [ ] Don't Know
G54.	*ENUMERATOR CHECK: Does the respondent have at least one living daughter (Question D2)*	1. [ ] YES<br>2. [ ] NO >>>>>>>**SKIP TO G56**
G55.	Has your daughter/ have any of your daughters been circumcised?<br><br>IF YES: How many?	1. [ ] YES<br>2. [ ] NO<br><br>|__|__| daughters
G56.	Do you think this practice should be continued or should it be discontinued?	1. [ ] Continued<br>2. [ ] Discontinued<br>3. [ ] Depends<br>4. [ ] Don't know

H. <u>Patient Health Questionnaire-9</u>

Over the last 2 weeks, how often have you been bothered by any of the following problems?		Answer			
		Not at all	Several Das	More than half the days	Nearly every day
H1.	Little interest or pleasure in doing things	0	1	2	3
H2.	Feeling down, depressed or hopeless	0	1	2	3
H3.	Trouble falling or staying asleep, or sleeping too much	0	1	2	3
H4.	Feeling tired or having little energy	0	1	2	3
H5.	Poor appetite or overeating	0	1	2	3
H6.	Feeling bad about yourself- or that you are a failure or have let yourself or your family down	0	1	2	3
H7.	Trouble concentrating on things, such as reading the newspaper or watching television	0	1	2	3
H8.	Moving or speaking so slowly that other people could have noticed. Or the opposite – being so figety or restless that you have been moving around a lot more than usual	0	1	2	3
H9.	Thoughts that you would be better off dead, or of hurting yourself	0	1	2	3
		Not difficult at all	Somewh at difficult	Very difficult	Extremel y difficult
H10.	If you checked off any problems, how difficult have these problems made it for you to do your work, take care of things at home, or get along with other people?	0	1	2	3

The End

Note:

Before leaving the participant

- **Check for consistency and completeness of each question**
- **Provide the referral and community resource after informing her**
- **Provide greetings and thank her.**
