# Supplementary material for: Effectiveness of a culturally appropriate intervention to prevent intimate partner violence and HIV transmission among men, women, and couples in rural Ethiopia: Findings from a cluster-randomized controlled trial
Source: PLoS Med. 2020 Aug 18;17(8):e1003274. doi: 10.1371/journal.pmed.1003274 (PMC7433859; doi:10.1371/journal.pmed.1003274)
Supplement: S4 Text — (PDF) [file pmed.1003274.s007.pdf]

Women's Health and Relationships Project in Ethiopia.

Collaborative Study by

The School of Public Health, Addis Ababa University;

The Ethiopian Public Health Association

and

The Abdul Latif Jameel Poverty Action Lab

Men's Questionnaire
የወንዶች መጠይቅ

Addis Ababa, Ethiopia
Nov 2014

Eligibility of study subject

1. A man in the age between 18-49 years
2. Currently living in a marriage or cohabiting with a woman
3. Residing in the selected village for at least 6 months

BASELINE SURVEY

Identity Number: |__|__|__| |__|__|__| |__|__|__|
Woreda Gotte House No

Enumerator Information

1.	Names and Codes of enumerators	____________________ |__|__|__|
2.	Name and codes of the supervisors	____________________ |__|__|__|

Identification

3.	District (Woreda)	W01 Meskan<br>W02 Mareko<br>W03 Silte<br>W04 Sodo	|__|__|__|
4.	Village (Kebele), name and code		|__|__|__|
5.	Subvillage (Gote), name and code		|__|__|__|
6.	Name of respondent	|________|________|________| Name Father's Name Grand name	
7.	Name of spouse	|________|________|________| Name Father's Name Grand name	
8.	Address (write down names of road, alley, house number) Given by supervisor (computer identity)	|__|__|__|	
9.	Identity Number	|__|__|__| |__|__|__| |__|__|__| Woreda Gotte House No	
10.	Telephone/Cellphone<br>Self…. Spouse .Family… Neighbor..<br>If no phone number put 000000	|__|__|__|__|__|__|__|__|__|__| 1. Self 2. Spouse 3 Family 4.Neighbor 5. None	

Results of Visits

11.		First Visit	Second Visit	Third Visit
12.	Date	|__|__| / |__|__| / 20|__|__| Date Month Year	|__|__| / |__|__| / 20|__|__| Date Month Year	|__|__| / |__|__| / 20|__|__| Date Month Year
13.	Time Interview Started	|__|__| : |__|__| Hour Minute	|__|__| : |__|__| Hour Minute	|__|__| : |__|__| Hour Minute
14.	Time interview completed	|__|__| : |__|__| Hour Minute	|__|__| : |__|__| Hour Minute	|__|__| : |__|__| Hour Minute
15.	Results of Visits	1. [ ] Completed<br>2. [ ] Partly Completed<br>3. [ ] Respondent not at home<br>4. [ ] Respondent Declined<br>5. [ ] Respondent not available in the village<br>6. [ ] Other (Specify)_____<br>Make appointment if 2 or 3 answered	1. [ ] Completed<br>2. [ ] Partly Completed<br>3. [ ] Respondent not at home<br>4. [ ] Respondent Declined<br>5. [ ] Respondent not available in the village<br>6. [ ] Other (Specify)_____<br>1. Make appointment if 2 or 3 answered	1. [ ] Completed<br>2. [ ] Partly Completed<br>3. [ ] Respondent not at home<br>4. [ ] Respondent Declined<br>5. [ ] Respondent not available in the village<br>6. [ ] Other (Specify)_____

A. Background Info/Education/Community Participation/SES

A1.	In what day, month and year were you born?<br>If he does not know his date of birth – write 01/01/year	└─┴─┘ / └─┴─┘ / └─┴─┴─┴─┘<br>Day month Year
A2.	How old were you at your last birthday?	└─┴─┘ Years
A3.	Are you able to read?	1. [ ] Yes<br>2. [ ] No **>>>>>SKIP TO A8**
A4.	Have you ever attended school?	1. [ ] Yes<br>2. [ ] No **>>>SKIP to A7**
A5.	What is the highest level of school you attended-primary, secondary or higher?	1. [ ] Primary (grade 1-8)<br>2. [ ] Secondary (grade 9-12)<br>3. [ ] Higher (>=13)
A6.	What is the highest class/form/year you completed at that level?	└─┴─┘
A7.	How often do you read a newspaper or magazine?	1. [ ] Almost every day<br>2. [ ] At least once a week<br>3. [ ] Less than once a week<br>4. [ ] Not at all
A8.	How often do you listen to the radio almost every day?	1. [ ] Almost every day<br>2. [ ] At least once a week<br>3. [ ] Less than once a week<br>4. [ ] Not at all
A9.	How often do you watch television?	1. [ ] Almost every day<br>2. [ ] At least once a week<br>3. [ ] Less than once a week<br>4. [ ] Not at all
A10.	What is your religion?	1. [ ] Muslim<br>2. [ ] Orthodox<br>3. [ ] Protestant<br>4. [ ] Catholic<br>5. [ ] Other _______________
A11.	How many years have you lived in this village?	└─┴─┘ year └─┴─┘ Month
A12.	How was your marriage ceremony conducted?	1.[ ] Municipality Wedding<br>2.[ ] Religious ceremony<br>3.[ ] By elderly people<br>4.[ ] Cohabiting (non-certified)
A13.	How many wives do you have?	└─┴─┘ wives
A14.	If currently married or cohabiting, how long have you been living with your spouse or sexual partner? You may answer in months or years, but not both.	└─┴─┘ months └─┴─┘ Years
A15.	How many times have you been married (civil, religious, common, customary law marriage)?	└─┴─┘ times<br>**IF 00 SKIP TO A18**

A16.	How old were you when you first got married?	└─┴─┘ Years old
A17.	The building that you sleep in – of what material are the walls made?<br><br>*(Tick all that apply;<br>o be filled by observation)*	1. [ ] Stone, solid cement<br>2. [ ] Fired brick<br>3. [ ] Sun-dried bricks<br>4. [ ] Mud, cement<br>5. [ ] Mud, sticks, reeds<br>6. [ ] Other (specify)______________
A18.	What is the roof of this building made of?<br>*(Tick all that apply;<br>to be filled by observation)*	1. [ ] Iron<br>2. [ ] Grass or reeds<br>3. [ ] Mud, branches<br>4. [ ] Mud, cement<br>5. [ ] Other (specify)______________
A19.	What is the floor of your house made of?<br>*(Tick all that apply; to be filled by observation)*	1. [ ] Cement<br>2. [ ] Mud<br>3. [ ] Cow dung/soil<br>4. [ ] Other (specify) ______________
A20.	Do you own a latrine?	1. [ ] Yes, outdoor pit latrine, shared<br>2. [ ] Yes, outdoor pit latrine, private<br>3. [ ] Yes, indoor latrine<br>4. [ ] Yes, outdoor pit latrine and indoor latrine<br>5. [ ] No, neither type of latrine **>>>skip to A22**
A21.	If you have a latrine at home, do you use the latrine nearly always, for long call only, or rarely?	1. [ ] Use it always<br>2. [ ] Use it for long call only<br>3. [ ] Use it rarely
A22.	Does your household have:<br><br>a. Electricity?<br>b. Watch/clock?<br>c. A radio?<br>d. A television?<br>e. A mobile phone?<br>f. An ox?<br>g. A cow?<br>h. A table?<br>i. A chair?<br>j. A bed?<br>k. A cotton/sponge/spring mattress?	 <br>a. 1. [ ] Yes 2. [ ] No<br>b. 1. [ ] Yes 2. [ ] No<br>c. 1. [ ] Yes 2. [ ] No<br>d. 1. [ ] Yes 2. [ ] No<br>e. 1. [ ] Yes 2. [ ] No<br>f. 1. [ ] Yes 2. [ ] No<br>g. 1. [ ] Yes 2. [ ] No<br>h. 1. [ ] Yes 2. [ ] No<br>i. 1. [ ] Yes 2. [ ] No<br>j. 1. [ ] Yes 2. [ ] No<br>k. 1. [ ] Yes 2. [ ] No
A23.	If one walked at an average pace, approximately how many minutes would it take to walk from your home to the nearest place where you can catch a bus?	|__|__|__| minutes

B. Wife's background information, Employment status, and man's economic and Employment status

B1.	How old was your wife/ main partner on her last birthday? Compute with your age	└─┴─┘ Years
B2.	Did your Wife/ main partner ever attend school?	1. [ ] Yes<br>2. [ ] No **>>>>>>>>>skip to B5**
B3.	What was the highest level of school she attended: primary, secondary or higher?	1. [ ] Primary(grade 1-8)<br>2. [ ] Secondary(grade 9-12)<br>3. [ ] Higher(>=13)
B4.	What was the highest grade/form/year that she completed at that level?	└─┴─┘
B5.	What is your wife's/ main partner's occupation? That is what kind of work does she mainly do?	1. [ ] Farming<br>2. [ ] Trade<br>3. [ ] Employed (Gov/ private)<br>4. [ ] Daily laborer<br>5. [ ] Other______________
B6.	Does this household own any agricultural land?	1. [ ] Yes<br>2. [ ] No **>>>>>>>>>skip to B9**
B7.	How much agricultural land does the household own? (1 Timmad is 0.25 Hectare)	|__|__| **.** |__|__| Timmad
B8.	What crop do you mainly grow?	1. [ ] Maize<br>2. [ ] Barely<br>3. [ ] Pepper<br>4. [ ] Teff<br>5. [ ] False banana<br>6. [ ] Bean<br>7. [ ] Khat<br>8. [ ] Coffee<br>9. [ ] Other______________
B9.	What is your average monthly income?	|__|__|__|__| Birr
B10.	Have you ever worked or done any activities for income?	1. [ ] Yes<br>2. [ ] No **>>>>>SKIP TO B18**
B11.	What kind of work or activities to earn income have you done in the last 12 months?	__________________
B12.	Do you do this work for a member of your family, for someone else, or are you self-employed? Read the response	1. [ ] For family member<br>2. [ ] For himself<br>3. [ ] For other person
B13.	Do you usually work throughout the year, or do you work seasonally, or only once in a while? Clarify	1. [ ] Throughout the year<br>2. [ ] Seasonally<br>3. [ ] Once in a while
B14.	Are you paid in cash or kind for this work or are you not paid at all?<br>Clarify	1. [ ] Cash only<br>2. [ ] In kind only<br>3. [ ] Cash and Kind

B15.	What was your average daily wage?<br>If in kind paid, change to a monetary	|__|__|__|Birr
B16.	Who usually decides how the money you earn will be used: mainly you, mainly your wife/ partner or you and your wife/partner jointly?	1. [ ] Respondent<br>2. [ ] Wife/partner<br>3. [ ] Respondent and wife/ partner jointly<br>4. [ ] Other______________
B17.	Would you say that the money that you earn is more than what your wife/partner earns, less than what she earns or about the same?	1. [ ] More than her<br>2. [ ] Less than her<br>3. [ ] About the same<br>4. [ ] wife/partner doesn't bring in money<br>5. [ ] Don't know
B18.	Who usually decides how your wife's/ partner's earnings will be used: you, your wife/partner, or you and your wife/partner jointly?	1. [ ] Respondent<br>2. [ ] wife/ partner<br>3. [ ] Respondent and wife/partner jointly<br>4. [ ] Other______________
B19.	How many times per week or per day you participate in a traditional coffee ceremony?<br>If answered per week, enter 00 on the day<br>If answered per day, enter 00 on the weeks	|__|__| per day |__|__| per week
B20.	Who usually participates in the coffee ceremony with you?<br>Tick all that apply	1. [ ] wife/partner<br>2. [ ] Family members<br>3. [ ] Neighbors<br>4. [ ] Village leader<br>5. [ ] Religious leader<br>6. [ ] Friends<br>7. [ ] Other______________
B21.	What do you discuss with others during a coffee ceremony?<br>Tick all that apply	1. [ ] Family or personal issues<br>2. [ ] Issues related to your community<br>3. [ ] National or political news<br>4. [ ] Health challenges<br>5. [ ] Problems with farming or cattle<br>6. [ ] Other
B22.	Who usually prepares the coffee during the traditional coffee ceremony?	1. [ ] Men<br>2. [ ] Women
B23.	Have you ever prepared the coffee during the traditional coffee ceremony?	1. [ ] Yes<br>2. [ ] No
B24.	Would you be happy to participate in a coffee ceremony if a man prepared the coffee?	1. [ ] Yes<br>2. [ ] No**>>>>>SKIP TO B26**
B25.	If happy, Why?	1. [ ] Men and women are equal.<br>2. [ ] Both men and women can participate fully in the coffee ceremony.<br>3. [ ] I enjoy participating without having to make coffee.<br>4. [ ] Other: ________________
B26.	Why not?	1. [ ] This is not customary<br>2. [ ] It is better if women prepare the coffee

		ceremony.<br>3. [ ] Men should not perform women's role in a coffee ceremony.<br>4. [ ] Other: _________________

B27.	Now if you permit me, I will ask some more questions about your relationship with your wife. In the last twelve months, did you ever:	Yes	No	If Yes, how often?		
				Often	Sometimes	Rarely
	a) Expect that your wife ask permission to purchase large items?	1	2	1	2	3
	b) Expect that your wife ask permission to purchase small items?	1	2	1	2	3
	c) Taken her earnings or savings from her against your will?	1	2	1	2	3
	d) Refuse to give her money for household expenses, even when you had money for other things?	1	2	1	2	3
	e) Require that she give up or refuse a job for money outside the home because you did not want your wife to work?	1	2	1	2	3
	f) Make important financial decisions without talking to your wife about them?	1	2	1	2	3
	g) Decide how she could spend money	1	2	1	2	3
	h) Demand to know how she spent money?	1	2	1	2	3
	i) Hide money from her?	1	2	1	2	3
	j) Spent money she needed for rent, food or clothes on things for yourself?	1	2	1	2	3
	k) Take things from her that were hers?	1	2	1	2	3

	l) Threaten to not give her money or take it away from her	1	2	1	2	3
	m) Given her little money or reduced her spacing when you were angry?	1	2	1	2	3
	n) Stopped giving her money for food?	1	2	1	2	3
	o) Taken away or used her belongings against her will?	1	2	1	2	3

B28.	How often do you drink alcohol?<br><br>1. Every day or nearly every day<br>2. Once or twice a week<br>3. 1-3 times a month<br>4. Occasionally, less than once a month<br>5. Never	1. [ ] Every day or nearly every day<br>2. [ ] Once or twice a week<br>3. [ ] 1-3 times in a month<br>4. [ ] Very Rarely<br>5. [ ] Never **>>>>SKIP TO B31**
B29.	In the past 12 months how often have you drunk?	1. [ ] Most days<br>2. [ ] Weekly<br>3. [ ] Once a month<br>4. [ ] Very Rarely<br>5. [ ] Never drinks >>> Skip to B31
B30.	In the past 12 months, have you experienced any of the following problems, related to your drinking?<br><br>a. Money problems<br>b. Health problems<br>c. Conflict with family or friends<br>d. Problems with authorities (police etc)<br><br>e. Any other problems (specify)	a. 1. [ ] Yes 2. [ ] No<br>b. 1. [ ] Yes 2. [ ] No<br>c. 1. [ ] Yes 2. [ ] No<br>d. 1. [ ] Yes 2. [ ] No<br><br>e. Other(Specify)_______________
B31.	How often do you chew khat?<br><br>1. Every day or nearly every day<br>2. Once or twice a week<br>3. 1-3 times a month<br>4. Occasionally, less than once a month<br>5. Never<br>6. Don't Know	1. [ ] Every day or nearly every day<br>2. [ ] Once or twice a week<br>3. [ ] 1-3 times in a month<br>4. [ ] Less than once a month<br>5. [ ] Never >>> Skip to C1<br>6. [ ] Don't know
B32.	In the past 12 months, have you experienced any of the following problems, related to your khat chewing?<br><br>a. Money problems<br>b. Health problems<br>c. Conflict with family or friends	a. 1. [ ] Yes 2. [ ] No<br>b. 1. [ ] Yes 2. [ ] No<br>c. 1. [ ] Yes 2. [ ] No

	d. Problems with authorities (police etc)<br><br>e. Any other problems (specify)	d. 1. [ ] Yes 2. [ ] No<br><br>e. Other(Specify)______________

C. Fatherhood

We know some of those were difficult questions to answer. Thank you for doing so, your answers are really important. The next questions are about you and the children you may have fathered or adopted or children who may live with you even if they are not legally or biologically yours.

C1.	How many biological children do you have?<br>If no child enter 00	└┴┘<br>If no child, skip to C6
C2.	How old is your youngest biological child?<br>If the child died, fill near the age he/ she died 99	└┴┘ Years<br>└┴┘ months (if age below 1 year, enter on months)<br>└┴┘ days (if age below 1 month, enter on days)
C3.	How old is your eldest biological child?<br>If the child died, fill near the age he/ she died 99	└┴┘ Years<br>└┴┘ months (if age below 1 year, enter on months)<br>└┴┘ days (if age below 1 month, enter on days)
C4.	Were you present at the birth of your last child?	1. [ ] Yes**>>>>> Skip to C7**<br>2. [ ] No
C5.	Did you choose not to be present at the birth or did you want to be present but were prevented from doing so for some reason?	1. [ ] Wanted to be present<br>2. [ ] Didn't want to be present
C6.	Did you accompany the mother of your child to any prenatal visits during the last pregnancy?	1. [ ] Yes<br>2. [ ] No<br>3. [ ] She didn't have prenatal visits<br>4. [ ] She has never been pregnant

F. Gender and Domestic Violence

Enumerator: Now, I am going to read you a list of statements. For each statement, I would like you to first tell me if you agree with this statement yourself, and next tell me if you think a majority (more than half) of your friends and family agree with this statement. For example, I could read the statement "It is beneficial for all children to attend primary school." First, you would say "Yes" if you agree with this statement yourself. Next, if you believe that the majority of your friends and families also agree, you would say "Yes" again.

		(a) Do you agree with this statement?	(b) Do your friends and family agree with this statement?
F1.	A man should have the final word on decisions in his home.	1. [ ]Yes<br>2. [ ]No	1. [ ]Yes<br>2. [ ]No
F2.	A woman should obey her husband in all things.	1. [ ]Yes<br>2. [ ]No	1. [ ]Yes<br>2. [ ]No
F3.	It must be the man who provides for the family.	1. [ ]Yes<br>2. [ ]No	1. [ ]Yes<br>2. [ ]No
F4.	The husband should decide what major household items to buy	1. [ ]Yes<br>2. [ ]No	1. [ ]Yes<br>2. [ ]No
F5.	If a wife does something wrong her husband has the right to punish her.	1. [ ]Yes<br>2. [ ]No	1. [ ]Yes<br>2. [ ]No
F6.	A woman should be able to choose her own friends even if her husband disapproves	1. [ ]Yes<br>2. [ ]No	1. [ ]Yes<br>2. [ ]No
F7.	A woman should be able to get help from a skilled birth attendant when she needs it, even without her husband's approval.	1. [ ]Yes<br>2. [ ]No	1. [ ]Yes<br>2. [ ]No
F8.	A husband and wife should make decisions together about health care for themselves.	1. [ ]Yes<br>2. [ ]No	1. [ ]Yes<br>2. [ ]No
F9.	A husband and wife should make decisions together about health care for their children.	1. [ ]Yes<br>2. [ ]No	1. [ ]Yes<br>2. [ ]No
F10.	Village assemblies and local development committee members should be mainly men because women don't have a voice in village matters.	1. [ ]Yes<br>2. [ ]No	1. [ ]Yes<br>2. [ ]No

	Continues	(a) Do you agree with this statement?	(b) Do your friends and family agree with this statement?
F11.	A man needs other women, even if things with his wife are fine.	1. [ ]Yes<br>2. [ ]No	1. [ ]Yes<br>2. [ ]No
F12.	You don't talk about sex, you just do it.	1. [ ]Yes<br>2. [ ]No	1. [ ]Yes<br>2. [ ]No
F13.	Women who carry condoms on them are easy.	1. [ ]Yes<br>2. [ ]No	1. [ ]Yes<br>2. [ ]No
F14.	Men need sex more than women do.	1. [ ]Yes<br>2. [ ]No	1. [ ]Yes<br>2. [ ]No
F15.	Men are always ready to have sex.	1. [ ]Yes<br>2. [ ]No	1. [ ]Yes<br>2. [ ]No
F16.	A woman should not initiate sex.	1. [ ]Yes<br>2. [ ]No	1. [ ]Yes<br>2. [ ]No
F17.	It is the man who decides what type of sex to have.	1. [ ]Yes<br>2. [ ]No	1. [ ]Yes<br>2. [ ]No
F18.	A man should be outraged if his wife asks him to use a condom.	1. [ ]Yes<br>2. [ ]No	1. [ ]Yes<br>2. [ ]No
F19.	A woman can suggest using condoms just like a man can.	1. [ ]Yes<br>2. [ ]No	1. [ ]Yes<br>2. [ ]No
F20.	A married woman should not refuse to have sex if her husband wants to.	1. [ ]Yes<br>2. [ ]No	1. [ ]Yes<br>2. [ ]No
F21.	It is a woman's responsibility to avoid getting pregnant.	1. [ ]Yes<br>2. [ ]No	1. [ ]Yes<br>2. [ ]No
F22.	A couple should decide together if they want to have children.	1. [ ]Yes<br>2. [ ]No	1. [ ]Yes<br>2. [ ]No
F23.	If a man gets a woman pregnant, the child is the responsibility of both.	1. [ ]Yes<br>2. [ ]No	1. [ ]Yes<br>2. [ ]No
F24.	A woman who has sex before she marries does not deserve respect.	1. [ ]Yes<br>2. [ ]No	1. [ ]Yes<br>2. [ ]No
F25.	Only when a woman has a child is she a real woman.	1. [ ]Yes<br>2. [ ]No	1. [ ]Yes<br>2. [ ]No
F26.	A real man produces a male child.	1. [ ]Yes<br>2. [ ]No	1. [ ]Yes<br>2. [ ]No
F27.	Parents should be happy if their daughter is going to be married at any age – even if she is younger than 18 years old.	1. [ ]Yes<br>2. [ ]No	1. [ ]Yes<br>2. [ ]No
F28.	Women are sometimes to blame for violence against them.	1. [ ]Yes<br>2. [ ]No	1. [ ]Yes<br>2. [ ]No

	**Continues**	(a) Do you agree with this statement?	(b) Do your friends and family agree with this statement?
F29.	Women should not tolerate violence just in order to keep her family together.	1. [ ]Yes<br>2. [ ]No	1. [ ]Yes<br>2. [ ]No
F30.	No one outside the couple should intervene if a husband beats up his wife.	1. [ ]Yes<br>2. [ ]No	1. [ ]Yes<br>2. [ ]No
F31.	There are times a woman deserves to be beaten.	1. [ ]Yes<br>2. [ ]No	1. [ ]Yes<br>2. [ ]No
F32.	A woman who intervenes when a husband is beating his wife is meddling.	1. [ ]Yes<br>2. [ ]No	1. [ ]Yes<br>2. [ ]No

FO: Now, some people may believe that a man has a good reason to hit his wife in certain situations. I am going to read you a list of situations. For each one, please tell me if you believe that a man has a good reason to hit his wife in this situation. Please also tell me if you think most of your friends and families agree that a man would have a good reason to hit his wife in this situation.

		(a) Do you agree a man would have a good reason to hit his wife in this situation?	(b) Would your friends and family agree a man has a good reason to hit his wife in this situation?
F33.	She disobeys him.	1. [ ]Yes<br>2. [ ]No	1. [ ]Yes<br>2. [ ]No
F34.	She answers back to him.	1. [ ]Yes<br>2. [ ]No	1. [ ]Yes<br>2. [ ]No
F35.	She disrespects his relatives.	1. [ ]Yes<br>2. [ ]No	1. [ ]Yes<br>2. [ ]No
F36.	He suspects that she is unfaithful.	1. [ ]Yes<br>2. [ ]No	1. [ ]Yes<br>2. [ ]No
F37.	He finds out that she has been unfaithful.	1. [ ]Yes<br>2. [ ]No	1. [ ]Yes<br>2. [ ]No
F38.	She spends her time gossiping with neighbors.	1. [ ]Yes<br>2. [ ]No	1. [ ]Yes<br>2. [ ]No
F39.	She neglects taking care of the children.	1. [ ]Yes<br>2. [ ]No	1. [ ]Yes<br>2. [ ]No
F40.	She does not complete her household work to his satisfaction.	1. [ ]Yes<br>2. [ ]No	1. [ ]Yes<br>2. [ ]No
F41.	She accuses him of infidelity.	1. [ ]Yes<br>2. [ ]No	1. [ ]Yes<br>2. [ ]No
F42.	She tells his secrets to others in the community.	1. [ ]Yes<br>2. [ ]No	1. [ ]Yes<br>2. [ ]No
F43.	He is angry with her.	1. [ ]Yes<br>2. [ ]No	1. [ ]Yes<br>2. [ ]No
F44.	She burns the food.	1. [ ]Yes	1. [ ]Yes

		2. [ ]No	2. [ ]No
F45.	She goes out without telling him.	1. [ ]Yes<br>2. [ ]No	1. [ ]Yes<br>2. [ ]No

FO: Now I would like to ask for your opinion on some other important aspects of a woman's life. I know some of these questions are very personal. However, your answers are crucial for helping to understand the condition of women in Ethiopia. Let me assure you that your answers are completely confidential and will not be told to anyone and no one else will know that you were asked these questions.

F46.	Do you think violence against women is a major problem in your village?	1. [ ] YES<br>2. [ ] NO<br>3. [ ] Don't Know
F47.	Out of 10 women living in your village, how many do you think are experiencing any type of physical violence from their husbands?	└─┴─┘ women
F48.	Out of 10 women living in your village, how many do you think are experiencing any type of sexual violence from their husbands?	└─┴─┘ women
F49.	What are some of the consequences of violence against women?<br>Tick all that are mentioned.<br>(**Do not read options**)	1. [ ] Psychological outcomes<br>2. [ ] Physical injuries<br>3. [ ] Sexual and reproductive health problems<br>4. [ ] Suicide or death<br>5. [ ] Health or psychological risks for children<br>6. [ ] HIV/ AIDS or STD transmission<br>7. [ ] Other (specify) ___________________<br>8. [ ] Don't know
F50.	How does violence against women increase their vulnerability to HIV/AIDS?<br>Tick all that are mentioned.<br>(**Do not read options**)	1. [ ] Limits ability to negotiate safe sex<br>2. [ ] Violent men may engage in risky sexual behaviors and put their spouses at risk<br>3. [ ] Limits access to information and treatment<br>4. [ ] Increases risk of transmission to infants<br>5. [ ] other specify ______________________
F51.	If a woman in your village was experiencing violence from her husband, who could she go to for help?<br>(**Do not read options**	1. [ ] Female relative/friend<br>2. [ ] Male relative<br>3. [ ] Police officer<br>4. [ ] Health worker<br>5. [ ] Religious leader<br>6. [ ] Community leader<br>7. [ ] Neighbor<br>8. [ ] Other: ________________________

F52.	What would you do if you saw a male friend use physical or sexual violence against a woman?<br><br>Tick all that are mentioned.<br>(**Do not read options**)	1. [ ] Intervene during the episode<br>2. [ ] Speak to him after the episode<br>3. [ ] Avoid/shun him<br>4. [ ] Call the police<br>5. [ ] Do nothing, as it is their problem<br>6. [ ] Mobilize neighbors or friends<br>7. [ ] Contact village elders<br>8. [ ] Other
F53.	What would you do if you saw physical or sexual violence being carried out by a stranger (man) against a woman?<br><br>Tick all that are mentioned.<br>(**Do not read options**)	1. [ ] Intervene during the episode<br>2. [ ] Speak to him after the episode<br>3. [ ] Avoid/shun him<br>4. [ ] Call the police<br>5. [ ] Do nothing, as it is their problem<br>6. [ ] Mobilize neighbors or friends<br>7. [ ] Contact village elders<br>8. [ ] Other
F54.	I am going to read some statements about violence against women. For each statement, please state whether you agree or disagree.<br><br>a) If a man does not give his wife money for household necessities, even though he spends money on things for himself, it is an example of violence against women.<br><br>b) If a man forbids his wife from seeing his parents and other family members, or friends, it is not an example of violence against women.<br><br>c) If a man slaps, pushes, shoves, kicks or drags a woman, including his wife, it is not an example of violence against women.<br><br>d) If a man threatens to hit a woman, but does not actually hit her, it is an example of violence against women.<br><br>e) If a man forces a woman he does not know to have sex, it is an example of violence against women.<br><br>f) If a man forces his wife to have sex, it is not an example of violence against women.<br><br>g) If a man shouts at his wife in public and embarrasses her, it is an example of violence against women.	a) 1. [ ] Agree 2. [ ] Disagree<br><br>b) 1. [ ] Agree 2. [ ] Disagree<br><br>c) 1. [ ] Agree 2. [ ] Disagree<br><br>d) 1. [ ] Agree 2. [ ] Disagree<br><br>e) 1. [ ] Agree 2. [ ] Disagree<br><br>f) 1. [ ] Agree 2. [ ] Disagree<br><br>g) 1. [ ] Agree 2. [ ] Disagree

F55.	**When two people marry or live together, they usually share both good and bad moments. I would now like to ask you some questions about your current and past relationships and how you treat your wife. If anyone interrupts us I will change the topic of conversation. I would again like to assure you that your answers will be kept secret, and that you do not have to answer any questions that you do not want to. May I continue?**		
F56.	In general, do you and your wife/partner discuss the following topics together:<br><br>a) Things that have happened to you in the day<br>b) Things that happened to her during the day<br>c) Your worries or feelings<br>d) Her worries or feelings	a) 1. [ ] Yes 2. [ ] No<br>b) 1. [ ] Yes 2. [ ] No<br>c) 1. [ ] Yes 2. [ ] No<br>d) 1. [ ] Yes 2. [ ] No	
F57.	In your relationship with your wife/partner, how often would you say that you quarreled? Would you say rarely, sometimes or often?	1. [ ] Often<br>2. [ ] Sometimes<br>3. [ ] Rarely (none)	
F58.	Now, I'd like to ask you some questions about how you interact with your wife. In the **last twelve months**, did you ever:	If No Skip to Next<br>1 Yes<br>2 No	**If YES**, How often did this happen **in the last 12 months**: often, only sometimes or rarely<br>1. [ ] Often<br>2. [ ] Sometimes<br>3. [ ] Rarely
	a) Belittle or humiliate her in front of other people?	a) 1. [ ] Yes<br>2. [ ] No	aa) 1. [ ] Often<br>2. [ ] Sometimes<br>3. [ ] Rarely
	b) Insult or make her feel bad about yourself?	b) 1. [ ] Yes<br>2. [ ] No	bb) 1. [ ] Often<br>2. [ ] Sometimes<br>3. [ ] Rarely
	c) Do things to scare or intimidate her on purpose (eg by the way you looked at her, by yelling and smashing things)?	c) 1. [ ] Yes<br>2. [ ] No	cc) 1. [ ] Often<br>2. [ ] Sometimes<br>3. [ ] Rarely
	d) Threaten to hurt her or someone she cares about?	d) 1. [ ] Yes<br>2. [ ] No	dd) 1. [ ] Often<br>2. [ ] Sometimes<br>3. [ ] Rarely

F59.	Have you ever done any of the following things to your wife	A.<br>**If Yes, continue with**<br><br>**B. If NO, skip to next item.**	B.<br>Has this happened in the past 12 months?<br><br>**(If YES ask C only. If NO ask D only.)**	C.<br>**In the past 12 months** would you say this has happened<br>1 many time<br>2.few times<br>3 once,?<br>**(After answering C, skip D)**	D.<br>**Before the past 12 months** would you say that this happened<br>1 many time<br>2.few times<br>3 once,?
	a) Slapped you or thrown something at her that could hurt her?	a) 1. [ ] Yes<br>2. [ ] No	a) 1. [ ] Yes<br>2. [ ] No	a) 1. [ ]<br>2. [ ]<br>3. [ ]	a) 1. [ ]<br>2. [ ]<br>3. [ ]
	b) Pushed her or shoved her?	b) 1. [ ] Yes<br>2. [ ] No	b) 1. [ ] Yes<br>2. [ ] No	b) 1. [ ]<br>2. [ ]<br>3. [ ]	b) 1. [ ]<br>2. [ ]<br>3. [ ]
	c) Hit her with your fist or something that could hurt her?	c) 1. [ ] Yes<br>2. [ ] No	c) 1. [ ] Yes<br>2. [ ] No	c) 1. [ ]<br>2. [ ]<br>3. [ ]	c) 1. [ ]<br>2. [ ]<br>3. [ ]
	d) Kicked her, dragged her or beaten her up?	d) 1. [ ] Yes<br>2. [ ] No	d) 1. [ ] Yes<br>2. [ ] No	d) 1. [ ]<br>2. [ ]<br>3. [ ]	d) 1. [ ]<br>2. [ ]<br>3. [ ]
	e) Choked or burnt her on purpose?	e) 1. [ ] Yes<br>2. [ ] No	e) 1. [ ] Yes<br>2. [ ] No	e) 1. [ ]<br>2. [ ]<br>3. [ ]	e) 1. [ ]<br>2. [ ]<br>3. [ ]
	f) Threatened to use or actually used a gun, knife or other weapon against her?	f) 1. [ ] Yes<br>2. [ ] No	f) 1. [ ] Yes<br>2. [ ] No	f) 1. [ ]<br>2. [ ]<br>3. [ ]	f) 1. [ ]<br>2. [ ]<br>3. [ ]
	g) Physically force her to have sexual intercourse with you even when she did not want to?	g) 1. [ ] Yes<br>2. [ ] No	g) 1. [ ] Yes<br>2. [ ] No	g) 1. [ ]<br>2. [ ]<br>3. [ ]	g) 1. [ ]<br>2. [ ]<br>3. [ ]
	h) Force her to perform sexual acts she did not want to?	h) 1. [ ] Yes<br>2. [ ] No	h) 1. [ ] Yes<br>2. [ ] No	h) 1. [ ]<br>2. [ ]<br>3. [ ]	h) 1. [ ]<br>2. [ ]<br>3. [ ]
	i) Did your wife ever have sexual intercourse with you because she was intimidated by you or afraid you would hurt her?<br>**Note:**<br>**If the respondent reports no violence >>>>>>>>>SKIP TO F61**	i) 1. [ ] Yes<br>2. [ ] No	i) 1. [ ] Yes<br>2. [ ] No	i) 1. [ ]<br>2. [ ]<br>3. [ ]	i) 1. [ ]<br>2. [ ]<br>3. [ ]

F60.	Are there any particular situations that tend to lead to violence? **Do not read options.**<br><br>Probe: Any other situation?<br><br>MARK ALL MENTIONED	1. [ ] No particular reason<br>2. [ ] When you are drunk<br>3. [ ] Money problems<br>4. [ ] Difficulties at your work<br>5. [ ] When you are unemployed<br>6. [ ] No food at home<br>7. [ ] Problems with his or her family<br>8. [ ] She is pregnant<br>9. [ ] He is jealous of her<br>10. [ ] She refuses sex<br>11. [ ] She is disobedient<br>12. [ ] Other:________________________
F61.	These next questions are about your childhood and your family. These questions will ask you about your life when you were growing up and the relationship you had with your parents or the people who cared for you.	
F62.	When you were growing up, would you say that your biological mother was never at home, rarely at home or often at home?<br>Childhood is time between the age of 00 to 15 years	1. [ ] Often at home<br>2. [ ] Rarely at home<br>3. [ ] Never at home
F63.	When you were growing up, would you say that your biological father was never at home, rarely at home or often at home?	1. [ ] Often at home<br>2. [ ] Rarely at home<br>3. [ ] Never at home
F64.	Apart from your biological father, were there other important male figures in your life when you were growing up?	1. [ ] Yes<br>2. [ ] No
F65.	Who had the final word in your household about decisions involving you and your brothers and sisters (their schooling, their activities)? Would you say your mother, father or both equally?	1. [ ] Mother<br>2. [ ] Father<br>3. [ ] Both equally<br>4. [ ] Other<br>______________________________
F66.	Who had the final word about decisions involving how your family spent money on food and clothing? Would you say your mother, father or both equally?	1. [ ] Mother<br>2. [ ] Father<br>3. [ ] Both equally<br>4. [ ] Other<br>______________________________
F67.	Who had the final word about decisions involving how your family spent money on large investments such as buying a car, a house or a household appliance? Would you say your mother, father or both equally?	1. [ ] Mother<br>2. [ ] Father<br>3. [ ] Both equally<br>4. [ ] Other<br>______________________________

This section will ask you about your knowledge of some laws and policies in your country.

F68.	According to the law, is a husband who forces his wife to have sex against her will committing a criminal act (That is, the husband can be fined or put in jail)?	1. [ ] Yes<br>2. [ ] No<br>3. [ ] Don't know
F69.	Are there any laws in your country about violence against women?	1. [ ] Yes<br>2. [ ] No<br>3. [ ] Don't know
F70.	Have you ever heard of any campaigns or activities in your community or workplace that talk about preventing violence against women?	1. [ ] Yes<br>2. [ ] No<br>3. [ ] Don't know
F71.	Have you ever seen an advertisement or public service announcement on television about violence against women?	1. [ ] Yes<br>2. [ ] No<br>3. [ ] Don't know
F72.	Have you ever participated in an activity (group session, rally, etc.) in your community or workplace on violence against women?	1. [ ] Yes<br>2. [ ] No<br>3. [ ] Don't know

F73 Anonymous picture technique

I would now like to give you a card with photos. On this card are two pictures, but no other message. The first picture is of a happy face, the second is of a sad face.

No matter what you have already told me!! I would like you to put a mark next to the sad picture if your husband has ever committed any act of physical or sexual violence against you. Please put a mark next to the happy face if this has never happened to you.

Once you have marked the card, please fold it over and put it in an envelope and then on this bag, along with many other women's responses from this village. This will ensure that I do not know your answer.

GIVE RESPONDENT CARD AND PEN. DO NOT LOOK AT RESPONSE. ONCE CARD IS FOLDED, ASK REPONDENT TO PUT IT IN AN ENVELOPE AND INTO A BAG THAT ALSO CONTAINS OTHER COMPLETED CARDS. DO NOT RECORD DETAILS OF QUESTIONNAIRE IDENTIFICATION ON CARD.

Section G.

a) Knowledge about HIV/AIDS

G1.	Now I would like to talk to you about something else. Have you ever heard of an illness called AIDS?	1. [ ] YES<br>2. [ ] NO
G2.	Where did you hear about it?<br><br>Tick all that are mentioned	1. [ ] Media (tv, radio, newspaper)<br>2. [ ] Pamphlet/leaflet/poster<br>3. [ ] Spouse<br>4. [ ] Neighbor/friend<br>5. [ ] Community Conversations program<br>6. [ ] Other program (ie NGO)<br>7. [ ] Health Worker<br>8. [ ] Other:_____________________
G3.	Have you ever discussed HIV/AIDS with anyone?	1. [ ] YES<br>2. [ ] NO **>>>>>>>>>>> SKIP TO QUESTION G5**
G4.	Who have you discussed HIV/AIDS with?	1.____<br><br>2.____<br><br>3.____<br><br>4.____
G5.	Please mention all of the ways you believe a person can get infected with HIV/AIDS.<br><br>***Do NOT read the list, tick off all that are mentioned.***	1. [ ] Sexual intercourse<br>2. [ ] Sharing needles (drug use)<br>3. [ ] Unclean medical equipment<br>4. [ ] Blood transfusions<br>5. [ ] During pregnancy<br>6. [ ] During birth<br>7. [ ] Through breast milk<br>8. [ ] Mosquito/insect bites<br>9. [ ] Contact with blood of infected person<br>10. [ ] Contact with infected person's toothbrush<br>11. [ ] Casual contact with infected person (i.e. sharing food, cup, glass, handshake, hugging, clothes)<br>12. [ ] Other (specify)______________________________<br>13. [ ] Has not heard about HIV/AIDS<br>14. [ ] Don't know/Don't remember
G6.	Is there anything a person can do to avoid getting infected with HIV, the virus that causes AIDS?	1. [ ] YES<br>2. [ ] NO **>>>>>>>>>>>SKIP TO QUESTION G8**<br>3. [ ] Don't Know

G7.	Can you tell me all the ways you know of that people can protect themselves from HIV?<br><br>***Tick all that are mentioned.***	1. [ ] Abstinence<br>2. [ ] Being faithful<br>3. [ ] Using condoms<br>4. [ ] Using condoms correctly and consistently<br>5. [ ] Going for VCT before engaging in sex<br>6. [ ] Not sharing sharp objects<br>7. [ ] Avoiding drugs/alcohol/anything which hampers judgment<br>8. [ ] Avoiding bad company<br>9. [ ] Avoiding prostitution<br>10. [ ] Avoiding walking alone at night<br>11. [ ] Avoiding contact between bloody wounds and skin<br>12. [ ] Avoiding circumcision with unsafe tools<br>13. [ ] Avoiding wife-inheritance<br>14. [ ] Avoiding sugar daddies/mummies<br>15. [ ] Avoiding multiple sexual partners<br>16. [ ] Voluntary Male circumcision<br>17. [ ] Other (specify___________________________<br>18. [ ] Don't know/ Don't remember
G8.	Do you think that a healthy-looking person can have HIV?	1. [ ] YES<br>2. [ ] NO<br>3. [ ] Don't Know
G9.	Can a pregnant woman infected with HIV transmit the virus to her baby?	1. [ ] YES<br>2. [ ] NO<br>3. [ ] Don't Know
G10.	Can a mother infected with HIV transmit the virus to her newborn baby through breastfeeding?	1. [ ] YES<br>2. [ ] NO<br>3. [ ] Don't Know
G11.	Can a person get the HIV virus by sharing a meal with someone who is infected?	1. [ ] YES<br>2. [ ] NO<br>3. [ ] Don't Know
G12.	Can people protect themselves from getting the HIV virus by avoiding mosquito bites?	1. [ ] YES<br>2. [ ] NO<br>3. [ ] Don't Know

b) Attitudes about HIV/AIDS

G13.	Do you personally know anyone who has HIV/AIDS or who died from AIDS?	1. [ ] YES<br>2. [ ] NO<br>3. [ ] Don't Know
G14.	If a member of your family got infected with the AIDS virus, would you want it to remain a secret or not?	1. [ ] YES, remain a secret<br>2. [ ] NO<br>3. [ ] Don't Know/not sure/depends
G15.	If a member of your family got infected with the AIDS virus, would you be willing to care for her or him in your own household?	1. [ ] YES<br>2. [ ] NO<br>3. [ ] Don't Know/not sure/depends

G16.	Would you buy fresh vegetables from a shopkeeper or vendor if you knew that the person had the AIDS virus?	1. [ ] YES<br>2. [ ] NO<br>3. [ ] Don't Know
G17.	In your opinion, if a female teacher has the AIDS virus but is not sick, should she be allowed to continue teaching in the school?	1. [ ] YES<br>2. [ ] NO<br>3. [ ] Don't Know
G18.	Do you know of a place where people can go to get tested for the AIDS virus?	1. [ ] YES<br>2. [ ] NO<br>3. [ ] Don't Know
G19.	Where is that?<br>Any other place?<br>(Tick all that are mentioned)	1. [ ] Hospital<br>2. [ ] Health center<br>3. [ ] Stand alone VCT center<br>4. [ ] Family planning clinic<br>5. [ ] Mobile clinic<br>6. [ ] Pharmacy<br>7. [ ] Other ________________
G20.	I don't want to know the results, but have you ever had a blood test for HIV?	1. [ ] YES<br>2. [ ] NO **>>>>>>>>>>SKIP TO G24**
G21.	How many times have you had a blood test for HIV?	|__|__| times
G22.	When was the last time you had a blood test for HIV/AIDS?<br>After entering the year, if the month unknown, enter 01 on the month	|__|__| month |__|__| year
G23.	I don't want to know the results, but did you get the results of the test?	1. [ ] YES<br>2. [ ] NO
G24.	Do you know a place where you can get condoms?	1. [ ] YES<br>2. [ ] NO **>>>>>>>>>>SKIP TO G26**
G25.	Where can you get condoms?<br>(tick all that are mentioned)	1. [ ] Hospital<br>2. [ ] Health center/clinic<br>3. [ ] Mobile clinic<br>4. [ ] Community health worker<br>5. [ ] Pharmacy<br>6. [ ] Street vendor<br>7. [ ] Bars/clubs<br>8. [ ] Shop<br>9. [ ] Friends/relative<br>10 [ ] Church<br>11. [ ] NGO<br>12. [ ] Other (specify)_________

FO: The objective of the next section is to find out your opinions about HIV/AIDS. I will read a statement. Please tell me if you strongly agree/agree/neither agree nor disagree/ disagree or strongly disagree with each statement.

Statements		Answer					
		**Strongly Agree**	**Agree**	**Neither Agree nor Disagree**	**Disagree**	**Strongly Disagree**	**Don't Know**
G26.	Using condoms reduces sexual pleasure.	1	2	3	4	5	0
G27.	It is embarrassing to buy or ask for condoms.	1	2	3	4	5	
G28.	Using a condom is a sign of not trusting your partner.	1	2	3	4	5	
G29.	Men need to have more than one sexual partner, often at the same time.	1	2	3	4	5	
G30.	HIV/AIDS is a punishment for bad behavior.	1	2	3	4	5	
G31.	It is women prostitutes that spread HIV in our community.	1	2	3	4	5	
G32.	It is promiscuous men that spread HIV in our community.	1	2	3	4	5	

c) Behavior Questions

FO: Now I am going to ask you some more questions. These ones will be about some topics that may seem embarrassing. Again, this is totally confidential and I won't share this information with anyone else.

G33.	How old were you when you had sexual intercourse for the first time?	|__|__| years
G34.	In your **lifetime**, how many sexual partners have you had?<br>Adding the current spouse	|__|__| partners
G35.	In the **last 6 months**, how many sexual partners have you had?<br>Adding the current spouse	|__|__| partners
G36.	Have you **ever** used a condom?	1. [ ] YES<br>2. [ ] NO **>>>>>>>> skip to question G40**
G37.	Did you use a condom **last time you had sex**?	1. [ ] YES<br>2. [ ] NO **>>>>>>>> skip to question**

		G39<br>3. [ ] Not sure **>>>> skip to question G39**
G38.	What were the reasons you used a condom?<br><br>*(tick all that are mentioned)*	1. [ ] Own concern to prevent pregnancy<br>2. [ ] Own concern to prevent STI/HIV<br>3. [ ] Partner insisted/ Partner's choice<br>4. [ ] Other (specify)_________________<br><br>**If any of the above answered >>> skip to G40**
G39.	What were the reasons you did not use a condom?<br><br>*(tick all that are mentioned)*	1. [ ] Condoms are uncomfortable<br>2. [ ] Embarrassing to use condoms<br>3. [ ] Unable to get condoms<br>4. [ ] Had sex with a partner who was not a sex worker<br>5. [ ] Had sex with my regular partner<br>6. [ ] Partner did not want<br>7. [ ] Other (specify)_________________
G40.	Would you like to use a condom next time/the first time you have sex?	1. [ ] YES<br>2. [ ] NO<br>3. [ ] I Don't Know
G41.	Who will make the decision about whether to use a condom?	1. [ ] Self<br>2. [ ] Partner<br>3. [ ] Both<br>4. [ ] Not sure
G42.	How confident are you that you could convince your partner to use a condom if you wanted to use one?	1. [ ] Not at all confident<br>2. [ ] Somewhat confident<br>3. [ ] Confident<br>4. [ ] Very confident
G43.	What would you do if your partner refused or declined to use a condom?	1. [ ] Have sex without condom<br>2. [ ] Talk it over and use condom<br>3. [ ] Talk it over and not use condom<br>4. [ ] Would not have sex<br>5. [ ] Other_____________________<br>6. [ ] Don't Know
G44.	Have you ever discussed HIV risk behaviors with your partner?	1. [ ] YES<br>2. [ ] NO
G45.	Now I would like to ask you some questions about your health in the last 12 months. During the last 12 months, have you had a disease which you got through sexual contact?	1. [ ] YES<br>2. [ ] NO<br>3. [ ] Don't Know
G46.	Sometimes men experience a bad smelling abnormal genital discharge. During the last 12 months, have you had a bad smelling abnormal genital discharge?	1. [ ] YES<br>2. [ ] NO<br>3. [ ] Don't Know

G47.	Sometimes men have a genital sore or ulcer. During the past 12 months, have you had a genital sore or ulcer?	1. [ ] YES<br>2. [ ] NO<br>3. [ ] Don't Know
	Enumerator Check: Did the respondent answer yes to question either G46, G47, OR D48?	**If NO >>>>>>>>End questionnaire**
G48.	The last time you had (PROBLEM FROM *G46, G47, OR D48*) did you seek any kind of advice or treatment?	1. [ ] YES<br>2. [ ] NO<br>3. [ ] Don't Know
G49.	Where did you go?	1. [ ] Community health worker<br>2. [ ] Health post<br>3. [ ] Primary health center<br>4. [ ] Government hospital<br>5. [ ] Private hospital<br>6. [ ] Private drug store<br>7. [ ] Other (specify)<br>________________________

The End

Note:

Before leaving the participant

- **Check for consistency and completeness of each question**
- **Provide greetings and thank him.**
