# Supplementary material for: Effectiveness of a culturally appropriate intervention to prevent intimate partner violence and HIV transmission among men, women, and couples in rural Ethiopia: Findings from a cluster-randomized controlled trial
Source: PLoS Med. 2020 Aug 18;17(8):e1003274. doi: 10.1371/journal.pmed.1003274 (PMC7433859; doi:10.1371/journal.pmed.1003274)
Supplement: S5 Text — (PDF) [file pmed.1003274.s008.pdf]

# Women's Health and Relationships Project in Ethiopia.

Collaborative Study by
The School of Public Health, Addis Ababa University;
The Ethiopian Public Health Association
and
The Abdul Latif Jameel Poverty Action Lab.

# Women's Questionnaire

**Addis Ababa, Ethiopia**

| QUESTIONS ABOUT THE HOUSEHOLD | | |
|---|---|---|
| | QUESTIONS & FILTERS | CODING CATEGORIES |
| 1. | If you don't mind, I would like to ask you a few questions about your household.<br>What is the main source of drinking water for your household? | TAP/PIPED WATER IN RESIDENCE .................01<br>OUTSIDE TAP (PIPED WATER).........................02<br>PUBLIC TAP ........................................................03<br>WELL WATER, WITHIN RESIDENCE ..............04<br>OUTSIDE / PUBLIC WELL..................................05<br>SPRING WATER .................................................06<br>RIVER / STREAM / POND / LAKE / DAM .......08<br>RAINWATER ........................................................09<br>TANKER / TRUCK / WATER VENDOR.............10<br>OTHER: ______________________ ...96<br>DON'T KNOW/DON'T REMEMBER ............ 98<br>REFUSED/NO ANSWER.....................................99 |
| 2. | What are the main materials used in the roof?<br>RECORD OBSERVATION | ROOF FROM NATURAL MATERIALS ...............1<br>RUDIMENTARY ROOF ........................................2<br>TILED OR CONCRETE ROOF...............................3<br>CORRUGATED IRON ...........................................4<br>OTHER: ______________________ .....6<br>DON'T KNOW/DON'T REMEMBER ............ 8<br>REFUSED/NO ANSWER.......................................9 |
| 3. | Does your household have:<br>a) Electricity<br>b) A radio<br>c) A television<br>d) A telephone<br>e) A refrigerator | YES / NO / DK<br>a) ELECTRICITY 1 2 8<br>b) RADIO 1 2 8<br>c) TELEVISION 1 2 8<br>d) TELEPHONE 1 2 8<br>e) REFRIGERATOR 1 2 8 |
| 4. | Do people in your household own any land? | YES...........................................................................1<br>NO............................................................................2<br>DON'T KNOW/DON'T REMEMBER ............ 8<br>REFUSED/NO ANSWER.......................................9 |
| 5. | How many rooms in your household are used for sleeping? | NUMBER OF ROOMS .............................. [ ][ ]<br>DON'T KNOW/DON'T REMEMBER ............ 8<br>REFUSED/NO ANSWER.......................................9 |
| 6. | Are you member of a social banking system called "Ekub" | YES...........................................................................1<br>NO............................................................................2 |
| 7. | Are you member of a social support system called "edir" | YES...........................................................................1<br>NO............................................................................2 |
| 8 | If you got something that made you happy, who would come to participate your happiness | Most of Kebele members.........................................1<br>Most of your neighbors.........................................2<br>Some of your neighbors...........................................3<br>No one will participate............................................4 |
| 9 | If you sustained a heavy problem (eg death of a family member), who would participate to support you | Most of Kebele members.........................................1<br>Most of your neighbors.........................................2<br>Some of your neighbors...........................................3<br>No one will participate............................................4 |
| 10 | In the last week, lack of money, is there a person in a family who sustained hunger per a day? | YES...........................................................................1<br>NO............................................................................2 |
| 11 | Based on your perception, how do you rank your economically with your neighbors | Higher .......................................................................1<br>Moderate .....................................................................2<br>Lower ........................................................................3 |

# ENDLINE SURVEY

Identity Number: |__|__|__| |__|__|__| |__|__|__| |__|
Woreda Kebele House No L/S

### Enumerator Information

| | |
|---|---|
| 1. Names and Codes of enumerators | ______________________________ |__|__|__| |
| 2. Name and codes of the supervisors | ______________________________ |__|__|__| |

### Identification

| | | | |
|---|---|---|---|
| 3. | District (Woreda) | W01 Meskan<br>W02 Mareko<br>W03 Silte<br>W04 Sodo | |__|__|__| |
| 4. | Village (Kebele), name and code | | |__|__|__| |
| 5. | Subvillage (Gote), name and code | | |__|__|__| |
| 6. | Name of respondent | |__________|__________|__________|<br>Name Father's Name Grand name | |
| 7. | Name of spouse | |__________|__________|__________|<br>Name Father's Name Grand name | |
| 8. | Address (write down names of road, alley, house number) Given by supervisor (computer identity) | |__|__|__| Short/ Long |__| | |
| 9. | Identity Number | |__|__|__| |__|__|__| |__|__|__| |__|<br>Woreda Kebele House No L/S | |
| 10. | Telephone/Cellphone<br>Self…. Spouse .Family… Neighbor..<br>If no phone number put 000000 | |__|__|__|__|__|__|__|__|__|__|__|<br>1. Self 2. Spouse 3 Family 4.Neighbor 5. None | |

### Results of Visits

| 11. | | First Visit | Second Visit | Third Visit |
|---|---|---|---|---|
| 12. | Date | |__|__| / |__|__| / 20|__|__|<br>Date Month Year | |__|__| / |__|__| / 20|__|__|<br>Date Month Year | |__|__| / |__|__| / 20|__|__|<br>Date Month Year |
| 13. | Time Interview Started | |__|__| : |__|__|<br>Hour Minute | |__|__| : |__|__|<br>Hour Minute | |__|__| : |__|__|<br>Hour Minute |
| 14. | Time interview completed | |__|__| : |__|__|<br>Hour Minute | |__|__| : |__|__|<br>Hour Minute | |__|__| : |__|__|<br>Hour Minute |
| 15. | Results of Visits | 1. [ ] Completed<br>2. [ ] Partly Completed<br>3. [ ] Respondent not at home<br>4. [ ] Respondent Declined<br>5. [ ] Respondent not available in the village<br>6. [ ] Other (Specify)_____<br>Make appointment if 2 or 3 answered | 1. [ ] Completed<br>2. [ ] Partly Completed<br>3. [ ] Respondent not at home<br>4. [ ] Respondent Declined<br>5. [ ] Respondent not available in the village<br>6. [ ] Other (Specify)_____<br>1. Make appointment if 2 or 3 answered | 1. [ ] Completed<br>2. [ ] Partly Completed<br>3. [ ] Respondent not at home<br>4. [ ] Respondent Declined<br>5. [ ] Respondent not available in the village<br>6. [ ] Other (Specify)_____ |

## A. Background Info/Education/Community Participation/SES

| | | |
|---|---|---|
| A1. | What is your relationship to the head of the household? | 1. [ ] Head<br>2. [ ] Wife<br>3. [ ] Co-wife |
| A2. | In what day, month and year were you born? | └─┴─┘ / └─┴─┘ / └─┴─┘<br>Day Month Year |
| A3. | How old were you at your last birthday? | └─┴─┘ Years |
| A4. | Including you, how many wives does your husband have? | └─┴─┘ wives |

## B. Gender and Domestic Violence

***FO: Now I would like to ask you questions about some other important aspects of a woman's life. I know some of these questions are very personal. However, your answers are crucial for helping to understand the condition of women in Ethiopia. Let me assure you that your answers are completely confidential and will not be told to anyone and no one else will know that you were asked these questions.***

| | | |
|---|---|---|
| B1. | Do you think violence against women is a major problem in your village? | 1. [ ] YES<br>2. [ ] NO<br>3. [ ] Don't Know |
| B2. | Out of 10 women living in your village, how many do you think are experiencing any type of physical violence from their husbands? | └─┴─┘ women<br>If unsure, enter "99" |
| B3. | Out of 10 women living in your village, how many do you think are experiencing any type of sexual violence from their husbands? | └─┴─┘ women<br>If unsure, enter "99" |
| B4. | What are some of the consequences of violence against women?<br>Tick all that are mentioned.<br>(**Do not read options**) | 1. [ ] Psychological outcomes<br>2. [ ] Physical injuries<br>3. [ ] Sexual and reproductive health problems<br>4. [ ] Suicide or death<br>5. [ ] Health or psychological risks for children<br>6. [ ] HIV or STI<br>7. [ ] Other (Specify)<br>8. [ ] Don't Know |
| B5. | How does rape expose women to HIV/AIDS?<br>Tick all that are mentioned.<br>(**Do not read options**) | 1. [ ] When couples don't openly discuss<br>2. [ ] Lack of couples trust to one another<br>3. [ ] Not willing to get advice and treatment<br>4. [ ] Other (Specify)__________________ |
| B6. | If a woman in your village was experiencing violence from her husband, who could she go to for help?<br>Tick all that are mentioned.<br>(**Do not read options**) | 1. [ ] Female relative/friend<br>2. [ ] Male relative<br>3. [ ] Police officer<br>4. [ ] Health worker<br>5. [ ] Religious leader<br>6. [ ] Community leader<br>7. [ ] Neighbor<br>8. [ ] Other: ________________________ |

| | | |
|---|---|---|
| B7. | If a woman in your village told you that she was experiencing violence from her husband, what would you do?<br><br>Tick all that are mentioned.<br>(**Do not read options**) | 1. [ ] Discuss the problem of violence with her<br>2. [ ] Encourage her to visit a health facility for treatment<br>3. [ ] Encourage her to report violence to a police officer<br>4. [ ] Encourage her to discuss this problem with traditional / religious leaders<br>5. [ ] Inform your husband about your conversation<br>6. [ ] Inform other women in the village about your conversation<br>7. [ ] Speak to the woman's husband or family about the problem of violence.<br>8. [ ] Nothing, since we should not interfere in other people's private lives.<br>9. [ ] Tell her that violence is common/normal and she should accept it.<br>10. [ ] Discourage her from telling other people<br>11. [ ] Advise her to resolve conflict elicited with her husband<br>12. [ ] Other |

| | | |
|---|---|---|
| | **When two people marry or live together, they usually share both good and bad moments. I would now like to ask you some questions about your current and past relationships and how your husband/partner treats you. If anyone interrupts us I will change the topic of conversation. I would again like to assure you that your answers will be kept secret, and that you do not have to answer any questions that you do not want to. May I continue?** | |
| B8. | I am going to read some statements about violence against women. For each statement, please state whether you agree or disagree. | |
| | a) If a man does not give his wife money for household necessities, even though he spends money on things for himself, it is an example of violence against women. | a) 1. [ ] Agree 2. [ ] Disagre |
| | b) If a man forbids his wife from seeing his parents and other family members, or friends, it is not an example of violence against women. | b) 1. [ ] Agree 2. [ ] Disagree |
| | c) If a man slaps, pushes, shoves, kicks or drags a woman, including his wife, it is not an example of violence against women. | c) 1. [ ] Agree 2. [ ] Disagree |
| | d) If a man threatens to hit a woman, but does not actually hit her, it is an example of violence against women. | d) 1. [ ] Agree 2. [ ] Disagree |
| | e) If a man forces a woman he does not know to have sex, it is an example of violence against women. | e) 1. [ ] Agree 2. [ ] Disagree |
| | f) If a man forces his wife to have sex, it is not an example of violence against women. | f) 1. [ ] Agree 2. [ ] Disagree |

| | | |
|---|---|---|
| | g) If a man shouts at his wife in public and embarrasses her, it is an example of violence against women. | g) 1. [ ] Agree 2. [ ] Disagree |
| B9. | In **the last 12 months** of your relationship with your husband/partner, how often would you say that you quarreled? Would you say rarely, sometimes or often? | 1. [ ] Rarely<br>2. [ ] Sometimes<br>3. [ ] Often |
| B10. | In the last 12 months, how are disagreements with your partner generally resolved?<br><br>Tick all that are mentioned.<br>(**Do not read options**) | 1. [ ] Through discussion<br>2. [ ] By doing what I want<br>3. [ ] By doing what my husband/partner wants<br>4. [ ] Though intervention from other family membersThrough<br>5. [ ] Through insults/forceTh<br>6. [ ] There was no conflict<br>7. [ ] Other (specify) |
| B11. | I am now going to ask you about some situations that are true for many women. Thinking about your current husband/partner, would you say in the last 12 months he: | |
| | a) Tries to keep you from seeing your friends? | a) 1. [ ] Yes 2. [ ] No |
| | b) Tries to restrict contact with your family of birth? | b) 1. [ ] Yes 2. [ ] No |
| | c) Insists on knowing where you are at all times? | c) 1. [ ] Yes 2. [ ] No |
| | d) Ignores you and treats you indifferently? | d) 1. [ ] Yes 2. [ ] No |
| | e) Gets angry if you talked to other men? | e) 1. [ ] Yes 2. [ ] No |
| | f) Is often suspicious that you are unfaithful? | f) 1. [ ] Yes 2. [ ] No |
| | g) Expects you to ask his permission before leaving home? | g) 1. [ ] Yes 2. [ ] No |
| | h) Expects you to ask his permission before seeking health care for yourself? | h) 1. [ ] Yes 2. [ ] No |
| | i) Prevent you from expressing your opinion in public? | i) 1. [ ] Yes 2. [ ] No |

| | | | | | | |
|---|---|---|---|---|---|---|
| B12. | Now, I'd like to ask you some questions about how your husband interacts with you. In the **last twelve months**, did your husband ever: | | | **If YES**, How often did this happen **in the last 12 months**: often, only sometimes or rarely | | |
| | a) Insulted you or made you feel bad about yourself? | a) 1. Yes | 2. No | aa) 1. Often | 2. Sometimes | 3. Rarely |
| | b) Belittled or humiliated you in front of other people? | b) 1. Yes | 2. No | bb) 1. Often | 2. Sometimes | 3. Rarely |
| | c) Do things to scare or intimidate you on purpose (eg by the way he looked at you, by yelling and smashing things)? | c) 1. Yes | 2. No | cc) 1. Often | 2. Sometimes | 3. Rarely |

| | | | | | | |
|---|---|---|---|---|---|---|
| | d) Threatened to hurt you or someone you care about? | d) 1. Yes | 2. No | dd) 1. Often | 2. Sometimes | 3. Rarely |
| B13. | **In the last 12 months, has your husband done any of the following things to you?** | **If NO, skip to next item** | | **Would you say this has happened?** | | |
| | a) Slapped you or thrown something at you that could hurt you? | a) 1. Yes | 2. No | aa) 1. many times | 2. few times | 3. once |
| | b) Pushed you or shoved you? | b) 1. Yes | 2. No | bb) 1. many times | 2. few times | 3. once |
| | c) Hit you with his fist or something that could hurt you? | c) 1. Yes | 2. No | cc) 1. many times | 2. few times | 3. once |
| | d) Kicked you, dragged you or beaten you up? | d) 1. Yes | 2. No | dd) 1. many times | 2. few times | 3. once |
| | e) Choked or burnt you on purpose? | e) 1. Yes | 2. No | ee) 1. many times | 2. few times | 3. once |
| | f) Threatened to use or actually used a gun, knife or other weapon against you? | f) 1. Yes | 2. No | ff) 1. many times | 2. few times | 3. once |
| | g) Physically force you to have sexual intercourse with him even when you did not want to? | g) 1. Yes | 2. No | gg) 1. many times | 2. few times | 3. once |
| | h) Force you to perform sexual acts you did not want to? | h) 1. Yes | 2. No | hh) 1. many times | 2. few times | 3. once |
| | i) Did you ever have sexual intercourse because you were intimidated by him or afraid he would hurt you? | i) 1. Yes | 2. No | ii) 1. many times | 2. few times | 3. once |

| | | | |
|---|---|---|---|
| B14. | Did your husband ever beat or assault you while you were pregnant? | 1. [ ] YES<br>2. [ ] NO | |
| B15. | As the result of actions by your husband, have you suffered any of the following in the last twelve months? | | |
| | a) Cuts, bruises or aches? | 1. [ ] Yes | 2. [ ] No |
| | b) Burns? | 1. [ ] Yes | 2. [ ] No |
| | c) Deep wounds, broken bones, broken teeth or any other serious injury | 1. [ ] Yes | 2. [ ] No |
| | d) Loss of consciousness or passing out? | 1. [ ] Yes | 2. [ ] No |
| | e) Other | e) ____________________ | |
| If no responses (or non-responses) are indicated for the above questions B12-B15, proceed to B24. | | | |
| B16. | In the past twelve months, were you ever treated badly enough by your husband that you needed health care (even if you didn't receive it)? | 1. [ ] YES<br>2. [ ] NO | |

| | | |
|---|---|---|
| B17. | Have you ever disclosed to anyone that your husband mistreats you? | 1. [ ] YES<br>2. [ ] NO **>>>>SKIP TO B24** |
| B18. | When did you first disclose this information?<br>*If more than once, record the date of the first disclosure.* | └─┴─┘ months └─┴─┘ years |
| B19. | Who did you disclose this mistreatment to in the last 12 months?<br><br>[ Check all that apply] | 1. Friends<br>2. Parents<br>3. aunt/uncle<br>4. Friend to husband<br>5. Children<br>6. Neighbors<br>7. Police<br>8. Health officer<br>9. Priest<br>10. Counselors<br>11. Non-government organization<br>12. Authorities<br>13. Elders<br>14. Other ______ |
| B20. | Did anyone try to help you in the last 12 months?<br>If yes, who helped you?<br><br>MARK ALL MENTIONED<br><br>PROBE: Anyone else? | 1. [ ] Village elders<br>2. [ ] Friends<br>3. [ ] Parents<br>4. [ ] Uncle or aunt<br>5. [ ] Husband/partner's family<br>6. [ ] Children<br>7. [ ] Neighbors<br>8. [ ] Police<br>9. [ ] Doctor/Health worker<br>10. [ ] Priest<br>11. [ ] Counselor/religious leader<br>12. [ ] NGO/Women's organization<br>13. [ ] Local leader<br>14. [ ] Other ________________<br>15. [ ] No one |
| B21. | Did you ever go to any of the following for help in the last 12 months?<br><br>READ EACH ONE, AND MARK ALL MENTIONED | 1. [ ] Police<br>2. [ ] Hospital/health center<br>3. [ ] Social services<br>4. [ ] Legal Services<br>5. [ ] Legal advice center<br>6. [ ] Court<br>7. [ ] Shelter<br>8. [ ] Local Leader<br>9. [ ] Women's organization<br>10. [ ] Priest/religious leader<br>11. [ ] Elsewhere: ______________<br>12. [ ] No where [SKIP TO B24] |
| B22. | In the last 12 months, did you ever leave, even if only overnight because of the violence? If yes, how many times? | \|__\|__\| times<br>00 If never left home<br>If 00 is entered, SKIP TO B24 |
| B23. | Where did you go last time?<br><br>Mark only one. | 1. [ ] Her relatives<br>2. [ ] His relatives<br>3. [ ] Her friends/neighbors |

| | | |
|---|---|---|
| | | 4. [ ] Hotel/lodging<br>5. [ ] Street<br>6. [ ] Church/mosque<br>7. [ ] Shelter<br>8. [ ]<br>Other:______________________ |
| B24. | Do you have any co-wives (does your husband have any other wives)? | 1. [ ] YES<br>2. [ ] NO **>>>>>>>>>>>SKIP TO B26** |
| B25. | How many of your co-wives experienced physical or sexual violence at the hands of your husband? | └─┴─┘ If unsure, enter “99” |
| B26. | Do you have any married sisters? | 1. [ ] YES<br>2. [ ] NO **>>>>>>>>>>>SKIP TO B29** |
| B27. | How many married sisters do you have? | └─┴─┘ If unsure, enter “99” |
| B28. | How many of your married sisters experienced physical or sexual violence from their husbands? | └─┴─┘ If unsure, enter “99” |
| B29. | Think about the four neighboring houses closest to you.<br>List all of the married or cohabitating women that live in those 4 houses (**List 4 names)** | 1. ______________________<br>2. ______________________<br>3. ______________________<br>4. ______________________ |
| B30. | Of the neighbors you listed, how many experienced any physical or sexual violence at the hands of their husbands in the last 12 months? | └─┴─┘ If unsure, enter “99” |
| B31. | According to the law, is a husband who forces his wife to have sex against her will committing a criminal act (That is, the husband can be fined or put in jail)? | 1. [ ] YES<br>2. [ ] NO<br>3. [ ] I don’t know |
| B32. | Are there any laws in your country about violence against women? | 1. [ ] YES<br>2. [ ] NO<br>3. [ ] I don’t know |
| NOTE TO ENUMERATOR: Read both questions to all respondents. Emphasize that we don’t want to know WHICH SPECIFIC statements are true but rather HOW MANY statements are true. It might be helpful for the respondent to count the true statements on their fingers behind their back to keep it a secret. | | |
| B33. | HOW MANY of the following statements truthfully describe your life in the **last twelve months**? Please do not tell me which statements are true, just HOW MANY..<br><br>1. I delivered a new baby.<br>2. I attended a wedding.<br>3. I received some health service at a hospital.<br>4. I purchased new clothing for myself. | └─┴─┘ statements |
| B34. | HOW MANY of the following statements truthfully describe your life in the **last twelve months**? Please do not tell me which statements are true, just HOW MANY.<br><br>1. I delivered a new baby.<br>2. I attended a wedding.<br>3. I received some health service at a hospital.<br>4. I purchased new clothing for myself.<br>5. My husband slapped me or threw something at me that could hurt me. | └─┴─┘ statements |

| | | |
|---|---|---|
| B35. | Have you ever heard of any campaigns or activities in your community or workplace that talk about preventing violence against women? | 1. [ ] YES<br>2. [ ] NO<br>3. [ ] I don't know |
| B36. | Have you ever seen an advertisement or public service announcement on television about violence against women? | 1. [ ] YES<br>2. [ ] NO<br>3. [ ] I don't know |
| B37. | Have you ever participated in an activity (group session, rally, etc.) in your community or workplace on violence against women? | 1. [ ] YES<br>2. [ ] NO<br>3. [ ] I don't know |
| NOTE TO ENUMERATOR: For the next two questions – respondents should only be asked **<u>ONE question</u>** depending on their respondent ID number.<br><br>Emphasize that we don't want to know WHICH SPECIFIC statements are true but rather HOW MANY statements are true. It might be helpful for the respondent to count the true statements on their fingers behind their back to keep it a secret.<br><br>If the last digit of the respondent ID number is **odd**, then read questions **B38 ONLY.**<br><br>If the last digit of the respondent ID number is **even**, then read question **B39 ONLY.** | | |
| B38. | HOW MANY of the following statements truthfully describe your life in the **<u>last twelve months</u>**? Please do not tell me which statements are true, just HOW MANY.<br><br>1. I have been to Addis.<br>2. My brother visited my home.<br>3. I listened to the radio almost every day.<br>4. I watched a football match. | |__|__| statements |
| B39. | HOW MANY of the following statements truthfully describe your life in the **<u>last twelve months</u>**? Please do not tell me which statements are true, just HOW MANY.<br>1. I have been to Addis.<br>2. My brother visited my home.<br>3. I listened to the radio almost every day.<br>4. I watched a football match.<br>5. I experienced physical violence such as hitting, slapping, pushing or threats of violence from my husband. | |__|__| statements |

*Enumerator: Now, I am going to read you a list of statements. For each statement, I would like you to first tell me if you agree with this statement yourself, and next tell me if you think a majority (more than half) of your friends and family agree with this statement. For example, I could read the statement "It is beneficial for all children to attend primary school." First, you would say "Yes" if you agree with this statement yourself. Next, if you believe that the majority of your friends and families also agree, you would say "Yes" again.*

| | | (a) Do you agree with this statement? | (b) Do your friends and family agree with this statement? |
|---|---|---|---|
| B40. | A man should have the final word on decisions in his home. | 1. [ ] Agree<br>2. [ ] Partially agree<br>3. [ ] Do not agree | 1. [ ] Agree<br>2. [ ] Partially agree |

| | | | |
|---|---|---|---|
| | | | 3. [ ] Do not agree<br>4. [ ] Don't know |
| B41. | A woman should obey her husband in all things. | 1. [ ] Agree<br>2. [ ] Partially agree<br>3. [ ] Do not agree | 1. [ ] Agree<br>2. [ ] Partially agree<br>3. [ ] Do not agree<br>4. [ ] Don't know |
| B42. | It is alright for a man to beat his wife if she is unfaithful. | 1. [ ] Agree<br>2. [ ] Partially agree<br>3. [ ] Do not agree | 1. [ ] Agree<br>2. [ ] Partially agree<br>3. [ ] Do not agree<br>4. [ ] Don't know |
| B43. | A man can hit his wife if she won't have sex with him. | 1. [ ] Agree<br>2. [ ] Partially agree<br>3. [ ] Do not agree | 1. [ ] Agree<br>2. [ ] Partially agree<br>3. [ ] Do not agree<br>4. [ ] Don't know |
| B44. | A woman should not initiate sex. | 1. [ ] Agree<br>2. [ ] Partially agree<br>3. [ ] Do not agree | |
| B45. | A man should be outraged if his wife asks him to use a condom. | 1. [ ] Agree<br>2. [ ] Partially agree<br>3. [ ] Do not agree | |
| B46. | It is a woman's responsibility to avoid getting pregnant. | 1. [ ] Agree<br>2. [ ] Partially agree<br>3. [ ] Do not agree | |
| B47. | A woman who has sex before she marries does not deserve respect. | 1. [ ] Agree<br>2. [ ] Partially agree<br>3. [ ] Do not agree | |
| B48. | Women should tolerate violence just in order to keep her family together. | 1. [ ] Agree<br>2. [ ] Partially agree<br>3. [ ] Do not agree | |
| B49. | There are times a woman deserves to be beaten. | 1. [ ] Agree<br>2. [ ] Partially agree<br>3. [ ] Do not agree | |
| B50. | A man using violence against his wife is a private matter that shouldn't be discussed outside the couple. | 1. [ ] Agree<br>2. [ ] Partially agree<br>3. [ ] Do not agree | |
| B51. | A man using violence against his wife is a private matter that shouldn't be discussed outside the couple. | 1. [ ] Agree<br>2. [ ] Partially agree<br>3. [ ] Do not agree | |
| B52. | It disgusts me when I see a man acting like a woman. | 1. [ ] Agree<br>2. [ ] Partially agree<br>3. [ ] Do not agree | |

***FO: Now, some people may believe that a man has a good reason to hit his wife in certain situations. I am going to read you a list of situations. For each one, please tell me if you believe that a man has a good reason to hit his wife in this situation. Please also tell me if you think most of your friends and families agree that a man would have a good reason to hit his wife in this situation.***

| | Good reason for a husband to beat his wife….. | (a) Do you agree a man would have a good reason to hit his wife in this situation? |
|---|---|---|
| B53. | She answers back to him. | 1. [ ]Yes<br>2. [ ]No |
| B54. | She neglects taking care of the children. | 1. [ ]Yes<br>2. [ ]No |
| B55. | She burns the food. | 1. [ ]Yes<br>2. [ ]No |
| B56. | She goes out without telling him. | 1. [ ]Yes<br>2. [ ]No |
| B57. | She refuses to have sex with him. | 1. [ ]Yes<br>2. [ ]No |

## C. HIV/AIDS Knowledge, Attitudes, and Perceptions

### (a) **Knowledge about HIV/AIDS**

| | | |
|---|---|---|
| C1. | Please mention all of the ways you believe a person can get infected with HIV/AIDS.<br><br>***Do NOT read the list, tick off all that are mentioned.*** | 1. [ ] Sexual intercourse<br>2. [ ] Sharing needles (drug use)<br>3. [ ] Unclean medical equipment<br>4. [ ] Blood transfusions<br>5. [ ] During pregnancy<br>6. [ ] During birth<br>7. [ ] Through breast milk<br>8. [ ] Mosquito/insect bites<br>9. [ ] Contact with blood of infected person<br>10. [ ] Contact with infected person's toothbrush<br>11. [ ] Casual contact with infected person (i.e. sharing food, cup, glass, handshake, hugging, clothes)<br>12. [ ] Other (specify) __ ______________________<br>13. [ ] Has not heard about HIV/AIDS<br>14. [ ] Don't know/Don't remember |

| | | |
|---|---|---|
| C2. | Can you tell me all the ways you know of that people can protect themselves from HIV?<br><br>***Tick all that are mentioned.*** | 1. [ ] Abstinence<br>2. [ ] Being faithful<br>3. [ ] Using condoms<br>4. [ ] Using condoms correctly and consistently<br>5. [ ] Going for VCT before engaging in sex<br>6. [ ] Not sharing sharp objects<br>7. [ ] Avoiding drugs/alcohol/anything which hampers judgment<br>8. [ ] Avoiding bad company<br>9. [ ] Avoiding prostitution<br>10. [ ] Avoiding walking alone at night<br>11. [ ] Avoiding contact between bloody wounds and skin<br>12. [ ] Avoiding circumcision with unsafe tools<br>13. [ ] Avoiding wife-inheritance<br>14. [ ] Avoiding sugar daddies/mummies<br>15. [ ] Avoiding multiple sexual partners<br>16. [ ] Voluntary male circumcision<br>17. [ ] Other (specify) ________________________<br>18. [ ] Don't know/ Don't remember |

| | | |
|---|---|---|
| C3. | Can people reduce their chance of getting the AIDS virus by having just one uninfected sex partner who has no other sex partners? | 1. [ ] YES<br>2. [ ] NO<br>3. [ ] Don't Know |
| C4. | Can people get the AIDS virus from mosquito bites? | 1. [ ] YES<br>2. [ ] NO<br>3. [ ] Don't Know |
| C5. | Can people reduce their chance of getting the AIDS virus by using a condom every time they have sex? | 1. [ ] YES<br>2. [ ] NO<br>3. [ ] Don't Know |
| C6. | Can people get the AIDS virus because of witchcraft, God's curse, or other supernatural means? | 1. [ ] YES<br>2. [ ] NO |
| C7. | Do you think that a healthy-looking person can have HIV? | 1. [ ] YES<br>2. [ ] NO<br>3. [ ] Don't Know |

## (b) Attitudes about HIV/AIDS

| | | |
|---|---|---|
| C8. | If a member of your family got infected with the AIDS virus, would you want it to remain a secret or not? | 1. [ ] YES, remain a secret<br>2. [ ] NO<br>3. [ ] Don't Know/not sure/depends |
| C9. | If a member of your family got infected with the AIDS virus, would you be wiling to care for her or him in your own household? | 1. [ ] YES,<br>2. [ ] NO<br>3. [ ] Don't Know/not sure/depends |
| C10. | Would you buy fresh vegetables from a shopkeeper or vendor if you knew that the person had the AIDS virus? | 1. [ ] YES<br>2. [ ] NO<br>3. [ ] Don't Know |
| C11. | In your opinion, if a female teacher has the AIDS virus but is not sick, should she be allowed to continue teaching in the school? | 1. [ ] YES<br>2. [ ] NO<br>3. [ ] Don't Know |
| C12. | I don't want to know the results, but have you ever had a blood test for HIV? | 1. [ ] YES<br>2. [ ] NO |

| | | |
|---|---|---|
| C13. | In your **lifetime**, how many sexual partners have you had? Including the current spouse | \|__\|__\| partners |
| C14. | Have you **ever** used a condom? | 1. [ ] YES<br>2. [ ] NO **>>>>>>>> skip to question C16** |
| C15. | Did you use a condom **last time you had sex**? | 1. [ ] YES<br>2. [ ] NO **>>>>> skip to question C18**<br>3. [ ] Not sure ›››››› **skip to question C19** |
| C16. | How confident are you that you could convince your partner to use a condom if you wanted to use one? | 1. [ ] Not at all confident<br>2. [ ] Somewhat confident<br>3. [ ] Confident<br>4. [ ] Very confident |

| | | |
|---|---|---|
| C17. | How confident are you that you know how to correctly use a condom? | 1. [ ] Not at all confident<br>2. [ ] Somewhat confident<br>3. [ ] Confident<br>4. [ ] Very confident |
| C18. | In the last 12 months, have you ever discussed HIV risk behaviors with your partner? | 1. [ ] YES<br>2. [ ] NO |
| C19. | In the last 12 months, have you ever discussed sex with your partner? | 1. [ ] YES<br>2. [ ] NO |

## D. Program questions

| | | |
|---|---|---|
| D1. | Do you remember the program “Unite for a Better Life,” a series of discussions and coffee ceremonies held in this community in 2015?<br><br>**If no- probe to make sure they don’t remember the program “Unite for Life is a program that brought people together in the community to discuss healthy relationships and gender issues”** | 1. [ ] Yes<br>2. [ ] No **>>>>> SKIP TO E1** |
| D2. | How many of these discussions did you attend? | └─┴─┘ Sessions<br>If it is 00, then go to D5 |
| D3. | If attended less than 14 sessions, why did you not attend any/ more sessions? | 1. [ ] Subject matter not interesting<br>2. [ ] Time not available (had other commitment)<br>3. [ ] Did not feel comfortable discussing those topics<br>4. [ ] Feared the disapproval of spouse<br>5. [ ] Feared the disapproval of other members of the community<br>6. [ ] Was not invited/selected for participation<br>7. [ ] Did not feel comfortable with the facilitator<br>8. [ ] Other |
| D4. | Did you attend the discussions alone or with your spouse? | 1. [ ] Always Alone<br>2. [ ] Always with my spouse<br>3. [ ] Sometimes with my spouse |
| D5. | How many sessions did your spouse attend? | └─┴─┘ Sessions |
| | **Enumerator: Check question D2 and copy response here:**<br><br>**# of sessions attended by the respondent** └─┴─┘ **Sessions IF = 0 then SKIP TO D16** | |
| D6. | If you did attend these discussions, would you say you remember most of what was discussed, some of what was discussed, only a little, or nothing at all? | 1. [ ] Most or nearly all of what was discussed<br>2. [ ] Some of what was discussed<br>3. [ ] Little of what was discussed<br>4. [ ] Nothing at all |
| D7. | What was the most important information you discussed in these sessions? | ________________________________ |

| | | |
|---|---|---|
| D8. | Did you share the information you obtained from this program with anyone? | 1. [ ] Yes<br>2. [ ] No **>>>>> SKIP to D11** |
| D9. | If yes, who did you share the information with?<br><br>Tick all that apply | 1. [ ] Spouse<br>2. [ ] Relative<br>3. [ ] Friend<br>4. [ ] Neighbor<br>5. [ ] My children<br>6. [ ] Other (specify) _______________ |
| D10. | How many people in total did you share the information with? | └─┴─┘ people |
| D11. | On a scale of 1 to 5, how satisfied were you with the program? | 1. [ ] Not at all satisfied<br>2. [ ] A little satisfied<br>3. [ ] Somewhat satisfied<br>4. [ ] Satisfied<br>5. [ ] Very satisfied |
| D12. | Were there any negative consequences of participating in these sessions? | 1. [ ] Yes<br>2. [ ] No >>>>>> **SKIP to D14** |
| D13. | If yes, what were the negative consequences of participating in these sessions?<br><br>**Tick all that apply.** | 1. [ ] Lost wages<br>2. [ ] No time to do household chores<br>3. [ ] Angered spouse when I participated<br>4. [ ] Spouse became violent when I participated<br>5. [ ] Angered family members when I participated<br>6. [ ] Family members became violent when I participated<br>7. [ ] Stigma from community members (outcast by others, name calling, gossip about you)<br>8. [ ] Other (specify) ________________ |
| D14. | How likely would you be to recommend to a friend to participate in this program? | 1. [ ] Not at all likely<br>2. [ ] A little likely<br>3. [ ] Somewhat likely<br>4. [ ] Likely<br>5. [ ] Very likely |
| D15. | Do you continue to meet the members of your Unite for a better life group to continue to discuss topics related to the program? | 1. [ ] Yes<br>2. [ ] No<br><br>**>>>>>SKIP TO E1** |
| | **QUESTIONS D16-D18 ARE ONLY FOR THOSE WHO DID NOT PARTICIPATE IN ANY SESSIONS (where the response to question D2=0)** | |
| D16. | Did anyone share information from the Unite for a better life program discussions with you? | 1. [ ] Yes<br>2. [ ] No **>>>>>>>>>>>>>>SKIP TO E1** |
| D17. | If yes, who shared the information with you? | 1. [ ] Spouse<br>2. [ ] Relative<br>3. [ ] Friend<br>4. [ ] Neighbor<br>5. [ ] Other (specify) _______________ |

| | | |
|---|---|---|
| D18. | Which specific information did they share with you? | ______________________ ______________________ |

**E. Spouse's information and work status; Respondent's relative socioeconomic status**

| | | |
|---|---|---|
| E1. | How old was your husband/partner on his last birthday? | \|__\|__\| Years |
| E2. | In the last 12 months, what has been your husband's average monthly income? | \|__\|__\|__\|__\|__\| Birr |
| E3. | Have you ever worked or done any activities for income? | 1. [ ] Yes<br>2. [ ] No **>>>>>SKIP TO E7** |
| E4. | In the last 12 months what was your average daily wage?<br>If in Kind change to a monetary equivalent | \|__\|__\|__\|__\| Birr |
| E5. | Who usually decides how the money you earn will be used: mainly you, mainly your husband/partner or you and your husband/partner jointly? | 1. [ ] Respondent<br>2. [ ] Husband/partner<br>3. [ ] Respondent and husband/partner jointly<br>4. [ ] Other_______________ |
| E6. | Would you say that the money that you earn is more than what your husband/partner earns, less than what he earns or about the same? | 1. [ ] More than him<br>2. [ ] Less than him<br>3. [ ] About the same<br>4. [ ] Husband/partner doesn't bring in money **[Skip to E8]**<br>5. [ ] Don't know |
| E7. | Who usually decides how your husband's/ partner's earnings will be used: you, your husband/partner, or you and your husband/partner jointly? | 1. [ ] Respondent<br>2. [ ] Husband/partner<br>3. [ ] Respondent and husband/partner jointly<br>4. [ ] Other_______________ |
| E8. | How many times per day or week do you participate in a traditional coffee ceremony?<br>If displayed in days, put 00 on the per-week<br>If displayed in weeks, put 00 on the per day | \|__\|__\| per day \|__\|__\| per week |

| | | |
|---|---|---|
| E9. | Who usually participates in the coffee ceremony with you?<br><br>Tick all that apply. | 1. [ ] Husband/partner<br>2. [ ] Family members<br>3. [ ] Neighbors<br>4. [ ] Village leader<br>5. [ ] Religious leader<br>6. [ ] Friends<br>7. [ ] Other______________ |
| E10. | What do you discuss with others during a coffee ceremony?<br>Tick all that apply. | 1. [ ] Family or personal issues<br>2. [ ] Issues related to your community<br>3. [ ] National or political news<br>4. [ ] Health challenges<br>5. [ ] Problems with farming or cattle<br>6. [ ] Other_____________________ |
| E11. | Who usually prepares the coffee during the traditional coffee ceremony? | 1. [ ] Men<br>2. [ ] Women<br>3. [ ] Both men and women |
| E12. | Has your husband ever prepared the coffee during the traditional coffee ceremony? | 1. [ ] Yes<br>2. [ ] No |

| E13. | Now if you permit me, I will ask some more questions about your relationship with your husband.<br><br>In the last twelve months, did your husband ever: | Yes | No | If yes, how often? | | |
|---|---|---|---|---|---|---|
| | | | | Often | Someti mes | Rarely |
| | a) Expect that you ask permission to purchase large items? | 1 | 2 | 1 | 2 | 3 |
| | b) Expect that you ask permission to purchase small items? | 1 | 2 | 1 | 2 | 3 |
| | c) Take your earnings or savings from you against your will? | 1 | 2 | 1 | 2 | 3 |
| | d) Spent money you needed for rent, food or clothes on things for him? | 1 | 2 | 1 | 2 | 3 |
| | e) Threaten to not give you money or take it away from you? | 1 | 2 | 1 | 2 | 3 |
| E14. | Who in your family usually has the final say in how you spend money on the following things? | Your self | Spouse | Yourself and spouse Jointly | Someone else | You and someone else jointly |
| | a. Food and clothing | 1 | 2 | 3 | 4 | 5 |
| | b. Large investments such as buying a car, or a house, or a household appliance | 1 | 2 | 3 | 4 | 5 |

| | | | | | | | |
|---|---|---|---|---|---|---|---|
| | c. Regarding spending time with family friends or relatives | 1 | 2 | 3 | 4 | 5 | |
| E15. | In the last 12 months, how did you and your partner divide the following tasks:<br><br>(Not applicable is coded as 0). | I do every-thing | Usually me | Shared equally or done together | Usually partner | Partner does every-thing | Some-one else |
| | a) Washing clothes | 1 | 2 | 3 | 4 | 5 | 6 |
| | b) Repairing house | 1 | 2 | 3 | 4 | 5 | 6 |
| | c) Buying food | 1 | 2 | 3 | 4 | 5 | 6 |
| | d) Cleaning the house | 1 | 2 | 3 | 4 | 5 | 6 |
| | e) Preparing the food | 1 | 2 | 3 | 4 | 5 | 6 |
| | f) Daily care of the children | 1 | 2 | 3 | 4 | 5 | 6 |

| | | |
|---|---|---|
| E16. | Are you satisfied with this division of tasks? | 1. [ ] Yes<br>2. [ ] No |
| E17. | Do you think your partner is satisfied with this division? | 1. [ ] Yes<br>2. [ ] No |

| | | |
|---|---|---|
| E18. | How often does your husband/partner drink alcohol? | 1. [ ] Every day or nearly every day<br>2. [ ] Once or twice a week<br>3. [ ] 1-3 times in a month<br>4. [ ] Rarely<br>5. [ ] Never drinks >>>>>>>> Skip to E21 |
| E19. | In the past 12 months how often have you seen your husband/partner drunk?<br>Would you say most days, weekly, once a month, less than once a month or never? | 1. [ ] Most days<br>2. [ ] Weekly<br>3. [ ] Once a month<br>4. [ ] Very Rarely<br>5. [ ] Never |
| E20. | In the past 12 months, have you experienced any of the following problems, related to your husband/partner's drinking?<br><br>a. Money problems<br>b. Health problems<br>c. Conflict with family or friends<br>d. Problems with authorities (police etc)<br>e. Any other problems (specify) | a. 1. [ ] Yes 2. [ ] No<br>b. 1. [ ] Yes 2. [ ] No<br>c. 1. [ ] Yes 2. [ ] No<br>d. 1. [ ] Yes 2. [ ] No<br><br>e. Other(Specify)__________ |
| E21. | How often does your husband/partner chew khat?<br><br>1. Every day or nearly every day<br>2. Once or twice a week<br>3. 1-3 times a month | 1. [ ] Every day or nearly every day<br>2. [ ] Once or twice a week |

| | | |
|---|---|---|
| | 4. Occasionally, less than once a month<br>5. Never | 3. [ ] 1-3 times in a month<br>4. [ ] Very Rarely<br>5. [ ] Never >>>>>>>> Skip to E23 |
| E22. | In the past 12 months, have you experienced any of the following problems, related to your husband/partner's khat chewing?<br><br>a. Money problems<br>b. Health problems<br>c. Conflict with family or friends<br>d. Problems with authorities (police etc)<br>e. Any other problems (specify) | a. 1. [ ] Yes 2. [ ] No<br>b. 1. [ ] Yes 2. [ ] No<br>c. 1. [ ] Yes 2. [ ] No<br>d. 1. [ ] Yes 2. [ ] No<br><br>e. Other(Specify)____________ |
| E23. | How many biological children do you have?<br>If no child enter 00<br>Enter 99 if child is dead | └─┴─┘ **If no child>>>>>>>skip to F1** |
| E24. | How old is your youngest biological child?<br>If the child died, fill near the age he/ she died 99 | └─┴─┘ Years<br>└─┴─┘ months (if age < 1 year, enter months)<br>└─┴─┘ days (if age <1 month, enter days) |
| E25. | How old is your eldest biological child?<br>If the child died, fill near the age he/ she died 99 | └─┴─┘ Years<br>└─┴─┘ months (if age <1 year, enter months)<br>└─┴─┘ days (if age <1 month, enter days) |
| E26. | When you were pregnant with your youngest child, how many times did your partner accompany you to an antenatal care (ANC) visit? | └─┴─┘ ANC visits<br><br>**if he did not accompany to any visits enter 00 and SKIP TO E28** |
| E27. | During the last antenatal care visit that your partner attended, did he: | 1. [ ] Drop you off at the entrance or wait for you outside<br>2. [ ] Wait in the facility, but not participate in the ANC visit<br>3. [ ] Join you for at least part of the visit with the health provider<br>4. [ ] Don't know / Don't remember |
| E28. | Where was your partner during the birth of your last child? | 1. [ ] He was in the delivery room with me<br>2. [ ] He was at the health facility/delivery location but not in the room with me<br>3. [ ] He was not at the health facility/ delivery location<br>4. [ ] Don't know / Don't remember |

## F. Patient Health Questionnaire

| Over the <u>last 2 weeks</u>, how often have you been bothered by any of the following problems? | | Answer | | | |
|---|---|---|---|---|---|
| | | Not at all | Several Days | More than half the days | Nearly every day |
| F1. | Little interest or pleasure in doing things | 0 | 1 | 2 | 3 |
| F2. | Feeling down, depressed or hopeless | 0 | 1 | 2 | 3 |
| F3. | Trouble falling or staying asleep, or sleeping too much | 0 | 1 | 2 | 3 |
| F4. | Feeling tired or having little energy | 0 | 1 | 2 | 3 |
| F5. | Poor appetite or overeating | 0 | 1 | 2 | 3 |
| F6. | Feeling bad about yourself- or that you are a failure or have let yourself or your family down | 0 | 1 | 2 | 3 |
| F7. | Trouble concentrating on things, such as reading the newspaper or watching television | 0 | 1 | 2 | 3 |
| F8. | Moving or speaking so slowly that other people could have noticed. Or the opposite – being so figety or restless that you have been moving around a lot more than usual | 0 | 1 | 2 | 3 |
| F9. | Thoughts that you would be better off dead, or of hurting yourself | 0 | 1 | 2 | 3 |
| | | **Not difficu lt at all** | **Some what difficu lt** | **Very difficult** | **Extrem ely difficult** |
| F10. | If you checked off any problems, how difficult have these problems made it for you to do your work, take care of things at home, or get along with other people? | 0 | 1 | 2 | 3 |

**Comments:**

______________________________________________

______________________________________________

______________________________________________

## <u>G0. Anonymous picture technique</u>

I would now like to give you a card with photos. On this card are two pictures, but no other message. The first picture is of a happy face, the second is of a sad face.

No matter what you have already told me, I would like you to put a mark next to the sad picture if your husband has ever committed any act of physical violence such as hitting, slapping, pushing or threats of violence or sexual violence against you <u>**in the last 12 months**</u>. Please put a mark next to the happy face if this has never happened to you.

Once you have marked the card, please fold it over and put it in an envelope and then on this bag, along with many other women's responses from this village. This will ensure that I do not know your answer. Your response will remain completely anonymous and there is no way for anyone to know what your answer is. Your confidentiality will be protected, so please feel free to share the honestly without fear.

GIVE RESPONDENT CARD AND PEN. DO NOT LOOK AT RESPONSE. ONCE CARD IS FOLDED, ASK REPONDENT TO PUT IT IN AN ENVELOPE AND INTO A BAG THAT ALSO CONTAINS OTHER COMPLETED CARDS. DO NOT RECORD DETAILS OF QUESTIONNAIRE IDENTIFICATION ON CARD.

# The End

## Note:

**Before leaving the participant**

- **Check for consistency and completeness of each question**
- **Provide the referral and community resource after informing her**
- **Provide greetings and thank her.**
