# Supplementary material for: Effectiveness of a culturally appropriate intervention to prevent intimate partner violence and HIV transmission among men, women, and couples in rural Ethiopia: Findings from a cluster-randomized controlled trial
Source: PLoS Med. 2020 Aug 18;17(8):e1003274. doi: 10.1371/journal.pmed.1003274 (PMC7433859; doi:10.1371/journal.pmed.1003274)
Supplement: S6 Text — (PDF) [file pmed.1003274.s009.pdf]

Women’s Health and Relationships Project in Ethiopia.

Collaborative Study by

The School of Public Health, Addis Ababa University;

The Ethiopian Public Health Association

and

The Abdul Latif Jameel Poverty Action Lab

Men’s Questionnaire
የወንዶች መጠይቅ

Addis Ababa, Ethiopia

BASELINE SURVEY

Identity Number: : |__|__|__| |__|__|__| |__|__|__| |__|
Woreda Kebele House No L/S

Enumerator Information

1. Names and Codes of enumerators	________________________________ |__|__|__|
2. Name and codes of the supervisors	________________________________ |__|__|__|

Identification

3.	District (Woreda)	W01 Meskan<br>W02 Mareko<br>W03 Silte<br>W04 Sodo	|__|__|__|
4.	Village (Kebele), name and code		|__|__|__|
5.	Subvillage (Gote), name and code		|__|__|__|
6.	Name of respondent	Name / Father's Name / Grand name	
7.	Name of spouse	Name / Father's Name / Grand name	
8.	Address (write down names of road, alley, house number) Given by supervisor (computer identity)	|__|__|__| Short/ Long |__|	
9.	Identity Number	|__|__|__| |__|__|__| |__|__|__| |__| Woreda Kebele House No L/S	
10.	Telephone/Cellphone<br>Self…. Spouse .Family… Neighbor..<br>If no phone number put 000000	|__|__|__|__|__|__|__|__|__|__| 1. Self 2. Spouse 3 Family 4.Neighbor 5. None	

Results of Visits

11.		First Visit	Second Visit	Third Visit
12.	Date	|__|__| / |__|__| / 20|__|__| Date Month Year	|__|__| / |__|__| / 20|__|__| Date Month Year	|__|__| / |__|__| / 20|__|__| Date Month Year
13.	Time Interview Started	|__|__| : |__|__| Hour Minute	|__|__| : |__|__| Hour Minute	|__|__| : |__|__| Hour Minute
14.	Time interview completed	|__|__| : |__|__| Hour Minute	|__|__| : |__|__| Hour Minute	|__|__| : |__|__| Hour Minute
15.	Results of Visits	1. [ ] Completed<br>2. [ ] Partly Completed<br>3. [ ] Respondent not at home<br>4. [ ] Respondent Declined<br>5. [ ] Respondent not available in the village<br>6. [ ] Other (Specify)_____<br>Make appointment if 2 or 3 answered	1. [ ] Completed<br>2. [ ] Partly Completed<br>3. [ ] Respondent not at home<br>4. [ ] Respondent Declined<br>5. [ ] Respondent not available in the village<br>6. [ ] Other (Specify)_____<br>1. Make appointment if 2 or 3 answered	1. [ ] Completed<br>2. [ ] Partly Completed<br>3. [ ] Respondent not at home<br>4. [ ] Respondent Declined<br>5. [ ] Respondent not available in the village<br>6. [ ] Other (Specify)_____

A. Background Info/Education/Community Participation/SES

A1.	In what day, month and year were you born?<br>If he does not know his date of birth – write 01/01/year	└─┴─┘ / └─┴─┘ / └─┴─┴─┴─┘<br>Day month Year
A2.	How old were you at your last birthday?	└─┴─┘ Years
A3.	How many wives do you have?	└─┴─┘ wives
A4.	How old was your wife/ main partner on her last birthday? Compute with your age	└─┴─┘ Years

B. Wife's background information, Employment status, and man's economic and Employment status

B1.	How many times per week or per day you participate in a traditional coffee ceremony?<br>If answered per week, enter 00 on the day<br>If answered per day, enter 00 on the weeks	└─┴─┘ per day └─┴─┘ per week
B2.	Who usually participates in the coffee ceremony with you?<br>Tick all that apply	1. [ ] wife/partner<br>2. [ ] Family members<br>3. [ ] Neighbors<br>4. [ ] Village leader<br>5. [ ] Religious leader<br>6. [ ] Friends<br>7. [ ] Other______________
B3.	What do you discuss with others during a coffee ceremony?<br>Tick all that apply	1. [ ] Family or personal issues<br>2. [ ] Issues related to your community<br>3. [ ] National or political news<br>4. [ ] Health challenges<br>5. [ ] Problems with farming or cattle<br>6. [ ] Other (Specify)__________________
B4.	Who usually prepares the coffee during the traditional coffee ceremony?	1. [ ] Men<br>2. [ ] Women<br>3. [ ] Both men and women
B5.	Have you ever prepared the coffee during the traditional coffee ceremony?	1. [ ] Yes<br>2. [ ] No
B6.	Would you be happy to participate in a coffee ceremony if a man prepared the coffee?	1. [ ] Yes<br>2. [ ] No

				If Yes, how often?		
B7.	Now if you permit me, I will ask some more questions about your relationship with your wife. In the last twelve months, did you ever:	Yes	No	Often	Sometimes	Rarely
	a) Expect that your wife ask permission to purchase large items?	1	2	1	2	3
	b) Expect that your wife ask permission to purchase small items?	1	2	1	2	3

	c) Taken her earnings or savings from her against her will?	1	2	1	2	3	
	d) Spent money she needed for rent, food or clothes on things for yourself?	1	2	1	2	3	
	e) Threaten to not give her money or take it away from her	1	2	1	2	3	
B8.	Who in your family usually has the final say in how you spend money on the following things?	Your self	Spouse	Yourself and spouse Jointly	Someone else	You and someone else jointly	
	a. Food and clothing	1	2	3	4	5	
	b. Large investments such as buying a car, or a house, or a household appliance	1	2	3	4	5	
	c. Regarding spending time with family friends or relatives	1	2	3	4	5	
B9.	In the last 12 months, how did you and your partner divide the following tasks:<br><br>(Not applicable is coded as 0).	I do every thing	Usually me	Shared equally or done together	Usually partner	Partner does every-thing	Some-one else
	a) Washing clothes	1	2	3	4	5	6
	b) Repairing house	1	2	3	4	5	6
	c) Buying food	1	2	3	4	5	6
	d) Cleaning the house	1	2	3	4	5	6
	e) Cleaning the compound and toilet	1	2	3	4	5	6
	f) Preparing the food	1	2	3	4	5	6
	h) Daily care of the children	1	2	3	4	5	6

B10.	Are you satisfied with this division of tasks?	1. [ ] Yes<br>2. [ ] No
B11.	Do you think your partner is satisfied with this division?	1. [ ] Yes<br>2. [ ] No

B12.	How often have you drank alcohol (liquor, tej, beer) in the past 12 months?<br><br>1. Every day or nearly every day<br>2. Once or twice a week<br>3. 1-3 times a month<br>4. Occasionally, less than once a month<br>5. Never	1. [ ] Every day or nearly every day<br>2. [ ] Once or twice a week<br>3. [ ] 2-3 times in a month<br>4. [ ] Once a month<br>5. [ ] Never **>>>>SKIP TO B14**
B13.	In the past 12 months how often have you been drunk?<br><br>Would you say most days, weekly, once a month, less than once a month or never?	1. [ ] Most days<br>2. [ ] Weekly<br>3. [ ] Once a month<br>4. [ ] Very Rarely<br>5. [ ] Never
B14.	In the past 12 months, have you experienced any of the following problems, related to your drinking?<br><br>a. Money problems<br>b. Health problems<br>c. Conflict with family or friends<br>d. Problems with authorities (police etc)<br><br>e. Any other problems (specify)	a. 1. [ ] Yes 2. [ ] No<br>b. 1. [ ] Yes 2. [ ] No<br>c. 1. [ ] Yes 2. [ ] No<br>d. 1. [ ] Yes 2. [ ] No<br><br>e. Other(Specify)_______________
B15.	How often do you chew khat?<br><br>1. Every day or nearly every day<br>2. Once or twice a week<br>3. 1-3 times a month<br>4. Occasionally, less than once a month<br>5. Never<br>6. Don't Know	1. [ ] Every day or nearly every day<br>2. [ ] Once or twice a week<br>3. [ ] 1-3 times in a month<br>4. [ ] Less than once a month<br>5. [ ] Never **>>> Skip to C1**<br>6. [ ] Don't know
B16.	In the past 12 months, have you experienced any of the following problems, related to your khat chewing?<br><br>a. Money problems<br>b. Health problems<br>c. Conflict with family or friends<br>d. Problems with authorities (police etc)<br><br>e. Any other problems (specify)	a. 1. [ ] Yes 2. [ ] No<br>b. 1. [ ] Yes 2. [ ] No<br>c. 1. [ ] Yes 2. [ ] No<br>d. 1. [ ] Yes 2. [ ] No<br><br>e. Other(Specify)_______________

C. Fatherhood

Thank you for doing so, your answers are really important. The next questions are about you and the children you may have fathered or adopted or children who may live with you even if they are not legally or biologically yours.

C1.	How many biological children do you have?<br>If no child enter 00	|__|__| If no child,>>>>>> skip to C4

C2.	How old is your youngest biological child?<br>If the child died, fill near the age he/ she died 99	└┴┘ Years<br>└┴┘ months (if age < 1 year, enter months)<br>└┴┘ days (if age <1 month, enter days)
C3.	How old is your eldest biological child?<br>If the child died, fill near the age he/ she died 99	└┴┘ Years<br>└┴┘ months (if age <1 year, enter months)<br>└┴┘ days (if age <1 month, enter days)
C4.	Did you accompany the mother of your **youngest child** to any prenatal visits during the last pregnancy?	1. [ ] Yes<br>2. [ ] No >>>>>SKIP TO C6<br>3. [ ] She didn't have ANC visits **>>>SKIP TO C6**<br>4. [ ] She has never been pregnant **>SKIP TO D1**
C5	During the last antenatal care visit that your attended, did you:	1. [ ] Drop your partner off at the entrance or wait for her outside<br>2. [ ] Wait in the facility, but not participate in the ANC visit<br>3. [ ] Join her for at least part of the visit with the health provider<br>4. [ ] Don't know / Don't remember
C6.	Where were you during the birth of your last child?	1. Were you at the delivery or around<br>2. I was not in the area<br>3. I don't know, don't remember<br>4. Was delivered at home, skip to D1<br>5. Last child was not delivered, skip D1
C7.	Did you choose not to be present at the birth or did you want to be present but were prevented from doing so for some reason?	1. [ ] Wanted to be present<br>2. [ ] Didn't want to be present<br>3. [ ] Don't know / don't remember

D. Gender and Domestic Violence

FO: Now I would like to ask for your opinion on some other important aspects of a woman's life. I know some of these questions are very personal. However, your answers are crucial for helping to understand the condition of women in Ethiopia. Let me assure you that your answers are completely confidential and will not be told to anyone and no one else will know that you were asked these questions.

D1.	Do you think violence against women is a major problem in your village?	1. [ ] YES<br>2. [ ] NO<br>3. [ ] Don't Know
D2.	Out of 10 women living in your village, how many do you think are experiencing any type of physical violence from their husbands?	└┴┘ women
D3.	Out of 10 women living in your village, how many do you think are experiencing any type of sexual violence from their husbands?	└┴┘ women

D4.	What are some of the consequences of violence against women?<br>Tick all that are mentioned.<br>(**Do not read options**)	1. [ ] Psychological outcomes<br>2. [ ] Physical injuries<br>3. [ ] Sexual and reproductive health problems<br>4. [ ] Suicide or death<br>5. [ ] Health or psychological risks for children<br>6. [ ] HIV/ AIDS or STD transmission<br>7. [ ] Other (specify) ___________________<br>8. [ ] Don't know
D5.	How does violence against women increase their vulnerability to HIV/AIDS?<br>Tick all that are mentioned.<br>(**Do not read options**)	1. [ ] Lack of the couple openness<br>2. [ ] Lack of trust<br>3. [ ] Restricting themselves from medical attention and knowledge<br>4. [ ] Other (specify)
D6.	If a woman in your village was experiencing violence from her husband, who could she go to for help?<br>(**Do not read options**	1. [ ] Female relative/friend<br>2. [ ] Male relative<br>3. [ ] Police officer<br>4. [ ] Health worker<br>5. [ ] Religious leader<br>6. [ ] Community leader<br>7. [ ] Neighbor<br>8. [ ] Other: ________________________

D7.	What would you do if you saw a male friend use physical or sexual violence against a woman?<br><br>Tick all that are mentioned.<br>(**Do not read options**)	1. [ ] Intervene during the episode<br>2. [ ] Speak to him after the episode<br>3. [ ] Avoid/shun him<br>4. [ ] Call the police<br>5. [ ] Do nothing, as it is their problem<br>6. [ ] Mobilize neighbors or friends<br>7. [ ] Contact village elders<br>8. [ ] Other
D8.	What would you do if you saw physical or sexual violence being carried out by a stranger (man) against a woman?<br><br>Tick all that are mentioned.<br>(**Do not read options**)	1. [ ] Intervene during the episode<br>2. [ ] Speak to him after the episode<br>3. [ ] Avoid/shun him<br>4. [ ] Call the police<br>5. [ ] Do nothing, as it is their problem<br>6. [ ] Mobilize neighbors or friends<br>7. [ ] Contact village elders<br>8. [ ] Other
	**When two people marry or live together, they usually share both good and bad moments. I would now like to ask you some questions about your current and past relationships and how you treat your wife. If anyone interrupts us I will change the topic of conversation. I would again like to assure you that your answers will be kept secret, and that you do not have to answer any questions that you do not want to. May I continue?**	
D9.	In general, do you and your partner discuss the following topics together:<br><br>a) Things that have happened to you in the day	 <br>a) 1. [ ] Yes 2. [ ] No

	b) Things that happened to her during the day	b) 1. [ ] Yes 2. [ ] No				
	c) Your worries or feelings	c) 1. [ ] Yes 2. [ ] No				
	d) Her worries or feelings	d) 1. [ ] Yes 2. [ ] No				
D10.	In **<u>the last 12 months</u>** of your relationship with your wife/partner, how often would you say that you quarreled? Would you say rarely, sometimes or often?	1. [ ] Rarely<br>2. [ ] Sometimes<br>3. [ ] Often				
D11.	In the last 12 months, how are disagreements with your partner generally resolved?	1. [ ] There are few disagreements<br>2. [ ] Through discussion and communication<br>3. [ ] By doing what I want<br>4. [ ] By doing what my wife/partner wants<br>5. [ ] Through intervention from other family members<br>6. [ ] Through Insults/ force<br>7. [ ] There are no disagreements.<br>8. [ ] Other (specify)____________				
D12.	Now, I'd like to ask you some questions about how you interact with your wife. In the **<u>last twelve months</u>**, did you ever:			**If YES**, How often did this happen **<u>in the last 12 months</u>**: often, only sometimes or rarely		
	a) Insulte or make her feel bad about herself?	a) 1. Yes	2. No	aa) 1. Often	2. Sometimes	3. Rarely
	b) Belittle or humiliate her front of other people?	b) 1. Yes	2. No	bb) 1. Often	2. Sometimes	3. Rarely
	c) Do things to scare or intimidate her on purpose (eg by the way he looked at you, by yelling and smashing things)?	c) 1. Yes	2. No	cc) 1. Often	2. Sometimes	3. Rarely
	d) Threatened to hurt her or someone she cares about?	d) 1. Yes	2. No	cd) 1. Often	2. Sometimes	3. Rarely
D13.	**In <u>the last 12 months</u>, have you ever any of the following things to your wife?**	**If NO, skip to next item**		**Would you say this has happened?**		
	a) Slapped her or thrown something at her that could hurt you?	a) 1. Yes	2. No	aa) 1. many times	2. few times	3. once
	b) Pushed her or shoved her?	b) 1. Yes	2. No	bb) 1. many times	2. few times	3. once

	c) Hit her with your fist or something that could hurt her?	c) 1. Yes	2. No	cc) 1. many times	2. few times	3. once
	d) Kicked her, dragged her or beaten her up?	d) 1. Yes	2. No	dd) 1. many times	2. few times	3. once
	e) Choked or burnt her on purpose?	e) 1. Yes	2. No	ee) 1. many times	2. few times	3. once
	f) Threatened to use or actually used a gun, knife or other weapon against her?	f) 1. Yes	2. No	ff) 1. many times	2. few times	3. once
	g) Physically force her to have sexual intercourse with you even when she did not want to?	g) 1. Yes	2. No	gg) 1. many times	2. few times	3. once
	h) Force her to perform sexual acts she did not want to?	h) 1. Yes	2. No	hh) 1. many times	2. few times	3. once
	i) Did your wife ever have sexual intercourse because she was intimidated by you or afraid you would hurt her?<br><br>**Note:**<br>**If the respondent reports no violence >>>>>>>>>SKIP TO D15**	i) 1. Yes	2. No	ii) 1. many times	2. few times	3. once

D14.	Are there any particular situations that tend to lead to violence? **Do not read options.**<br><br>Probe: Any other situation?<br><br>MARK ALL MENTIONED	1. [ ] No particular reason<br>2. [ ] When you are drunk<br>3. [ ] Money problems<br>4. [ ] Difficulties at your work<br>5. [ ] When you are unemployed<br>6. [ ] No food at home<br>7. [ ] Problems with his or her family<br>8. [ ] She is pregnant<br>9. [ ] He is jealous of her<br>10. [ ] She refuses sex<br>11. [ ] She is disobedient<br>12. [ ] Other:____________________
D15.	Think about the four neighboring houses closest to you. List all of the married or cohabitating women that live in those 4 houses (**List 4 names)**	1. ________________________<br>2. ________________________<br>3. ________________________<br>4. ________________________
D16.	Of the neighbors you listed, how many experienced any physical or sexual violence at the hands of their husbands in the last 12 months?	└─┴─┘ If no neighbors experienced violence enter 00

This section will ask you about your knowledge of some laws and policies in your country.

D17.	According to the law, is a husband who forces his wife to have sex against her will committing a criminal act (That is, the husband can be fined or put in jail)?	1. [ ] Yes<br>2. [ ] No<br>3. [ ] Don't know
D18.	Are there any laws in your country about violence against women?	1. [ ] Yes<br>2. [ ] No<br>3. [ ] Don't know
NOTE TO ENUMERATOR: Read both questions to all respondents. Emphasize that we don't want to know WHICH SPECIFIC statements are true but rather HOW MANY statements are true. It might be helpful for the respondent to count the true statements on their fingers behind their back to keep it a secret.		
D19.	HOW MANY of the following statements truthfully describe your life in the **last twelve months**? Please do not tell me which statements are true, just HOW MANY.<br><br>1. I became a father to a new baby.<br>2. I attended a wedding.<br>3. I received some health service at a hospital.<br>4. I purchased new clothing for myself.	└─┴─┘ statements
D20.	HOW MANY of the following statements truthfully describe your life in the **last twelve months**? Please do not tell me which statements are true, just HOW MANY.<br><br>1. I became a father to a new baby.<br>2. I attended a wedding.<br>3. I received some health service at a hospital.<br>4. I purchased new clothing for myself.<br>5. I slapped my wife or threw something at her that could hurt her.	└─┴─┘ statements
D21.	Have you ever heard of any campaigns or activities in your community or workplace that talk about preventing violence against women?	1. [ ] Yes<br>2. [ ] No<br>3. [ ] Don't know
D22.	Have you ever seen an advertisement or public service announcement on television about violence against women?	1. [ ] Yes<br>2. [ ] No<br>3. [ ] Don't know
D23.	Have you ever participated in an activity (group session, rally, etc.) in your community or workplace on violence against women?	1. [ ] Yes<br>2. [ ] No<br>3. [ ] Don't know
NOTE TO ENUMERATOR: For the next two questions – respondents should only be asked **ONE question** depending on their respondent ID number.<br><br>Emphasize that we don't want to know WHICH SPECIFIC statements are true but rather HOW MANY statements are true. It might be helpful for the respondent to count the true statements on their fingers behind their back to keep it a secret.<br><br>If the last digit of the respondent ID number is **odd**, then read questions **D24 ONLY.**<br><br>If the last digit of the respondent ID number is **even**, then read question **D25 ONLY.**		

D24.	HOW MANY of the following statements truthfully describe your life in the **<u>last twelve months</u>**? Please do not tell me which statements are true, just HOW MANY.<br><br>1. I have been to Addis.<br>2. My brother visited my home.<br>3. I listened to the radio almost every day.<br>4. I watched a football match.	└─┴─┘ statements
D25.	HOW MANY of the following statements truthfully describe your life in the **<u>last twelve months</u>**? Please do not tell me which statements are true, just HOW MANY.<br>1. I have been to Addis.<br>2. My brother visited my home.<br>3. I listened to the radio almost every day.<br>4. I watched a football match.<br>5. I used physical violence such as hitting, slapping, pushing or threats of violence against my wife.	└─┴─┘ statements

Enumerator: Now, I am going to read you a list of statements. For each statement, I would like you to first tell me if you agree with this statement yourself, and next tell me if you think a majority (more than half) of your friends and family agree with this statement. For example, I could read the statement "It is beneficial for all children to attend primary school." First, you would say "Yes" if you agree with this statement yourself. Next, if you believe that the majority of your friends and families also agree, you would say "Yes" again.

		(a) Do you agree with this statement?	(b) Do your friends and family agree with this statement?
D26.	A man should have the final word on decisions in his home.	1. [ ] Agree<br>2. [ ] Partially agree<br>3. [ ] Do not agree	1. [ ] Yes, Agree<br>2. [ ] Partially agree<br>3. [ ] Do not agree<br>4. [ ] Don't know
D27.	A woman should obey her husband in all things.	1. [ ] Agree<br>2. [ ] Partially agree<br>3. [ ] Do not agree	1. [ ] Agree<br>2. [ ] Partially agree<br>3. [ ] Do not agree
D28.	It is alright for a man to beat his wife if she is unfaithful.	1. [ ] Agree<br>2. [ ] Partially agree<br>3. [ ] Do not agree	1. [ ] Agree<br>2. [ ] Partially agree<br>3. [ ] Do not agree
D29.	A man can hit his wife if she won't have sex with him.	1. [ ] Agree<br>2. [ ] Partially agree<br>3. [ ] Do not agree	1. [ ] Agree<br>2. [ ] Partially agree<br>3. [ ] Do not agree

D30.	A woman should not initiate sex.	1. [ ] Agree<br>2. [ ] Partially agree<br>3. [ ] Do not agree
D31.	A man should be outraged if his wife asks him to use a condom.	1. [ ] Agree<br>2. [ ] Partially agree<br>3. [ ] Do not agree

D32.	It is a woman's responsibility to avoid getting pregnant.	1. [ ] Agree<br>2. [ ] Partially agree<br>3. [ ] Do not agree
D33.	A woman who has sex before she marries does not deserve respect.	1. [ ] Agree<br>2. [ ] Partially agree<br>3. [ ] Do not agree

D34.	Women should not tolerate violence just in order to keep her family together.	1. [ ] Agree<br>2. [ ] Partially agree<br>3. [ ] Do not agree
D35.	There are times a woman deserves to be beaten.	1. [ ] Agree<br>2. [ ] Partially agree<br>3. [ ] Do not agree
D36.	A man using violence against his wife is a private matter that shouldn't be discussed outside the couple.	1. [ ] Agree<br>2. [ ] Partially agree<br>3. [ ] Do not agree
D37.	It disgusts me when I see a man acting like a woman.	1. [ ] Agree<br>2. [ ] Partially agree<br>3. [ ] Do not agree

FO: Now, some people may believe that a man has a good reason to hit his wife in certain situations. I am going to read you a list of situations. For each one, please tell me if you believe that a man has a good reason to hit his wife in this situation. Please also tell me if you think most of your friends and families agree that a man would have a good reason to hit his wife in this situation.

	(a) Do you agree a man would have a good reason to hit his wife in this situation?	
D38.	She disobeys him.	1. [ ] Yes<br>2. [ ] No
D39.	She answers back to him.	1. [ ] Yes<br>2. [ ] No
D40.	She neglects taking care of the children.	1. [ ] Yes<br>2. [ ] No
D41.	She burns the food.	1. [ ] Yes<br>2. [ ] No
D42.	She refuses to have sex with him.	1. [ ] Yes<br>2. [ ] No
D43.	She spends time gossiping with neighbors.	1. [ ] Yes<br>2. [ ] No
D44.	She ignores taking care of children.	1. [ ] Yes<br>2. [ ] No
D45.	She is not performing what husband is expecting.	1. [ ] Yes<br>2. [ ] No
D46.	She doesn't trust the husband and argues.	1. [ ] Yes

		2. [ ] No
D47.	She spreads the news about her husband to neighbors.	1. [ ] Yes<br>2. [ ] No
D48.	He is mad at his wife.	1. [ ] Yes<br>2. [ ] No
D49.	She is not making tasty food.	1. [ ] Yes<br>2. [ ] No
D50.	She leaves the house without the permission of her husband.	1. [ ] Yes<br>2. [ ] No

Section E.

a) Knowledge about HIV/AIDS

E1.	Please mention all of the ways you believe a person can get infected with HIV/AIDS.<br><br>***Do NOT read the list, tick off all that are mentioned.***	1. [ ] Sexual intercourse<br>2. [ ] Sharing needles (drug use)<br>3. [ ] Unclean medical equipment<br>4. [ ] Blood transfusions<br>5. [ ] During pregnancy<br>6. [ ] During birth<br>7. [ ] Through breast milk<br>8. [ ] Mosquito/insect bites<br>9. [ ] Contact with blood of infected person<br>10. [ ] Contact with infected person's toothbrush<br>11. [ ] Casual contact with infected person (i.e. sharing food, cup, glass, handshake, hugging, clothes)<br>12. [ ] Other (specify)____ ______________________<br>13. [ ] Has not heard about HIV/AIDS<br>14. [ ] Don't know/Don't remember

E2.	Can you tell me all the ways you know of that people can protect themselves from HIV?<br><br>***Tick all that are mentioned.***	1. [ ] Abstinence<br>2. [ ] Being faithful<br>3. [ ] Using condoms<br>4. [ ] Using condoms correctly and consistently<br>5. [ ] Going for VCT before engaging in sex<br>6. [ ] Not sharing sharp objects<br>7. [ ] Avoiding drugs/alcohol/anything which hampers judgment<br>8. [ ] Avoiding bad company<br>9. [ ] Avoiding prostitution<br>10. [ ] Avoiding walking alone at night<br>11. [ ] Avoiding contact between bloody wounds and skin<br>12. [ ] Avoiding circumcision with unsafe tools<br>13. [ ] Avoiding wife-inheritance<br>14. [ ] Avoiding sugar daddies/mummies<br>15. [ ] Avoiding multiple sexual partners<br>16. [ ] Voluntary Male circumcision<br>17. [ ] Other (specify___________________________<br>18. [ ] Don't know/ Don't remember

E3.	Can people reduce their chance of getting the AIDS virus by having just one uninfected sex partner who has no other sex partners?	1. [ ] YES<br>2. [ ] NO<br>3. [ ] Don't Know
E4.	Can people get the AIDS virus from mosquito bites?	1. [ ] YES<br>2. [ ] NO<br>3. [ ] Don't Know
E5.	Can people reduce their chance of getting the AIDS virus by using a condom every time they have sex?	1. [ ] YES<br>2. [ ] NO<br>3. [ ] Don't Know
E6.	Can people get the AIDS virus because of witchcraft, God's curse, or other supernatural means?	1. [ ] YES<br>2. [ ] NO
E7.	Do you think that a healthy-looking person can have HIV?	1. [ ] YES<br>2. [ ] NO<br>3. [ ] Don't Know

b) Attitudes about HIV/AIDS

E8.	If a member of your family got infected with the AIDS virus, would you want it to remain a secret or not?	1. [ ] YES, remain a secret<br>2. [ ] NO<br>3. [ ] Don't Know/not sure/depends
E9.	If a member of your family got infected with the AIDS virus, would you be willing to care for her or him in your own household?	1. [ ] YES<br>2. [ ] NO<br>3. [ ] Don't Know/not sure/depends

E10.	Would you buy fresh vegetables from a shopkeeper or vendor if you knew that the person had the AIDS virus?	1. [ ] YES<br>2. [ ] NO<br>3. [ ] Don't Know
E11.	In your opinion, if a female teacher has the AIDS virus but is not sick, should she be allowed to continue teaching in the school?	1. [ ] YES<br>2. [ ] NO<br>3. [ ] Don't Know
E12.	I don't want to know the results, but have you ever had a blood test for HIV?	1. [ ] YES<br>2. [ ] NO

c) Behavior Questions

FO: Now I am going to ask you some more questions. These ones will be about some topics that may seem embarrassing. Again, this is totally confidential and I won't share this information with anyone else.

E13.	In your **lifetime**, how many sexual partners have you had?<br>Adding the current spouse	|__|__| partners

E14.	In the **<u>last 12 months</u>**, how many sexual partners have you had?<br>Adding the current spouse	|__|__| partners
E15.	Have you **<u>ever</u>** used a condom?	1. [ ] YES<br>2. [ ] NO **>>>>>>>> skip to question E17**
E16.	Did you use a condom **<u>last time you had sex</u>**?	1. [ ] YES<br>2. [ ] NO<br>3. [ ] Not sure
E17.	How confident are you that you could convince your partner to use a condom if you wanted to use one?	1. [ ] Not at all confident<br>2. [ ] Somewhat confident<br>3. [ ] Confident<br>4. [ ] Very confident
E18.	How confident are you that you know how to correctly use a condom?	1. [ ] Not at all confident<br>2. [ ] A little confident<br>3. [ ] Somewhat confident<br>4. [ ] Confident<br>5. [ ] Very confident
E19.	In the last 12 months, have you ever discussed HIV risk behaviors with your partner?	1. [ ] YES<br>2. [ ] NO
E20.	In the last 12 months, have you ever discussed sex with your partner?	1. [ ] YES<br>2. [ ] NO

F. Program Questions

F1.	Do you remember the program “Unite for a Better Life,” a series of discussions and coffee ceremonies held in this community in 2015?<br><br>**If no- probe to make sure they don’t remember the program “Unite for Life is a program that brought people together in the community to discuss healthy relationships and gender issues”**	1. [ ] Yes<br>2. [ ] No **>>>>> SKIP TO G1**
F2.	How many of these discussions did you attend?	|__|__| Sessions
F3.	If attended less than 14 sessions, why did you not attend any/ more sessions?	1. [ ] Subject matter not interesting<br>2. [ ] Time not available (had other commitment)<br>3. [ ] Did not feel comfortable discussing those topics<br>4. [ ] Feared the disapproval of spouse<br>5. [ ] Feared the disapproval of other members of the community<br>6. [ ] Was not invited/selected for participation<br>7. [ ] Did not feel comfortable with the facilitator<br>8. [ ] Other

F4.	Did you attend the discussions alone or with your spouse?	1. [ ] Always Alone<br>2. [ ] Always with my spouse<br>3. [ ] Sometimes with my spouse<br>4. [ ] Didn't attend any sessions
F5.	How many sessions did your spouse attend?	└─┴─┘ Sessions
	**Enumerator: Check question F2 and copy response here:**<br><br>**# of sessions attended by the respondent └─┴─┘ Sessions IF = 0 then SKIP TO F16**	
F6.	If you did attend these discussions, would you say you remember most of what was discussed, some of what was discussed, only a little, or nothing at all?	1. [ ] Most or nearly all of what was discussed<br>2. [ ] Some of what was discussed<br>3. [ ] Little of what was discussed<br>4. [ ] Nothing at all [**Skip to F12**]
F7.	What was the most important information you discussed in these sessions?	______________________________<br>______________________________
F8.	Did you share the information you obtained from this program with anyone?	1. [ ] Yes<br>2. [ ] No **>>>>> SKIP to F12**
F9.	If yes, who did you share the information with?<br><br>Tick all that apply	1. [ ] Spouse<br>2. [ ] Relative<br>3. [ ] Friend<br>4. [ ] Neighbor<br>5. [ ] My children<br>6. [ ] Other (specify)
F10.	How many people in total did you share the information with?	└─┴─┘ people
F11.	On a scale of 1 to 5, how satisfied were you with the program?	1. [ ] Not at all satisfied<br>2. [ ] A little satisfied<br>3. [ ] Somewhat satisfied<br>4. [ ] Satisfied<br>5. [ ] Very satisfied
F12.	Were there any negative consequences of participating in these sessions?	1. [ ] Yes<br>2. [ ] No >>>>>> SKIP to F14
F13.	If yes, what were the negative consequences of participating in these sessions?<br><br>**Tick all that apply.**	1. [ ] Lost wages<br>2. [ ] No time to do household chores<br>3. [ ] Angered spouse when I participated<br>4. [ ] Spouse became violent when I participated<br>5. [ ] Angered family members when I participated<br>6. [ ] Family members became violent when I participated

		7. [ ] Stigma from community members (outcast by others, name calling, gossip about you)<br>8. [ ] Other (specify) ________________
F14.	How likely would you be to recommend to a friend to participate in this program?	1. [ ] Not at all likely<br>2. [ ] A little likely<br>3. [ ] Somewhat likely<br>4. [ ] Likely<br>5. [ ] Very likely
F15.	Do you continue to meet the members of your Unite for a better life group to continue to discuss topics related to the program?	1. [ ] Yes<br>2. [ ] No<br><br>**>>>>>SKIP TO G1**
	**QUESTIONS F16-F18 ARE ONLY FOR THOSE WHO DID NOT PARTICIPATE IN ANY SESSIONS (where the response to question H2=0)**	
F16.	Did anyone share information from the Unite for a better life program discussions with you?	1. [ ] Yes<br>**2. [ ] No >>>>>>>>>>>>>>SKIP TO G1**
F17.	If yes, who shared the information with you?<br><br>**Tick all that apply.**	1. [ ] Spouse<br>2. [ ] Relative<br>3. [ ] Friend<br>4. [ ] Neighbor<br>5. [ ] Other (specify)
F18.	Which specific information did they share with you?	____<br><br>____

G. Spouse background information, Employment status

G1.	In the last 12 months, what has been your average monthly income?	|__|__|__| Birr
G2.	Have you ever worked or done any activities for income?	1. [ ] Yes<br>2. [ ] No **>>>>>SKIP TO G5**
G3.	What kind of work or activities to earn income have you done in the last 12 months?	___________________
G4.	What was the main reason you worked extra time in the last 12 months?	1. [ ] For my family<br>2. [ ] For myself<br>3. [ ] For others
G5.	When did you do the extra work?	1. [ ] The whole year<br>2. [ ] Occassionally<br>3. [ ] Sometimes
G6.	What did you do to earn extra in the past 12 months?	1. [ ] Cash<br>2. [ ] In kind

		3. [ ] In both<br>4. [ ] Was not paid at all
G7.	In the last 12 months what was your average daily wage?<br>If in kind paid, change to a monetary	|__|__|__| Birr
G8.	Who usually decides how the money you earn will be used: mainly you, mainly your wife/ partner or you and your wife/partner jointly?	1. [ ] Respondent<br>2. [ ] Wife/partner<br>3. [ ] Respondent and wife/ partner jointly<br>4. [ ] Other______________
G9.	Would you say that the money that you earn is more than what your wife/partner earns, less than what she earns or about the same?	1. [ ] More than her<br>2. [ ] Less than her<br>3. [ ] About the same<br>4. [ ] wife/partner doesn't bring in money<br>5. [ ] Don't know
G10.	Who usually decides how your wife's/ main partner's earnings will be used: you, your wife/partner, or you and your wife/partner jointly?	1. [ ] Respondent<br>2. [ ] wife/ partner<br>3. [ ] Respondent and wife/partner jointly<br>4. [ ] Other______________

H1. Anonymous picture technique

I would now like to give you a card with photos. On this card are two pictures, but no other message. The first picture is of a happy face, the second is of a sad face.

No matter what you have already told me, I would like you to put a mark next to the sad picture if you have committed any act of physical physical violence such as hitting, slapping, pushing or threats of violence or sexual violence against your wife in the last 12 months.. Please put a mark next to the happy face if this has never happened.

Once you have marked the card, please fold it over and put it in an envelope and then on this bag, along with many other responses from this village. This will ensure that I do not know your answer. Your response will remain completely anonymous and there is no way for anyone to know what your answer is. Your confidentiality will be protected, so please feel free to share the honestly without fear.

GIVE RESPONDENT CARD AND PEN. DO NOT LOOK AT RESPONSE. ONCE CARD IS FOLDED, ASK REPONDENT TO PUT IT IN AN ENVELOPE AND INTO A BAG THAT ALSO CONTAINS OTHER COMPLETED CARDS. DO NOT RECORD DETAILS OF QUESTIONNAIRE IDENTIFICATION ON CARD.

The End

Note:

Before leaving the participant

- **Check for consistency and completeness of each question**
- **Provide greetings and thank him.**
